# Shape Guides Visual Pretense

**DOI:** 10.1162/OPMI.a.277

**Published:** 2025-12-18

**Authors:** Peng Qian, Tomer D. Ullman

**Affiliations:** Department of Psychology, Harvard University, Cambridge, MA, USA; Kempner Institute for the Study of Natural and Artificial Intelligence, Harvard University, Cambridge, MA, USA

**Keywords:** pretense, imagination, multi-modal model, image inpainting

## Abstract

People often imagine everyday objects are something else. A turned over bottle becomes a car, a teapot becomes a swan. Such pretense is common in play, pedagogy, and narratives. The relationship between a real and pretend object is flexible, but not arbitrary. In this work, we used a behavioral and computational approach that compares people and performant multi-modal vision models to study the features that guide the construction of visual pretense. In four studies (*N* = 716 in total), we show that people have systematic preferences in visual pretense, and that these preferences are better accounted for by spatial and physical alignment (specifically shape similarity), over surface feature similarity (such as color). We also found that people systematically align the subpart structure of real and pretend objects. We further show that people’s visual pretense preferences are not accounted for by current common approaches to multi-modal vision models, likely due to their reliance on surface features rather than spatial and physical ones.

## INTRODUCTION

Suppose you wanted to play pretend. Looking around, you notice a red block, like the one below. Would it make more sense to pretend that this block is a *car*, or a *strawberry*?


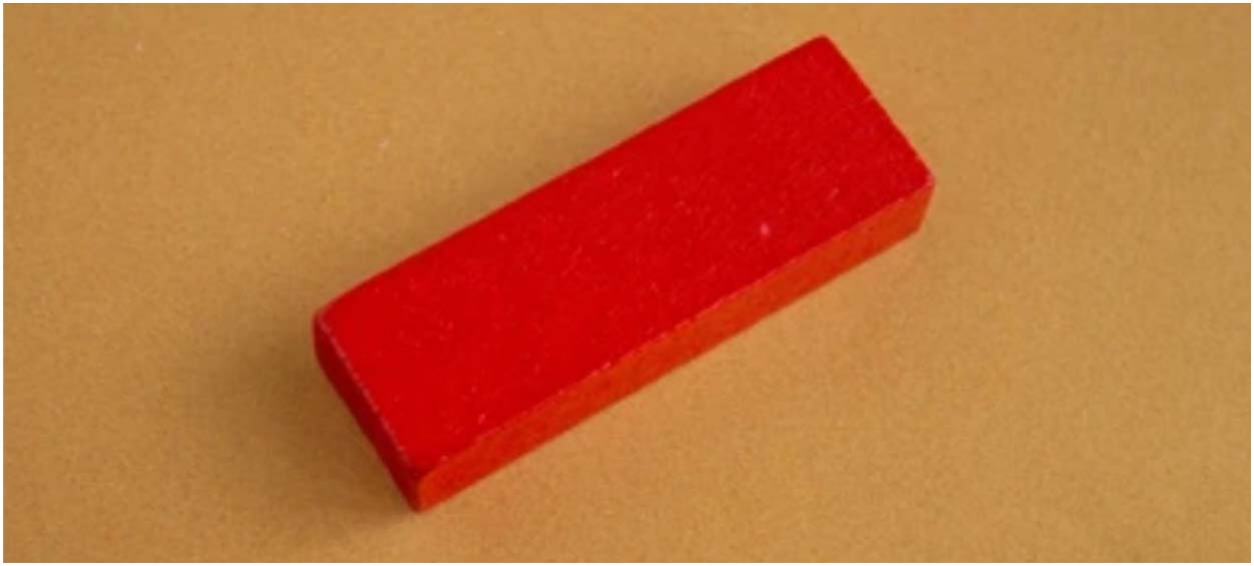


As it turns out, a majority of people answer “car”. But why? After all, pretense is a departure from reality, and in principle anything can be pretended to be anything else (Currie & Ravenscroft, 2002). To imagine a block as a strawberry is just as reasonable in theory as imagining it to be a car, or a cart, or a carburetor. However, a great deal of work in cognitive science, development, and philosophy suggests that imagined possibilities are constrained by our understanding of the real world (Byrne, 2007; Harris, 2000; McCoy & Ullman, 2019; Nichols, 2006; Shtulman, 2023, *inter alia*). Such accounts predict that some imagined possibilities will make more sense than others. But even taking such accounts into account, it doesn’t immediately follow what specific features of the real world constrain pretense, such that it makes more sense to pretend that the block is a car.

While much of the previous work on imagination and pretense in cognitive science and philosophy has focused on vignettes, the specific kind of pretense we have in mind here is *visual pretense*. This is the everyday activity demonstrated by the opening example, the act that underlies swinging a stick while making light-saber sounds, or grabbing a bottle and bottle cap to explain “here’s me, and here’s the truck that nearly ran me over”. This intuitive behavior is common-place in both children and adults, and shows up in everyday behaviors including communication, explanation, play, and pedagogy. Certainly such behavior has been studied in cognitive development (see e.g., Gleason & White, 2023; Harris, 2000; Lillard, 2017; Lockman & Tamis-LeMonda, 2021), and a great deal of work has focused on when children are able to use objects as symbols, and the degree to which iconic mapping can aid in their reasoning (e.g., DeLoache, 2010; Tomasello et al., 1999). A line of developmental literature on pretend play has also noted the perceptual similarity between an object and its intended referent in play, which is different from the arbitrariness of linguistic symbols (Harris et al., 1993; Piaget, 1945; Vygotsky, 1967). Studies on object substitution in pretend play suggests that young children more readily accept and engage with pretend activity when highly prototypical substitution is involved (Elder & Pederson, 1978; Fein, 1975; Jackowitz & Watson, 1980; Rabkina & Forbus, 2022), that children prefer choosing generic objects with matching shape to the intended referent (Burns-Nader et al., 2019), and that younger children find it more difficult to comprehend another person’s pretense when the real object is dissimilar in form to the pretend object as implied by a specific pretend action (Bigham & Bourchier-Sutton, 2007; Hopkins et al., 2016).

So, the role of overall object similarity in pretend play has been long observed and discussed. But, to our knowledge, there is no systematic and quantitative account that details which similarity features (if any) guide the widespread and intuitive act of visual pretense, including into adulthood. Going back to our opening question: it sure seems to make more sense that the block is a car, but why?

In this work, we systematically investigate the idea that while in principle pretense can take any object to stand in for any other, in practice people have principled preferences in visual pretense. We suggest that these preferences are guided by an alignment between real-world objects and pretense objects. More specifically, we argue that physical and spatial alignment matters more for this pretense than surface similarity. We emphasize early (and discuss later) that we do not mean that surface features do not matter, and also that feature-weighting may well be context-sensitive.

The notion that physical, spatial alignment will matter more for visual pretense than surface features parallels the contrast between structural and surface level features in other processes. As one prominent example, infants are sensitive to shape similarity for inductive inference (Graham & Diesendruck, 2010; Graham et al., 2004). Decades of research in cognitive development has documented a ‘shape bias’ in early language learning: children tend to extend a novel word based on shape, rather than things like color or material (Landau et al., 1988; Samuelson & Smith, 2000). This phenomenon exists in early word learning, and also extends into children’s categorization (Gelman, 2003). While there are differing theoretical accounts for this tendency (and see for example Diesendruck & Bloom, 2003; Portelance et al., 2021; Samuelson & Smith, 1999; Smith, 1999; Xu & Tenenbaum, 2007), its overall robustness suggests that object shape (alongside other physical properties) may be a foundational property in other cognitive processes.

Despite the importance of physical features like shape in processes such as word learning and conceptual generalization, there are reasons to think that surface features like color and texture will play a more dominant role in pretense. First of all, visual pretense is not a learning task. Adults and children that engage in visual pretense are not labeling new instances of a category (the bottle is not actually a sword), and it’s not immediately obvious that a bias useful for learning (getting the world right) will be relevant in imagining (coming up with a new world). Second, surface features such as color do matter for a variety of cognitive processes, including recognition (Price & Humphreys, 1989; Rossion & Pourtois, 2004; Tanaka et al., 2001). The stronger the association between object and color, the stronger the influence of color on recognition (Tanaka & Presnell, 1999). Similar effects have been found for visual scene recognition (Oliva & Schyns, 2000). Third, many current successful machine models of visual processing rely on statistics extracted from large-scale datasets of naturalistic images, without necessarily representing the physical and spatial features in an explicit or salient manner (Geirhos et al., 2019; Hermann et al., 2020; Ritter et al., 2017), including cutting-edge image generation models. Given all this, it seems at least *a priori* reasonable that people may rely on information about the surface-level visual appearance (or various features as a holistic representation) in visual pretense.

**Figure 1.**
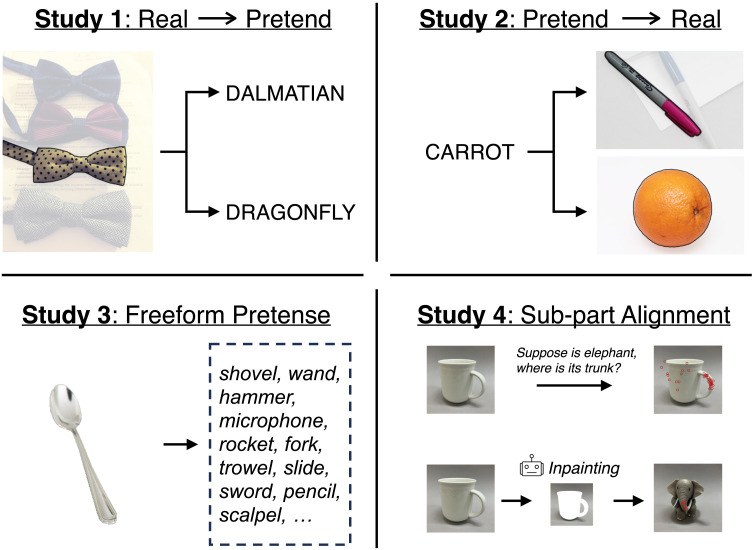
Overview of the four studies. All studies collected data on pretense preference from human participants, and compared them both to human judgments of similarity, as well as to generative multi-modal AI models.

To test our proposal that people have systematic preferences in visual pretense, and that these preferences are guided primarily by physical features, we designed four pre-registered studies (see Figure 1 for a basic overview). Studies 1–3 aimed to measure people’s visual pretense preferences, and correlated this preference with human-based judgments of shape and color similarity, as representatives of major physical and surface-level features. These studies also compared people’s pretense preferences to the similarity judgments of current language-and-vision AI models. The use of AI models allows us to capture in aggregate a great deal of visual similarity within a learned general-purpose embedding space. Moving beyond general pretense preferences, Study 4 examined the idea that people align specific sub-parts of imagined and real objects when engaging in visual pretense, and compared people’s behavior to contemporary generative multi-modal AI systems. Across all four studies, we found converging evidence that shape similarity between a real object and a pretense target highly predicts people’s preferences in visual pretense, suggesting that people’s systematic preferences in visual pretense are guided by structural and spatial alignment between the real and the pretend object. Surface similarity in the form of color features hardly predicts visual pretense at all. Moreover, we compared our results to three representative and performant contemporary multi-modal foundation models in AI, and found that despite the rich statistical regularities learned from gigantic data of images and texts, representations derived in these models still fall short as an adequate model of the way people engage in visual pretense. On a broader level, our findings contribute to a more precise account of how people flexibly re-purpose physical objects as representations of ideas in the mind.

## METHODS

### Study 1: Real → Pretend

#### Stimuli and Materials.

We created a set of 50 images, each highlighting a different everyday object. The objects varied along many visual dimensions. The original images were selected from Open Images Dataset V7 (Krasin et al., 2017) and LVIS (Gupta et al., 2019). As some images contained more than one object, all the images were pre-processed to highlight the target object and fade away the background, based on the annotated object mask in the image database. Each image was paired with two words that describe different entities. For example, a picture of a bow-tie was paired the options “Dalmatian” and “dragonfly”, as shown in Figure 1. The 50 triads (image + word pairs) form stimuli set in Study 1. The words were chosen with the heuristics that two pretense objects may share certain spatial/physical or surface-level features with the target real object to varying degrees. We note that while this approach to stimuli design aims for convenience and broader coverage, future studies can explore alternative possibilities for more precise control over the stimulus set, such as using computer graphics to render images of familiar or novel objects with specific feature combinations.

#### Procedure and Participants.

There were three different tasks in Study 1, each completed by a different random sample of participants. In the Pretense task, participants saw the 50 images of different everyday objects from the stimuli set, one at a time, in random order. For each image, participants were asked to select the best completion for “*It makes more sense to pretend the* [object in the picture] *is a …*”, given a forced choice between the pair of words. For example, participants saw an image highlighting a mug, and were asked to indicate whether it makes more sense to pretend the mug is an *elephant* or a *caterpillar*. The Shape task was identical in design to the Pretense task, except that Participants were asked to select the best completion for “*The shape of the* [object in the picture] *is more similar to …*”. The Color task was again identical to the Shape task, except participants were asked to select the best completion for “*The color of the* [object in the picture] *is more similar to …*”. This study, together with Study 2–4, was approved by the Institutional Review Board of Harvard University. All participants of the human behavioral studies gave informed consent.

We recruited 120 participants online via Prolific (*M*_*age*_: 35.7; 50.8% female, 49.2% male; 5.8% Asian, 9.2% Black, 5.0% Mixed, 76.7% White, 1.7% Other, 1.7% did not report), 40 for each task. All participants were native English speakers based on the United States. The study was pre-registered on Open Science Framework (https://osf.io/xzvhc). In the Results section, we report a set of correlation analyses, as their measures across conditions map onto the prediction of our hypotheses. We also pre-registered and conducted mixed-effect logistic regression analyses of trial-level data for Study 1–3, and found qualitatively similar patterns consistent with our predictions (see Section A in Supplemental Information).

In addition to the color comparisons, we wanted to jointly examine a host of visual features. We considered a set of current leading language-vision foundation models in AI, including CLIP (Radford et al., 2021), ALIGN (Jia et al., 2021), and FLAVA (Singh et al., 2022). These models differ in their particulars but together they form a good basis for comparison as they are representative instances of multi-modal embedding space learned from gigantic amounts of text-image pairs. The joint image-text embedding space of the contemporary multi-modal foundation models is a useful way of operationalizing the hypothesis that people’s visual pretense preferences simply extend the representational similarity structure that one has acquired from naturally paired visual and linguistic input. Compared to the classic neural network models of object recognition, including AlexNet (Krizhevsky et al., 2017) and ResNet (He et al., 2016), these Transformer-based foundation models are shown to generalize more robustly and less biased towards texture (Geirhos et al., 2021). Internal representations extracted from models like CLIP have also been shown to effectively predict human similarity judgment of natural objects (Kaniuth et al., 2024).

Specifically, we used the pre-trained model checkpoints (openai/clip-vit-base-patch32 for CLIP, kakaobrain/align-base for ALIGN, and facebook/flava-full for FLAVA) via Huggingface (Wolf et al., 2020). We derived representational similarities from these models. Similar to the structure of the Pretense task, each model received the same image that highlights a specific real object as human participants had seen in the behavioral experiment, together with two text items. For example, the model took the image of a bow-tie, as well as the text items “*A Dalmation*” and “*A dragonfly*” as input, transformed them into a high-dimensional embedding space, and computed a score over each pair of the text input and the visual input, which can be transformed through a softmax function into a probability distribution over the two phrases “*A Dalmation*” and “*A dragonfly*” given the input image of a bow-tie. This distribution was the basis of the comparison for people’s pretense preference. In our results in the main paper and main figures, we mostly discuss CLIP as a simple and convenient instance and to avoid visual clutter. We note that the results for the other models are similar, and that the full findings for ALIGN and FLAVA are presented in the Supplemental Information. Also note that we did not consider visual instruction-tuned models (e.g., LLaVa; Liu et al., 2024) as they interact with humans through chat, which introduces unnecessary complexities, and makes it less straightforward to compute the similarity measure in the model’s internal representations. While some studies fine-tuned pretrained models on human behavioral data (Binz et al., 2025; Muttenthaler et al., 2023; Zalcher et al., 2025), we chose to use the off-the-shelf foundation models so that we can more clearly assess the explanatory potential of multi-modal contrastive learning as a possible account of the source of human visual pretense preferences.

### Study 2: Pretend → Real

#### Stimuli and Materials.

We created a set of 50 objects specified as text. Each object was paired with two visually-depicted real objects. The images of the real objects were selected from Open Images Dataset V7 (Krasin et al., 2017) and LVIS (Gupta et al., 2019), and largely overlapped with the images in Study 1. For example, the text item “ladder” was paired with an image that highlighted a red high-heel shoe, and and an image that highlighted a wooden baseball bat. The 50 triads (word + image pairs) form the stimuli set for Study 2.

#### Procedure and Participants.

As with Study 1, there were three tasks in Study 2. In the Pretense task, participants were presented with 50 word-image triads, one at a time, in random order. for each item, participants were shown images of two everyday real-world objects, together with the text prompt “*Suppose someone wants to pretend that something is* [text item]*. Would it make more sense to pretend that the* [real object 1] *is* [text item]*, or the* [real object 2] *is* [text item]*?*”. Participants were asked to make a forced-choice between the two real objects highlighted in the images. The ordering of the images was randomized. The key dependent variable was people’s preference for the real objects (e.g., the choice of apple or banana given the prompted text target “a phone”). The Shape task was identical to the Pretense task, except that participants responded to the prompt “*Which of the following is more similar to* [text item] *just in terms of shape*, [real object 1] *or* [real object 2]*?*”. The Color task was again identical to the Shape task, except that participants were asked about color rather than shape.

In total, we recruited 120 participants online via Prolific for Study 2 (*M*_*age*_: 36.7; 49.2 female, 48.3% male, 2.5% did not report; 5.8% Asian, 8.3% Black, 7.5% Mixed, 67.5% White, 7.5% Other, 3% did not report), 40 for each task. All the participants were native English speakers based on the United States. Data from one participant in the Shape task and one participant in the Color task were excluded in the analyses due to failure at passing the attention check. The study was pre-registered on the Open Science Framework (https://osf.io/k8uya).

As with Study 1, we again derived representational similarities from CLIP, ALIGN, and FLAVA (details identical to Study 1). The model received the linguistic description of the target pretend object, together with the two images that highlighted specific real objects just as participants had seen in the behavioral experiment. For example, the model took the text “*A carrot*” together with images of a marker and an orange as input, mapped them into a high-dimensional embedding space, and computed a score over each pair of the text input and the visual input, which can then be transformed through softmax function into a probability distribution over the two images given the text input. We took the output distribution as the basis for comparing to participants pretense preference. Similar to Study 1, we mostly discuss CLIP as a simple instance and to avoid visual clutter in the plots. Results for the other models are similar, and the full findings for ALIGN and FLAVA are presented in the Supplemental Information.

### Study 3: Freeform Pretense

#### Stimuli and Materials.

We used the same set of images of 50 everyday objects as in Study 1. For each image, the target object was cropped out by masking the background completely as white pixels. This was done to minimize semantic association effects from the surrounding visual scene in the image during the elicitation of open-ended responses.

#### Procedure and Participants.

Participants were presented with the 50 images from the stimuli set in random order. For each image, participants were given the prompt “*What would it make sense to pretend the* [object in the picture] *is?*”. Participants typed in their answers as a free-form response, providing one answer per prompt, meaning 50 answers from each participant.

After eliciting free-form responses, we sub-sampled the space of pretend objects that participants generated, among the phrases that contain less than three words (these account for 93% of people’s free-form pretend responses). For each of the 50 real-world objects in the stimuli, we randomly sampled 3 pretend objects, weighted by the frequency of the objects (so, for example, if 4 people responded ‘shovel’ when seeing a spoon, and 1 person responded ‘paddle’, it was 4 times more likely that we would sub-sample the word ‘shovel’ for the spoon item). Note that the sampling process was run on the frequency distribution of distinctive lexical items in the elicited responses (i.e., ‘vanity mirror’ and ‘dental mirror’ were treated as different types while the referred concepts may have overlapping features). This produced 150 pairs of real object and pretense options. Next, we added to each real-image-and-pretense-word a control word, by randomly taking a word that was generated in response to a different real-world item. So, given a real object X1 and the sub-sampled pretense option Y1, and the real object X2 and the sub-sampled pretense option Y2, we paired X1 with [Y1, Y2] and X2 also with [Y1, Y2]. This process created 150 triads of [real object (image), matched pretense object (text), randomly-paired pretense object (text)].

Based on the 150 triads, a separate sample of participants completed an object feature judgment task similar to the structure of Study 1. Each participant saw a random subset of 50 triads from the 150 total amount, in random order. For each triad, participants made a forced-choice between the two pretend objects. In the Shape task, participants were asked to respond to the prompt “*The Shape of the* [object in image] *is more similar to* [pretense object 1] */* [pretense object 2]”. The Color task was identical, except that participants responded to a prompt asking about color rather than shape. The key dependent variable was the proportion of participants’ choice of the originally generated pretense object.

We recruited 280 online participants via Prolific for the pretense elicitation and feature judgment tasks (*M*_*age*_: 36.9; 46.1% female, 53.6% Male, 0.4% did not report; 7.1% Asian, 8.2% Black, 5.4% Mixed, 73.2% White, 3.2% Other, 2.9% did not report). All participants were native English speakers based in the United States. Of these, 40 were assigned to the free-form pretense elicitation task, 120 were assigned to the shape similarity judgment task (Shape), and 120 were assigned to the color similarity judgment task (Color). As mentioned, each participant in the shape/color judgment task was presented with 50 trials across the 50 real objects presented in one of the three possible triads that were paired in the aforementioned manner. The study was pre-registered on the Open Science Framework (https://osf.io/8c6yp).

### Study 4: Sub-Part Alignment

#### Stimuli and Materials.

We created our stimuli set by taking photos of everyday entities in the lab, specifically a bottle, a broccoli, a toy car, a mug, a pear, a stapler, a teapot, and a wineglass (see Figure 5). We took three photos of each object from different views. Each object was placed on a gray background, to create a sense of depth while keeping the details of the scene minimal. This formed 24 images in total (8 objects × 3 views).

#### Procedure and Participants.

There were two conditions in this study, Pretense and Prior. In the Pretense condition, participants were shown all 24 images, one at a time in random order. Each image was paired with a prompted pretend object and two sub-parts. For example, participants saw an image of a mug, prompted to pretend the mug was an elephant, then asked to click on the image of the mug where they thought the elephant’s trunk was, and then to click where the elephant’s left ear was. In total, each participant saw 48 trials, crossing 8 objects × 3 different views × 2 sub-parts. For each image, the two questions about the location of the subpart were presented consecutively, but in random order.

The design of the Prior condition was similar to the Pretense condition, except that the object in each images was covered by a centered dark gray square, whose size was just big enough to cover the object. This masking hid the structural layout of the real object in the original picture. Participants were prompted to imagine that there was an object behind the dark gray square, and then asked to click on the images to indicate where they thought the subpart of the pretend object is in their mind. This condition served as a control for the Pretense condition, to rule out the possibility that people’s systematic responses were due to prior biases in imagining an object regardless of the specific composition of the real-world object.

In addition to analyzing human behavior on its own, we compared human performance to state-of-art language-prompted generative vision models, as a stand-in for a specific way in which the ‘replacement’ of a real object by a pretend object could work in visual pretense. Models such as Stable Diffusion (Rombach et al., 2022) and DALL-E (Ramesh et al., 2022) have shown impressive performance in synthesizing novel and stylistic images given a linguistic prompt of the intended scene, and here we use them as candidate algorithmic accounts of human visual pretense. Conceptually, these models are trained to denoise images with the guidance of a linguistic prompt. It is possible that people’s imagination unfolds in a similar fashion: adding noise to the visual representation of the real object, and then denoising it to form a mental image of the pretend object. Inpainting with diffusion-based generative models implements an algorithm that enables exactly this type of editing operation over images. These diffusion models are flexible and open-ended. Specifically, the inpainting model pipelines we ran rely on a coarse-grained shape representation, i.e., the real object’s silhouette projected on the image, without an explicit representation of the fine-grained spatial layout of the real object. We compare how people and the inpainting AI models imagine a pretend object given an image of a real object, in terms of the subpart structure of the pretend object (as indicated by people and repainted directly by the AI models). We focus on the subpart structure as it helps reveal the format of people’s imagination by probing the details of *how* the pretend objects were held in the mind. The comparison between people and inpainting models would inform whether the process of human visual pretense could be adequately accounted for by simple guided edits over noisy imagery.

We ran the following image inpainting pipeline with contemporary generative language-vision models: we masked out the real objects in the image stimuli, and used the models to inpaint (‘fill in’) an object using the text prompt given to people as the pretense object. The object mask was created using the Segment Anything Model (SAM; Kirillov et al., 2023). The model-annotated object mask was smoothed and enlarged to ensure that all pixels of the object were masked out. During inpainting, for example, the model took as input a masked image of a mug, and filled in the masked-out mug given the prompt “*An elephant*”. We used two state-of-art diffusion-based generative image models, Stable Diffusion (Rombach et al., 2022) and DALLE-2 (Ramesh et al., 2022). Inpainting with Stable Diffusion used a pre-trained model (runwayml/stable-diffusion-inpainting) via Huggingface. Inpainting samples from DALLE-2 were accessed via OpenAI API. For each model, we randomly sampled 10 inpainted image for each original masked image. Both models generated images that were 512 × 512 pixels in size. The model-inpainted images were annotated by an independent sample of coders (*N* = 100) to indicate the location of the sub-parts of the prompted pretend object. Coders were told they would see images created by computational models. Each coder saw 24 model output images, one at a time. For each image, coders were prompted to indicate the location of two specific sub-parts of the object. For example, coders saw an image of a crocodile that DALLE-2 created by inpainting a stapler, and were asked to click on the image in response to the question “*Where is the tail of the crocodile in the image?*”. Each inpainted model image was annotated by 5 different coders.

We recruited a total of 200 participants online via Prolific (*M*_*age*_: 37.5; 49.0% female, 50.0% Male, 1% did not report; 6.0% Asian, 11.0% Black, 6.5% Mixed, 71.5% White, 0.5% Other, 4.5% did not report), 50 for the Pretense condition, 50 for the Prior condition, and an additional 100 participants for annotating the inpainting output from the generative language-vision models. Data from one participant in the Prior condition and one participant in the coding task were excluded in the analysis due to not passing an attention check. All the participants were native English speakers based in the United States. The image stimuli in this study were in the shape of a square and were presented to participants in the size of 512 × 512 pixels among different task conditions as well as in the coding procedure for the inpainted images. The study was pre-registered on the Open Science Framework (https://osf.io/9c24p).

## RESULTS

**Figure 2.**
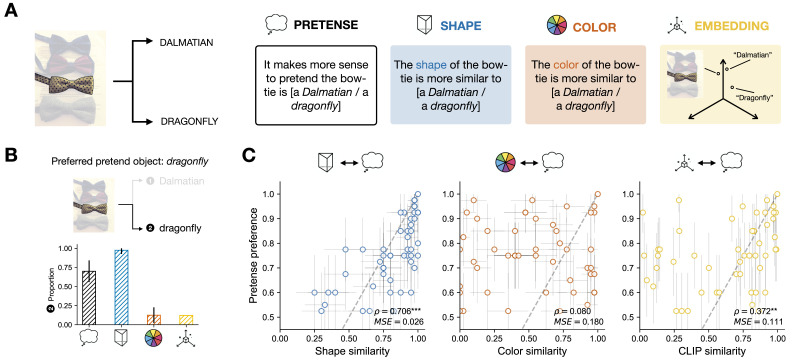
(A) Overview of Study 1, different groups of participants were presented with 50 images, and made a paired forced-choice between item pairs according to 3 different measures (pretense, shape, color). We also considered a similarity measure based on distances in a high-dimensional multi-modal space using CLIP (Radford et al., 2021). (B) Example from one item, across the different measures. (C) Correlation between pretense preferences and participant-based similarity measures of shape and color, as well as representational similarity derived from CLIP (results of other models are included in the Supplemental Information). The dependent measures (choice proportion) for the two given options of each item are inherently center-symmetrical across both ends of the dotted-line, and see the Supplemental Materials for plots showing the dis-preferred options. Spearman correlation (*ρ*) across items is based on the strength of preference for the preferred pretend options. Error bars indicate 95% confidence intervals with normal approximation.

We conducted a series of pre-registered studies to test the relationship between people’s visual pretense preferences and different object features. All the materials, data, and code are available in the following Open Science Framework repository: https://osf.io/ykdr5. In Study 1 (Real → Pretense, see Figure 2), people were given a task very similar to the opening example of this paper: participants were shown images of 50 everyday objects (e.g., an image of a bow-tie), and asked which object it would make more sense to pretend the real object was, given a forced pairwise choice (e.g., Dalmatian vs. dragonfly). In addition to this Pretense condition, we had two other independent groups of participants carry out norming tasks, which measured the degree to which people saw the real and pretend objects as similar in Shape or Color. To test our specific hypothesis about the relative importance of different features for visual pretense, we examined the fine-grained, item-specific patterns of correlation between features and pretense preference. As Figure 2C shows, shape similarity preferences were highly correlated with pretense preferences (Spearman *ρ* = 0.706, *p* = 1.00 · 10^−8^, *MSE* = 0.026), meaning that the greater the shape similarity between the real and pretense objects, the stronger the pretense preference between them. By contrast, color similarity was not predictive of pretense preferences (Spearman *ρ* = 0.08, *p* = 0.57, *MSE* = 0.18). In terms of Mean Squared Error, people’s pretense preferences are also better predicted by shape similarity than color similarity.

Of course, shape and color are just two salient dimensions. Many other features exist. At this point, one might reasonably object that people’s pretense preferences would be better understood as a natural generalization of the similarity structure inherent to a general-purpose representational space that encodes abundant features and captures complex statistical patterns in visual-linguistic experience. Rather than test exhaustively for each visual feature separately (a task that would be intractable practically, and difficult conceptually, as many visual features useful for recognition may not be easily separated), we examined a whole host of visual features in combination, by using machine learning models. Specifically, here we focused on vision-and-language representations derived from CLIP (Radford et al., 2021), a popular and performant multi-modal foundation model trained with a contrastive learning objective (results of two other models, ALIGN and FLAVA, were qualitatively similar to that of CLIP and reported in Supplemental Information). We derived an embedding similarity measure by considering the distance between the embedding of the image of the real-world object shown to people, and the embeddings of the words that were given to people as the proposed pretense objects. We then examined the correlation between people’s pretense preference, and the embedding-based similarity metric. We found a significant correlation (Spearman *ρ* = 0.372, *p* = 0.008, *MSE* = 0.111) between people’s pretense preference and the emebdding similarity, though this correlation was significantly lower than the correlation between people’s pretense preference and shape (Dunn and Clark’s *z* = −2.402, *p* = 0.016, computed via the cocor package in R; Diedenhofen & Musch, 2015). We did not find significant correlations between human pretense preferences and representational similarities derived from ALIGN (*ρ* = 0.138, *p* = 0.340) and FLAVA (*ρ* = 0.250, *p* = 0.079), two other instances of contemporary language-vision foundation models. This finding shows several things of importance to cognitive science and machine learning: on the cognitive side, surface-level features likely do play a role in human visual pretense, but not as large as the role of physical features (the single physical feature of shape heavily outperformed the entire combination of visual features captured by CLIP). On the AI and machine learning side, our results suggest that a general-purpose embedding space as developed in many state-of-the-art generative multi-modal foundation models, despite their fruitful success in accounting for people’s visual object representation (Conwell et al., 2024; Du et al., 2025), falls short as potential models of human-like visual pretense and imagination.

**Figure 3.**
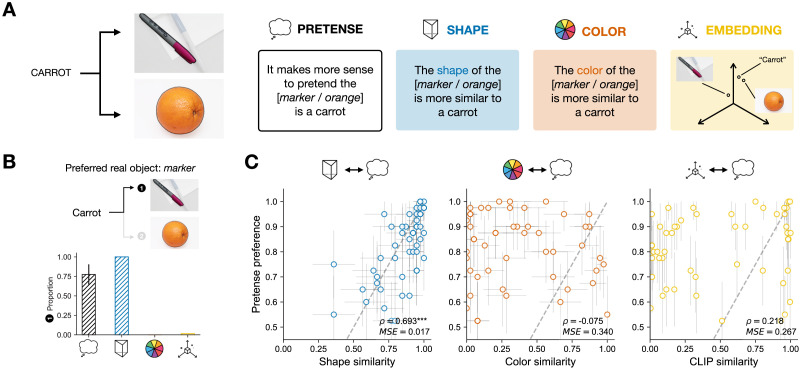
(A) Overview of Study 2, different groups of participants were resented with 50 words, and made a paired forced-choice between two image pairs according to 3 different measures (pretense, shape, color). We again considered a similarity measure based on distances in a high-dimensional multi-modal space using CLIP (Radford et al., 2021). (B) Example from one item, across the different measures. (C) Correlation between pretense preferences and participant-based similarity measures of shape, color, as well as representational similarity derived from CLIP (results of other models are included in the Supplemental Information). The dependent measures (choice proportion) for the two given options of each item are inherently center-symmetrical, and see the Supplemental Materials for plots showing the dis-preferred options. Spearman correlation (*ρ*) across items is based on the strength of preference for the preferred pretend options. Error bars indicate 95% confidence intervals with normal approximation.

In Study 2 (see Figure 3), we flipped the direction of real and pretense objects. This is similar to a case in which you start out wanting to pretend some object is a car—say, to communicate a scene—and are looking around for a suitable real world object. Other than flipping the direction of pretense, the study reused the measures, procedures, and analyses of Study 1. As Figure 3C shows, we again found that shape similarity preferences strongly correlated with pretense preferences (Spearman *ρ* = 0.693, *p* = 2.42 · 10^−8^, *MSE* = 0.017), though we note in this study the preference is a choice between alternative real objects. Color similarity was again not correlated with pretense preferences (Spearman *ρ* = −0.075, *p* = 0.607, *MSE* = 0.339). Representational similarity derived from CLIP (Radford et al., 2021) also did not predict human pretense preferences (Spearman *ρ* = 0.218, *p* = 0.128, *MSE* = 0.267). While there is a significant correlation between human pretense preferences and representational similarities derived from ALIGN (*ρ* = 0.339, *p* = 0.015), we did not find significant correlation with human preferences for FLAVA (*ρ* = 0.223, *p* = 0.119). Looking across the three pre-trained foundation models tested here, we did not see a strong and robust correlation between the preference structure in the human visual pretense and the representational similarity extracted from the statistics of large-scale text-image pairs. In short, the findings of Study 2 largely repeated the findings of Study 1, suggesting physical features such as shape play a primary role in guiding visual pretense.

Studies 1 and 2 both used a paired forced-choice design (whether pairs of pretend objects, or pairs real-world objects). While this allows for a controlled quantitative analysis, it leaves open the possibility that the experimenter-derived items are not the ones that participants would intuitively come up with on their own. To go back to the original example: while a majority of people may agree it makes more sense to pretend the block is a car rather than a strawberry, perhaps most people would not themselves come up with either ‘strawberry’ or ‘car’ when engaging in visual pretense with the block. So, To better establish the generalization of our results, in Study 3 we asked participants to come up with possible pretend options for everyday objects themselves, using a free-form response design. This was the equivalent of showing people the red block, and prompting ‘What could we pretend this to be? You can say anything’. The objects in Study 3 were the same as those used in Study 1.

**Figure 4.**
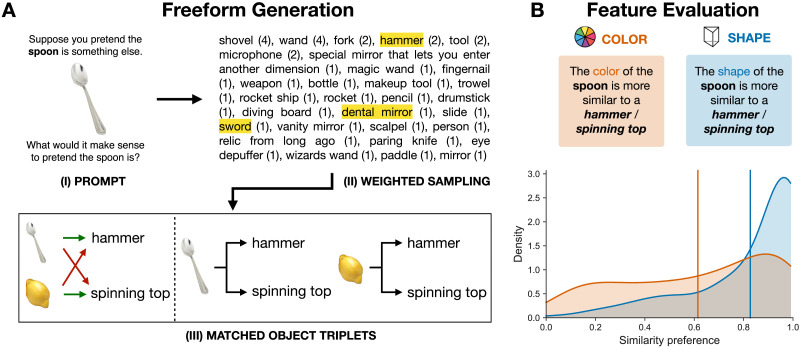
(A) Experimental procedure of Study 3, showing how one of the 150 triplets was constructed. (B) Distributions of the proportion of choosing the free-form generated pretend object relative to the randomly paired baseline, for color and shape similarity. Each distribution is based on 150 triplets. The mass of the shape-distribution being toward ‘1.0’, and higher than the color-distribution shows that people’s free-form generated pretense objects strongly resembled the original object in shape, much more so than they did the color.

People gave a variety of free-form responses to a prompt asking them to pretend an object was something else. As Figure 4A illustrates, the space of possible options that people come up with is rich and most of the elicited options occurred only once. To test the features of these responses, we randomly sub-sampled pretense objects for each real-world entity, in proportion to the relative frequency of the free-form response. Using this procedure, we created 150 triads that match each real-world prompt object with two free-form pretend responses, one taken from participants’ responses to the original object, and one taken from the responses to a different object. For example, we created the triads [spoon | hammer, spinning-top], and [lemon | hammer, spinning-top], randomly sampling ‘hammer’ from the free-form response to ‘spoon’, and ‘spinning-top’ from the free-form response to ‘lemon’ (see Figure 4A).

Using the real-to-pretend triads, we next compared the relative shape and color similarity judgments of the pretend objects to the real item, using separate groups of participants for shape and color. As shown in Figure 4B, when aggregating across the 150 triads, we found that the shape similarity preference (mean = 0.827, median = 0.95, 95% *CI* = [0.809, 0.845]) is more skewed towards the free-form generated pretend object, compared to color similarity preference (mean = 0.614, median = 0.70, 95% CI = [0.589, 0.639], Mann-Whitney *U* test, *U*_1_ = 16334.50, *p* = 1.149 · 10^−11^). In other words: even when giving free-form responses, the shape of the pretend objects that people come up with matches the shape of the real objects, over and above a surface feature like color. This result corroborates the findings of Studies 1 and 2, suggesting that spatial and physical properties of objects guide visual pretense.

Studies 1–3 support the idea that people have structured preferences in visual pretense, and that these preferences are primarily based on spatial/physical features, as measured by shape. However, these studies leave open many of the details of the process of visual pretense. Among these is the basic question of spatial alignment between the real and imagined object. Regardless of the specific features that matter for preference, it is *a priori* possible that visual pretense is simply a process of replacement: the real object is removed from one’s representation of a scene, and the pretense object put in its place. However, it seems more plausible that people systematically align the real and pretense objects, including their subparts. To go back to the opening example: when pretending the block is a car, it is theoretically possible that people would place the long axis of the car to align with the short axis of the block. But it seems more likely that people would choose to align the long axis of the car with the long axis of the block. Such a spatial alignment of parts and subparts would be a cause for the importance of shape similarity in the first place.

**Figure 5.**
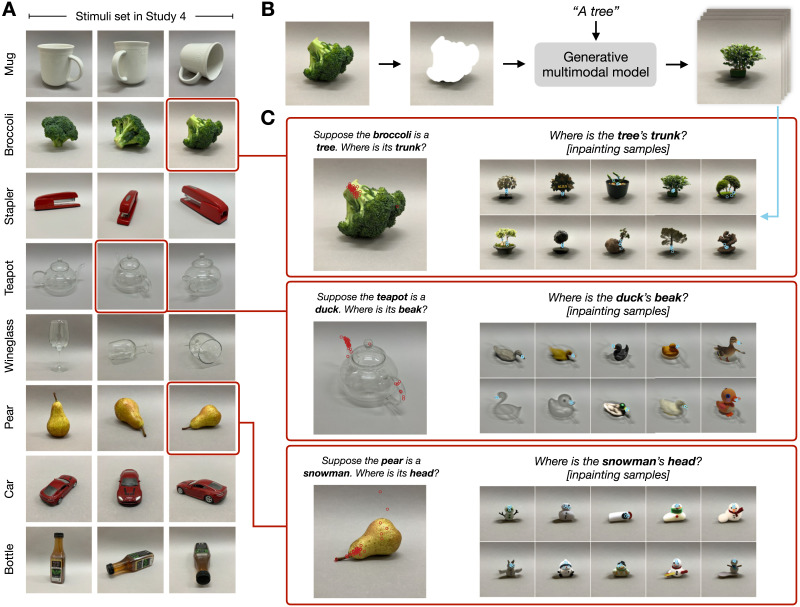
(A) The stimuli used in Study 4, 8 objects from 3 different angles. Each object was paired with a pretense entity, for example, people were asked to imagine a broccoli was a tree. (B) Inpainting example: Two generative multi-modal AI models filled in the masked area of images (e.g., a masked broccoli), given a text prompt that corresponded to the pretend object (e.g., ‘a tree’). (C) Example results of the pretense and coding tasks. Following the pretense prompt, participants were asked to locate a subpart of the pretend object (e.g., the tree’s trunk) by clicking on the image of the real object (broccoli). The subpart of the pretend object in the model-inpainted images were annotated by a separate group of participants (see Methods for details).

The goal of Study 4 was to empirically study whether and how people align the subparts of real and pretend objects. To capture the alternative of simple replacement, we used two controls: one in which people generated pretend objects without the corresponding real objects, and another in which we used current multi-modal generative models. The overall procedure is shown in Figure 5. In the main part of the study, participants were shown novel images of everyday objects (created by the experimenters) in various rotations. Participants were asked to pretend the object was something else (e.g., to pretend that a broccoli is a tree). Following the pretense prompt, participants were asked to indicate the location of different subparts of the pretend object (e.g., the trunk or crown of the tree) by clicking on the real object (broccoli). We found that participant responses were generally in agreement with one another, differed by subpart, were centered around one or a few plausible locations for subpart alignment, and tracked the rotation of the objects even in unusual orientations, as shown in Figure 5C (see Supplemental Materials for other examples). Unrelated to the specific question of subpart alignment, we note that people’s responses were clustered around the center of the mass of the relative subpart of an entity, corroborating the results of previous studies examining people’s reasoning about the location of objects in direct perception (Boger & Ullman, 2023).

**Figure 6.**
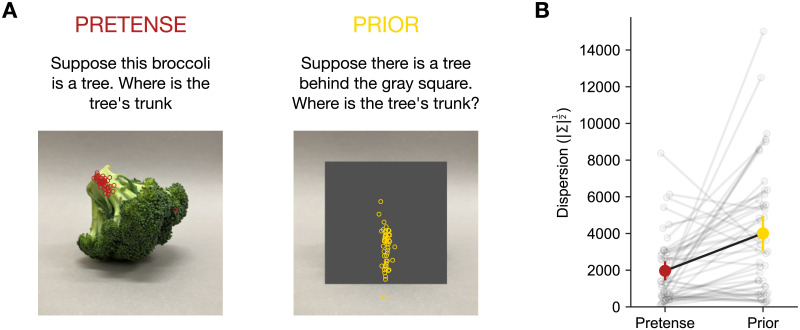
(A) Example trial of the Pretense condition vs. Prior condition. (B) Comparing the dispersion of participant responses for all 24 items across the Pretense and Prior conditions. Individual semi-transparent hollow circles are specific items. Error bars show 95% confidence intervals of the mean with normal approximation.

As a basic quantitative check, we compared the degree of dispersion among the click responses given a specific [image, prompt] pair across Pretense and Prior conditions. In the Pretense condition, participants saw the original image of the real object, while in the Prior condition participants were given a processed image in which a gray square fully covers the real object (see Figure 6). The Prior condition was a necessary control, as otherwise it may be objected that when people generate a pretense object in the location of the real object, the orientation of the pretense object obeys prior biases (for example, when asked to imagine a car in their mind’s eye, people are unlikely to imagine it upside down), and that these biases just so happen to line up with the real object. The dispersion of people’s response clicks was quantified as ${\left|\Sigma \right|}^{\frac{1}{2}}$, the square root of the determinant of the co-variance matrix of participant clicks (Wilks, 1932). As shown in Figure 6, participant responses for the location of specific sub-parts of the imagined objects in the Pretense condition had a significantly lower dispersion than in the Prior condition (*t*(47) = −4.077, *p* = 0.00017). This suggests that the way people mentally construct the pretend object is sensitive to, and constrained by the perceptual input of the real object.

**Figure 7.**
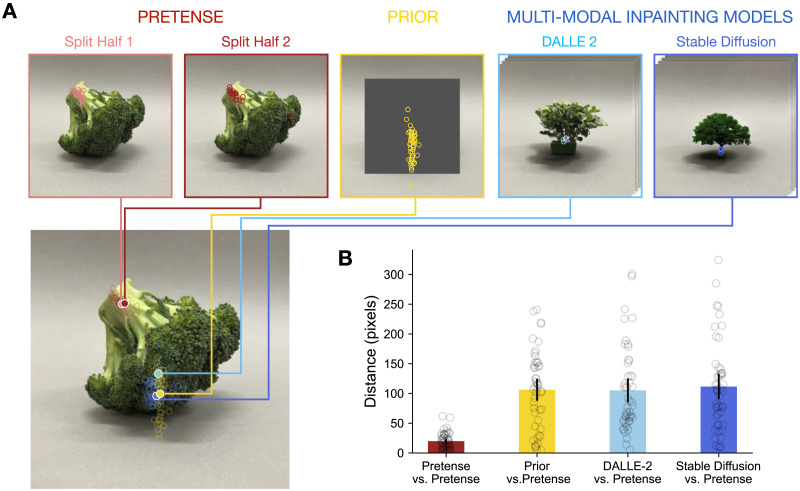
Comparing the location of imagined sub-parts (A) A specific example (broccoli) illustrates the location of an imagined sub-part (tree trunk), for random half splits of the Pretense condition, the Prior condition, and aggregations of 10 inpainting samples from DALLE-2 and Stable Diffusion. (B) The distances between the center of pretend sub-part location, comparing the Pretense condition to the other outputs for all 24 items. The lower distance for Pretense-Pretense compared to the other conditions indicates people are consistent in locating the sub-part (e.g., tree-trunk) in pretense, and that its location is not explained by a simple process of substitution. Individual circles show specific items. Error bars show 95% confidence intervals with normal approximation.

In addition to a dispersion analysis, we compared the distances between the spatial location of imagined sub-parts in the Pretense and Prior conditions (Figure 7). We randomly split the data in the Pretense condition into two halves, and then computed the distance between the center of clicks in the first randomly-split half to the center of clicks in the second randomly-split half, averaged across all the items. This gives us an estimation of the intrinsic variability under the Pretense condition. We then computed the distance between the center of clicks in the first randomly-split half in the Pretense condition to the center of clicks in the Prior condition, averaged across all items. As shown in Figure 7, the center of the locations that participant click on the subpart are significantly different across the Pretense and the Prior condition.

As illustrated in Figure 5, the inpainting samples of contemporary multi-modal AI models do not show sensitivity to the orientation of the real object in the way humans do: people systematically track the stem of the broccoli as the trunk of the imagined tree through its rotations, while the models consistently inpaint a tree in a canonical position in the location left behind by the absence of the broccoli. To quantitatively measure the differences between the models’ output and people’s imagination, we recruited an independent group of human annotators to identify the location of the relevant subparts in each of the model-inpainted samples. We aggregated the annotator clicks for each item, and measured the distance between the locations of imagined subparts in the Pretense condition and that of the inpainted model output, using the same metric and procedure as the comparison analysis of the Pretense and Prior conditions. As shown in Figure 7 the inpainted images generated by state-of-art multi-modal models are significantly different from people’s imagination in terms of how the subparts of a pretend object are mapped onto the real object.

## DISCUSSION

A child waves a stick, yelling: “behold my wand!”. A teacher holds up an apple and says “this is the sun”. A parent points out two small carrots next to a big carrot and nudges his kids: “that’s us”. These acts are a specific kind of pretense, in which an object in the world is taken to stand in for an object in the mind. Such acts are common, intuitive, and useful. Here, we examined whether people have systematic preferences in the kind of visual pretense they come up with or accept as sensible, and what these preferences are guided by. We proposed that there exists a hierarchy of features that constrain the way people entertain the relationship between a real object and a pretend object. We suggested that within this hierarchy, physical and spatial features will matter more than surface features, and that people’s visual pretense is primarily guided by a structural, spatial alignment between real and pretend objects.

We focused on shape and color as two salient dimensions representing spatial/physical features and surface features more generally, but we also used current cutting-edge vision models to capture a host of visual features encoded in a general-purpose visual-semantic embedding space. Across four studies, we found that while pretense is in theory a departure from reality, in practice people have systematic preferences in their visual pretense. The single spatial property of shape similarity strongly correlated with people’s pretense across a variety of tasks, including both constrained and free-form responses, above and beyond both human-based judgments of color similarity and machine models that capture a host of visual features.

We stress that while we found that shape plays a strong role in base visual pretense, we do not mean this to rule out the role of surface-level features. While Study 2 did not find a correlation between color or other surface-level features and pretense preferences, Studies 1 and 3 did find an above-chance association between them. Also, one does not need a host of studies to know that people will choose a red block over an otherwise identical blue block when pretending something is a strawberry. Surely, surface-level features matter to some degree. We also note that the exact relative importance of different features may depend on the specific context. Our studies did not give people a context for *why* they are asked to pretend an object is another object. This was an intentional choice, as we were interested in the base-level preference, allowing people to come up with whatever context they wished. We maintain that our results suggest that shape (and more generally, physical and spatial properties) will matter across a wide variety of contexts, but more studies are necessary to show how context can vary the relative importance of features for visual pretense.

Related to the question of whether and how context matters, there is the question of *why* spatial features seem to guide visual pretense, and whether this is a ground-floor preference, or something rooted in more fundamental cognitive principles. One possibility is that the importance of shape is based on the functional use of objects, which in turn is constrained by shape. However, we note that while in some cases shape can explain function, many of the items we used were not artifacts, and do not have a clearly defined function. A different possibility is that pretense is driven by psychological essentialism (Gelman, 2003, 2004), and pays more attention to features that are strong cues of the object category. So, in the case of visual pretense, shape might be preferred over color for being less likely to be an accidental feature of most everyday objects. While the empirical data from Studies 1–3 is consistent with this view, the essentialist view might underestimate the pragmatic nature of visual pretense. It also does not on its own explain the importance of mapping subparts and orientation, as seen in Study 4 (the orientation of a pretend car is not an essential feature of it being a car, but it is important when lining it up with a real block). Another option is that visual pretense is about the repurposing of real-world objects to represent ideas in the mind, rather than an inference over properties pointing to the deep essences of things. On this view, what matters is not the essence of categories, but the potential use of an object as an iconic sign to achieve a communicative goal (Clark, 2016; Fan et al., 2023; Peirce, 1894), and that for this spatial and physical features matter more. Of course, other possibilities may exist, and further work is needed to examine these theoretical ideas.

In our studies, we used current models of visual processing as benchmarks in several ways. In all cases, we found a gap between state-of-the-art machine models and people’s behavior. This gap existed when comparing people’s pretense preferences (Studies 1–2), and when examining subpart alignment (Study 4). To the degree that people rely on spatial and physical features in visual pretense, this gap is not surprising. Vision models based on convolutional neural networks have a notoriously hard time picking up the shape bias (Geirhos et al., 2019; Hermann et al., 2020; Ritter et al., 2017). Pre-trained multi-modal models show limited capacity for spatial reasoning (Wang et al., 2025) and abstract visual reasoning of whole-part relationship (Ji et al., 2022). Targeted evaluations of CLIP and vision language models show that they display a preference to shape over texture on a cue conflict dataset (Geirhos et al., 2019) previously used to assess the shape bias of convolutional neural networks-based vision models. However, the shape bias of CLIP is still relatively weak compared to humans (Gavrikov et al., 2025). Although the performances of the multi-modal foundation models in our Studies 1–2 do not necessarily imply that CLIP and other multi-modal foundation models only learn surface-level features from large-scale text-image data, these results are consistent with recent findings that suggest a lack of robust representation of shape in CLIP as well as the visual encoders of major multi-modal language models (Eppel et al., 2025; Rudman et al., 2025). And, while recent work suggests that generative vision models might have acquired stronger human-like shape bias in their visual representation (Jaini et al., 2023), we found that multi-modal generative models (DALLE-2, Ramesh et al., 2022; Stable Diffusion; Rombach et al., 2022) that are in principle able to ‘fill in’ novel objects by inpainting, did so in a way that did not match people’s behavior. This is hardly surprising, the models were given as input ‘hollowed out’ versions of the real-world object, without the subpart information to go on for filling them in. But that is precisely the point: people were not explicitly given this subpart information, and yet presumably they extracted it, and took it into account. To the degree that machine vision models can be used to capture visual pretense, they too will need to extract or be given this subpart, spatial information. Notably, recent work in machine learning demonstrates the potential of integrating a structure-mapping engine with deep neural networks (Webb et al., 2023). Such a model can explicitly align subparts between objects in two images or 3D representations. Building on this idea, future work can investigate a potentially promising proposal for a cognitively-inspired model of human visual pretense, which can account for the systematic alignment of subparts.

We recognize some limitations of this work as well as opportunities for advancing the methodology. First, the representations derived from the AI models are high-dimensional and distributed. It is challenging to delineate exactly what and how features are encoded. A refined characterization of the models’ embedding space could inform the nature of the observed gap between people and current models. Relatedly, one may consider developing new tools that can extract or erase specific feature subspace from a model’s learned general embedding space (e.g., Belrose et al., 2023; Ravfogel et al., 2020). Second, our studies used natural image stimuli that were originally curated for computer vision tasks, such as object segmentation and scene understanding. While we conducted feature norming studies to measure shape and color similarities, it could be challenging to precisely match the overall distributions of feature similarity in the stimuli. One potential approach to more directly controlling the objects’ visual appearance in the stimuli is to use rendered images from computer graphics simulation. For example, instead of sifting through a gigantic pile of images taken in everyday scenes, one could use a graphics engine like Blender to synthesize objects in a specific size, orientation, color, and texture, which could support systematic manipulation of spatial/physical or surface-level features.

Visual pretense relates to analogy and depiction. Pretense at its core is supposition: taking some aspects of a real object and mentally setting it to an alternative value. But as our studies suggest, reasonable suppositions in theory are not always equally sensible to people. Stronger or weaker shape resemblance between the real and pretend objects correlates with people’s pretense preferences, and the specifics of the real object’s spatial layout guides the construction of the specific imagery of the pretend object. Just like depiction^1^, visual pretense turns a real-world object into an iconic representation of some referent. The resemblance between the real and the imagined naturally enables inferences about how the subpart structure of the pretend object would map onto that of the real object, similar to analogy (Gentner, 1983; Gentner & Markman, 1997; Rabkina & Forbus, 2022). But we conjecture that appraising the relationship between a real object and the pretend object it stands for involves cognitive processes beyond analogical reasoning. To illustrate, consider this item from Study 1 (marker → plane | lantern). Our behavioral data showed that a majority of the participants (72.5%, 29/40) preferred to pretend the marker (same as the one in Figure 3) to be a plane. Here is a puzzle for the canonical theory of analogy: we seem to intuitively see the marker as the body of the pretend plane, while the plane’s wings—highly relevant to how a plane typically looks and works—do not correspond to any subpart of the actual marker. Rather, the wings seem painted *ad hoc* onto the mental image. This example suggests that apprehending visual pretense potentially involves manipulating imagery representations. One might stretch, carve, weld, or repaint things on a mental canvas to apprehend the resemblance between the real and pretend objects, with certain visual details of the real object retained in the pretense and others discarded. Our analysis of the distinction between visual pretense and analogy shares a similar stance with a recent critique of the analogical account of children’s pretend play (Revencu, 2024). Still, we need further theoretical, empirical, and computational work to develop the full argument from the anecdotal “marker → plane” example.

Pretense is an act of the imagination, and our work relates to a broader line of work examining the important role of physical and spatial properties in imagery and imagination. For example, McCoy and Ullman (2019) found that magical spells involving a physical transformation of an object’s shape is judged as more effortful than a surface change of color. The physical and spatial properties of imagined entities (such as their size or relative location) also seem to be more likely to be committed to a mental scene than surface properties (such as color or texture; Bigelow et al., 2023). Despite the differences in the modes of imagination being investigated in prior literature and the current studies here, the findings across these works suggest a converging idea that the spatial and physical structure of objects guide much of people’s imagination.

Pretending that things are something they are not is a common yet distinctive part of how we play, communicate, teach, and create (Mollerup, 2019). Our studies here are steps on the path to describe and explain the systematic hierarchy of features that guides this pretense. The studies combined behavioral experiments and computational models, and their results suggest a primary role for spatial and physical properties over surface properties in visual pretense. The results also point to critical gaps in current machine models of vision that do not take physical and spatial properties into account. We hope this works creates new directions for studying playful human expressions, and helps explain the magic that happens when a child picks up a stick and calls it a wand.

## ACKNOWLEDGMENTS

We thank members of the CoCoDev labs at Harvard, members of the ECCL Lab at MIT, as well as the participants in the Imagistic Cognition Workshop in Salzburg and the 49th Annual Meeting of Society for Philosophy and Psychology for their insightful comments.

## FUNDING INFORMATION

This work is supported by the Hodgson Fund, a Harvard University Department of Psychology Research Innovation Fund (PQ), the Center for Brains, Minds, and Machines (TDU), and the Jacobs Family Foundation (TDU).

## AUTHOR CONTRIBUTIONS

P.Q.: Conceptualization; Funding acquisition; Investigation; Methodology; Visualization; Writing – Original draft; Writing – Review & editing. T.D.U.: Conceptualization; Funding acquisition; Methodology; Writing – Original draft; Writing – Review & editing.

## DATA AVAILABILITY STATEMENT

Materials, data, and code are available in the following Open Science Framework repository: https://osf.io/ykdr5.

## Note

^1^ Resemblance has been long argued as a central property of depiction, or *pictorial representation*. Resemblance theories of depiction can be traced back to as early as Plato’s *Republic* and continue to carry significance in contemporary thoughts (Abell, 2009; Blumson, 2014; Clark, 2016), although philosophical debates remain on whether (and what notion of) resemblance is essential to explaining how pictures are understood (Abell & Bantinaki, 2010; Goodman, 1976; Greenberg, 2013; Lopes, 1996).

## Supplementary Material


