# Supplementary material for: Shape Guides Visual Pretense
Source: Open Mind (Camb). 2025 Dec 18;9:2092–113. doi: 10.1162/OPMI.a.277 (PMC12768553; doi:10.1162/OPMI.a.277)
Supplement: Supplementary file 1 [file opmi-09-2092-s001.pdf]

Supplemental Information for
Shape Guides Visual Pretense

Contents

A Additional Analyses **2**

B Supplemental Information for Study 1 **3**

C Supplemental Information for Study 2 **22**

D Supplemental Information for Study 3 **49**

E Supplemental Information for Study 4 **67**

A Additional Analyses

Study 1. We conducted a mixed-effect logistic regression analysis on participants choices in the pretense, shape judgment, and color judgment tasks. For the analysis of the feature judgment data, we coded people's response to a trial in the SHAPE and COLOR tasks as 1 if the response was the preferred pretense option (i.e. chosen by more than half of the participants in the PRETENSE task). We found that the pretense object that was preferred in the PRETENSE task was more likely chosen as the more similar option to the real object in the shape similarity judgment task than in the color similarity judgment task ($\beta = 1.898$, $z = 4.032$, $p = 5.53 \cdot 10^{-5}$ model, formula: `is_preferred_pretense_option_chosen` $\sim$ `is_shape_judgment + (is_shape_judgment | item) + (is_shape_judgment | subject)`).

We also ran a mixed-effect logistic regression to examine the effects of shape similarity and color similarity in predicting people's choices in the pretense task. The dependent variable was people's responses in the pretense task, which were coded as 1 if the response was the preferred pretense option across participants. To use a concrete example, if the pretense task included the real object 'block' and the pretense objects 'car' and 'strawberry', and more than half of people reported that it would make more sense to pretend the 'block' was a 'strawberry', then 'car' was coded as 1 and 'strawberry' was coded as 0 for the dependent variable. The predictors were the proportions of participants choosing the preferred pretense object in the shape or color similarity judgment task for specific items. We use the following mode formula: `is_preferred_option_chosen` $\sim$ `shape + color + (1 + shape + color | item) + (1 + shape + color | subject)`. We found significant effects of both shape similarity ($\beta = 3.099$, $z = 4.716$, $p = 2.41 \cdot 10^{-6}$) and color similarity judgments ($\beta = 0.848$, $z = 2.365$, $p = 0.018$) in predicting participants' choices in the pretense task, but the effect of shape was significantly stronger than that of color ($p = 0.00013$).

Study 2. We again conducted a mixed-effect logistic regression analysis on people's choices in the pretense, shape judgment, and color judgment tasks. As above, for the analysis of the feature judgment data, we coded people's response to a trial in the SHAPE and COLOR tasks as 1 if the response was the preferred pretense option (i.e. chosen by more than half of the participants in the PRETENSE task), similar to the analysis in Study 1. We again found that the pretense object that was preferred in the PRETENSE task was more likely chosen as the more similar option to the real object in the shape similarity judgment than in the color similarity judgment ($\beta = 3.231$, $z = 7.249$, $p = 4.21 \cdot 10^{-13}$, model formula: `is_preferred_pretense_option_chosen` $\sim$ `is_shape_judgment + (is_shape_judgment | item) + (is_shape_judgment | subject)`).

We ran a mixed-effect logistic regression to examine the effects of shape similarity and color similarity in predicting people's choices in the pretense task. The dependent variable is people's responses in the pretense task, which were coded as 1 if the response was the preferred pretense option across participants. The predictors are the proportions of people choosing the preferred pretense option in the shape or color similarity judgment task for specific items. We use the following mode formula: `is_preferred_option_chosen` $\sim$ `shape + color + (1 + shape + color | item) + (1 + shape + color | subject)`. We found a significant effect of shape similarity judgment ($\beta = 4.844$, $z = 6.021$, $p = 1.73 \cdot 10^{-9}$) in predicting participants' choices in the pretense task, but not for the color similarity judgment

($\beta = 0.107$, $z = 0.309$, $p = 0.757$).

Study 3 Similar to the analyses above, we conducted a mixed-effect logistic regression analysis on people's choices in the feature evaluation tasks. We coded people's response as 1 if it was the pretense option for the corresponding real object as elicited in the free-form pretense task, and 0 for the randomly paired option. We found that the pretense option elicited for the corresponding real object was more likely chosen as the more similar option in the shape similarity judgment than in the color similarity judgment task, compared to the randomly paired option ($\beta = 3.231$, $z = 7.249$, $p = 4.21 \cdot 10^{-13}$, model formula: `is_preferred_pretense_option_chosen` $\sim$ `is_shape_judgment + (is_shape_judgment | item) + (is_shape_judgment | subject)`).

B Supplemental Information for Study 1

Figure S1-S50 showcase the experimental materials used in the behavioral experiments and results from people and the multi-modal embedding space of CLIP (Radford et al., 2021). In each figure, the left panel shows the triad, the image of the real object and two pretend options. The right panel plots the proportion of participant choosing one of the two options in the PRETENSE, SHAPE, and COLOR conditions, as well as representational similarity derived from CLIP embedding space. See *Methods* section in the main text for details of the experimental procedure. All the error bars represent 95% confidence interval with normal approximation. Figure S51 plots people's pretense preferences against feature judgments in terms of shape and color, as well as representational similarity in CLIP embedding space, across all items. Figure S52 plots people's pretense preferences against representational similarity in the learned embedding space of two other representative multi-modal foundation models, ALIGN and FLAVA.

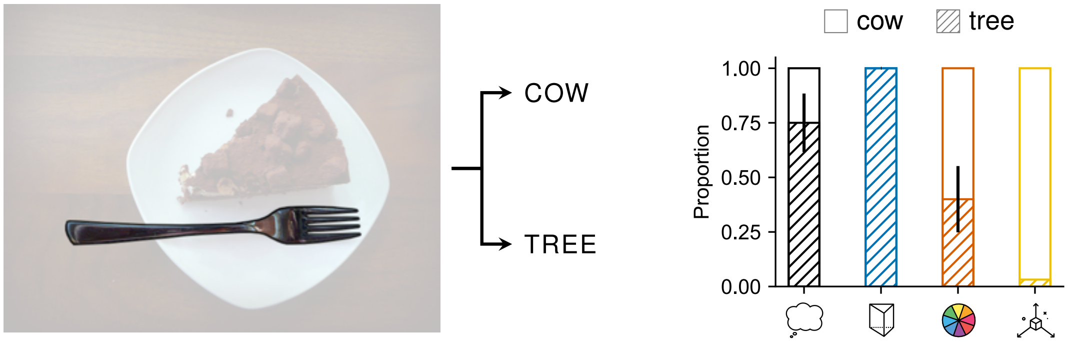

Figure S1: For the specific item (fork→[cow/tree], left panel), the proportion of participant choosing one of the two options in the Pretense, Shape, and Color conditions, as well as representational similarity derived from CLIP embedding space.

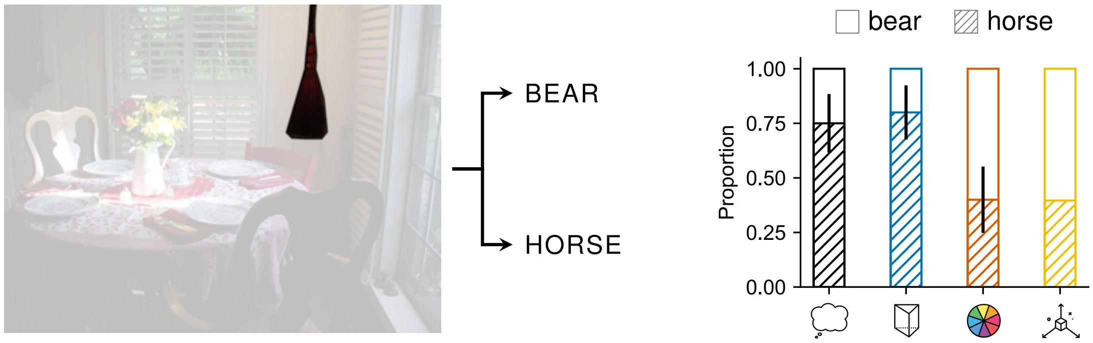

Figure S2: For the specific item (broom→[bear/horse], left panel), the proportion of participant choosing one of the two options in the Pretense, Shape, and Color conditions, as well as representational similarity derived from CLIP embedding space.

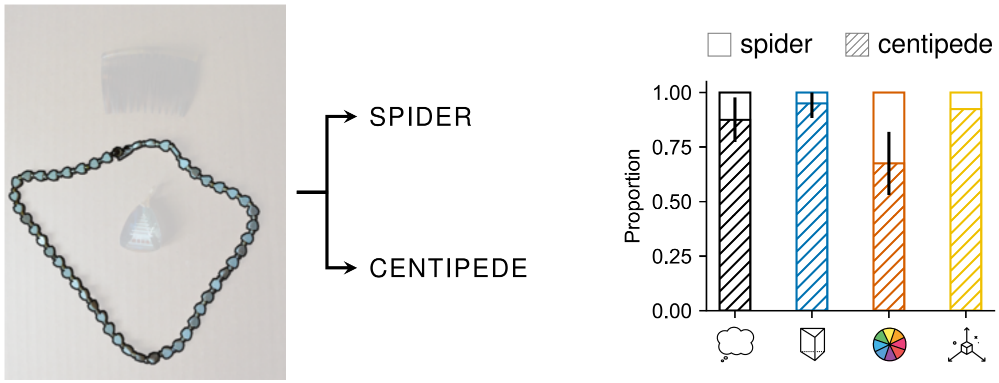

Figure S3: For the specific item (necklace→[spider/centipede], left panel), the proportion of participant choosing one of the two options in the Pretense, Shape, and Color conditions, as well as representational similarity derived from CLIP embedding space.

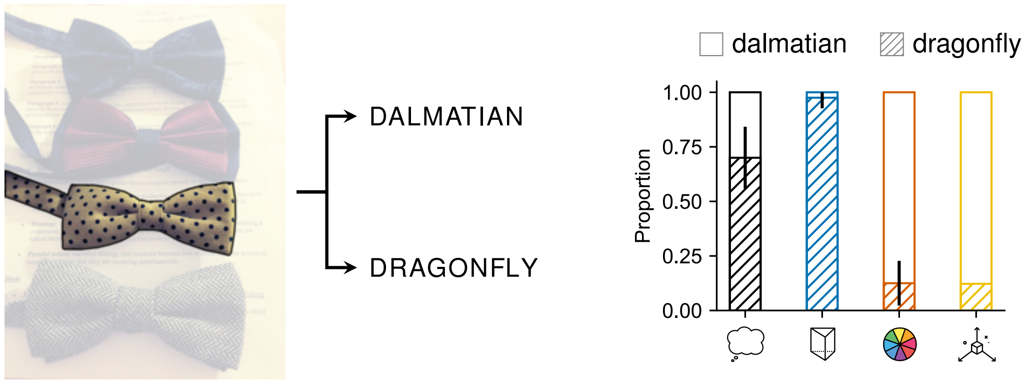

Figure S4: For the specific item (bow-tie→[dalmatian/dragonfly], left panel), the proportion of participant choosing one of the two options in the Pretense, Shape, and Color conditions, as well as representational similarity derived from CLIP embedding space.

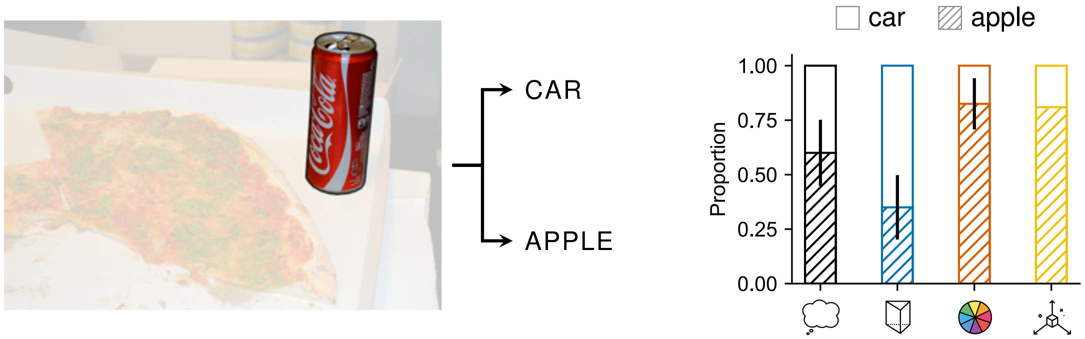

Figure S5: For the specific item (can→[car/apple], left panel), the proportion of participant choosing one of the two options in the Pretense, Shape, and Color conditions, as well as representational similarity derived from CLIP embedding space.

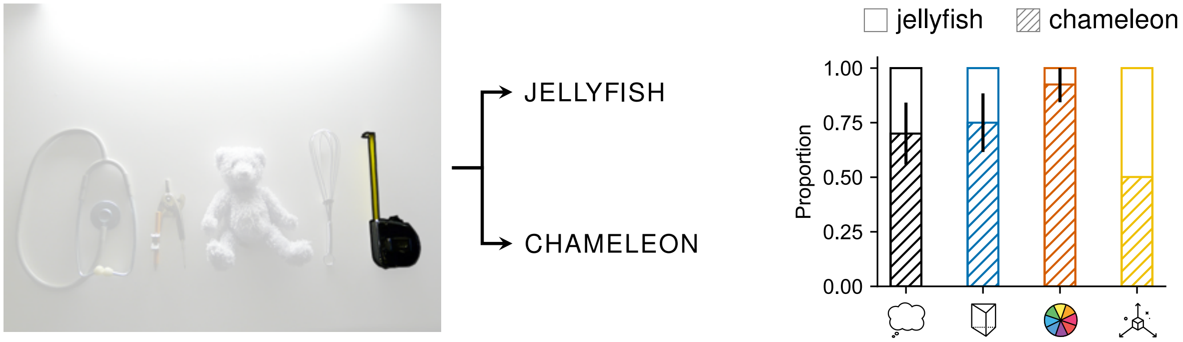

Figure S6: For the specific item (tape measure→[jellyfish/chameleon], left panel), the proportion of participant choosing one of the two options in the Pretense, Shape, and Color conditions, as well as representational similarity derived from CLIP embedding space.

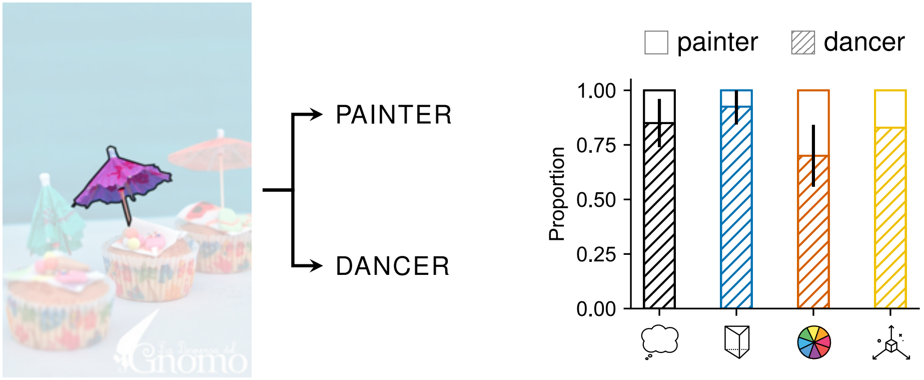

Figure S7: For the specific item (umbrella→[painter/dancer], left panel), the proportion of participant choosing one of the two options in the PRETENSE, SHAPE, and COLOR conditions, as well as representational similarity derived from CLIP embedding space.

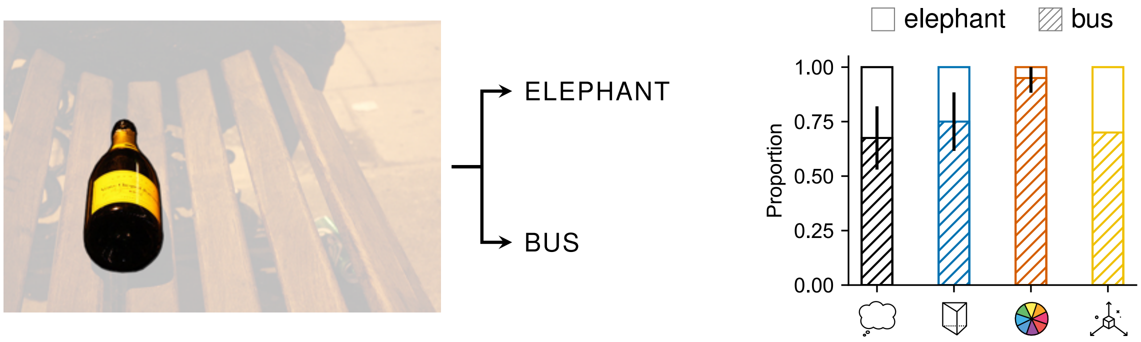

Figure S8: For the specific item (wine bottle→[elephant/bus], left panel), the proportion of participant choosing one of the two options in the PRETENSE, SHAPE, and COLOR conditions, as well as representational similarity derived from CLIP embedding space.

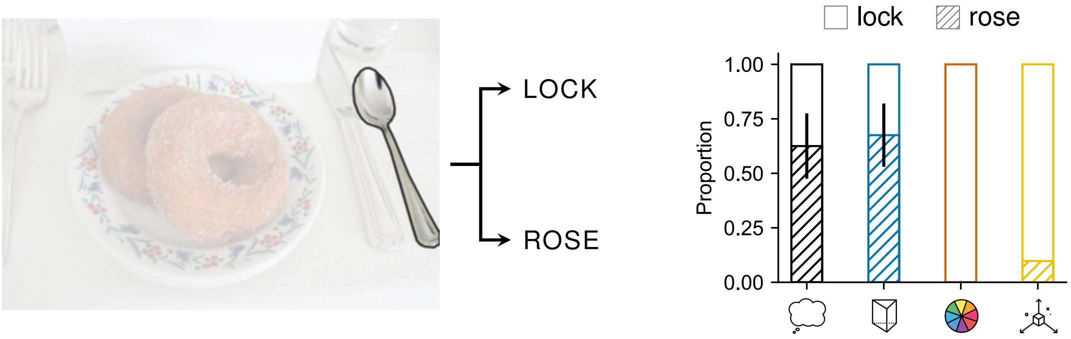

Figure S9: For the specific item (spoon→[lock/rose], left panel), the proportion of participant choosing one of the two options in the PRETENSE, SHAPE, and COLOR conditions, as well as representational similarity derived from CLIP embedding space.

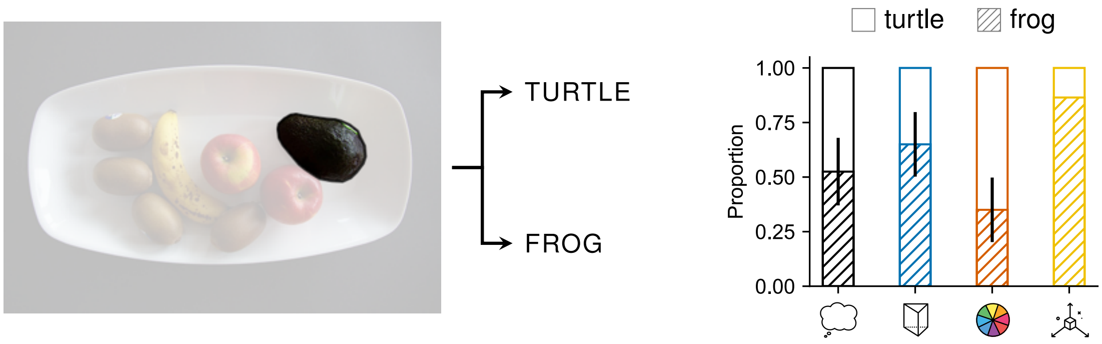

Figure S10: For the specific item (avocado→[turtle/frog], left panel), the proportion of participant choosing one of the two options in the PRETENSE, SHAPE, and COLOR conditions, as well as representational similarity derived from CLIP embedding space.

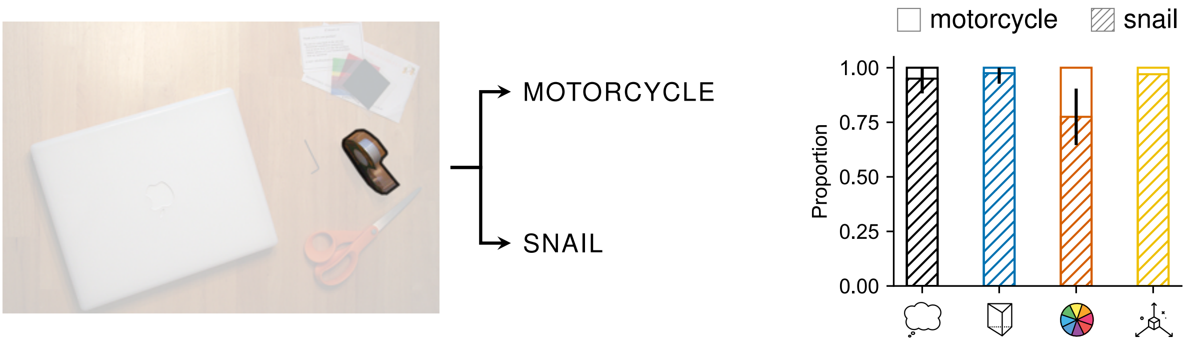

Figure S11: For the specific item (tape→[motorcycle/snail], left panel), the proportion of participant choosing one of the two options in the PRETENSE, SHAPE, and COLOR conditions, as well as representational similarity derived from CLIP embedding space.

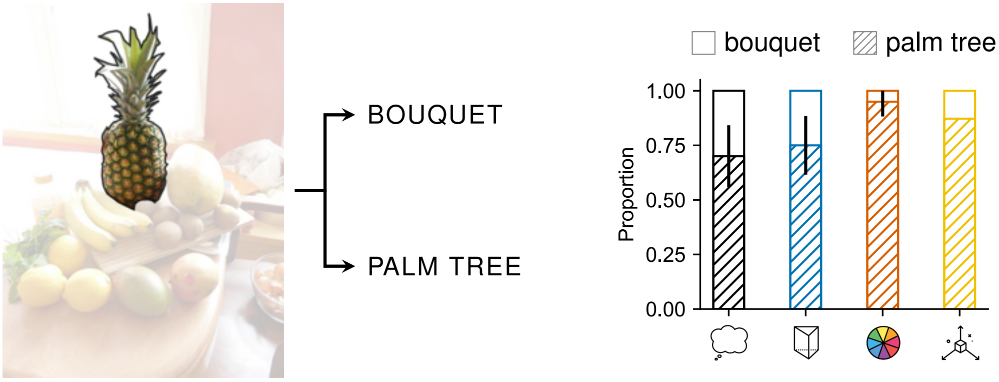

Figure S12: For the specific item (pineapple→[bouquet/palm tree], left panel), the proportion of participant choosing one of the two options in the PRETENSE, SHAPE, and COLOR conditions, as well as representational similarity derived from CLIP embedding space.

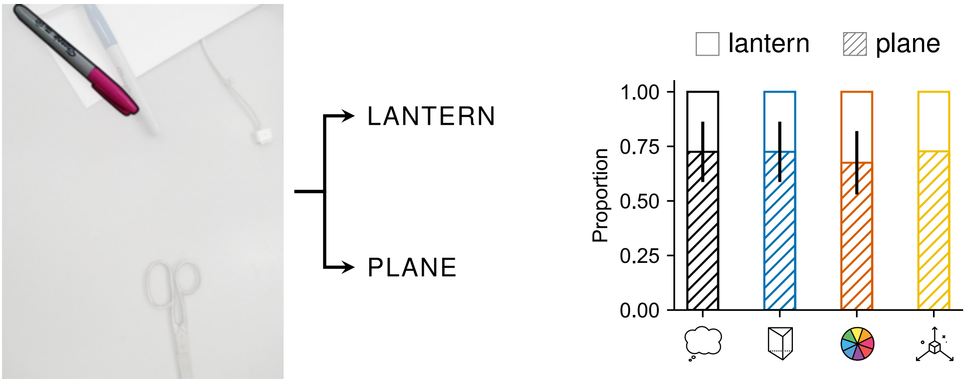

Figure S13: For the specific item (marker→[lantern/plane], left panel), the proportion of participant choosing one of the two options in the PRETENSE, SHAPE, and COLOR conditions, as well as representational similarity derived from CLIP embedding space.

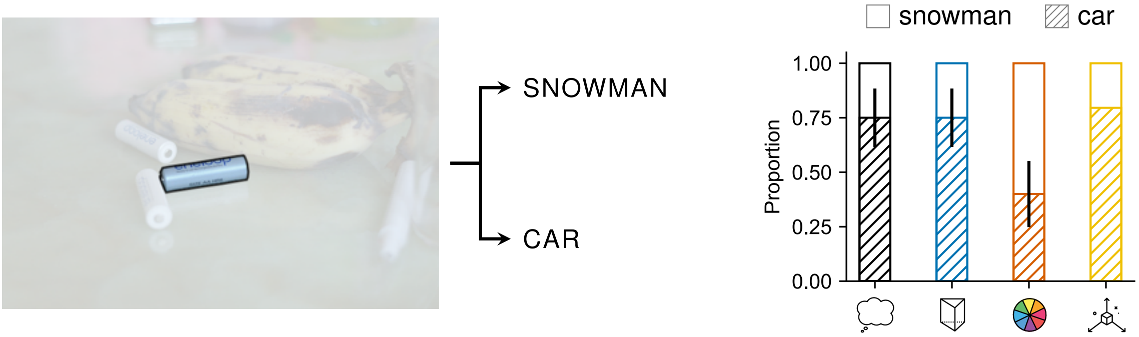

Figure S14: For the specific item (battery→[snowman/car], left panel), the proportion of participant choosing one of the two options in the PRETENSE, SHAPE, and COLOR conditions, as well as representational similarity derived from CLIP embedding space.

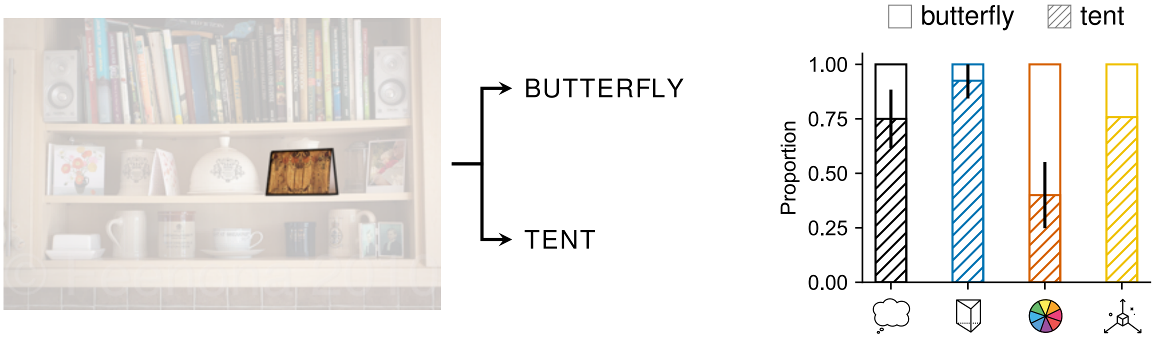

Figure S15: For the specific item (card→[butterfly/tent], left panel), the proportion of participant choosing one of the two options in the PRETENSE, SHAPE, and COLOR conditions, as well as representational similarity derived from CLIP embedding space.

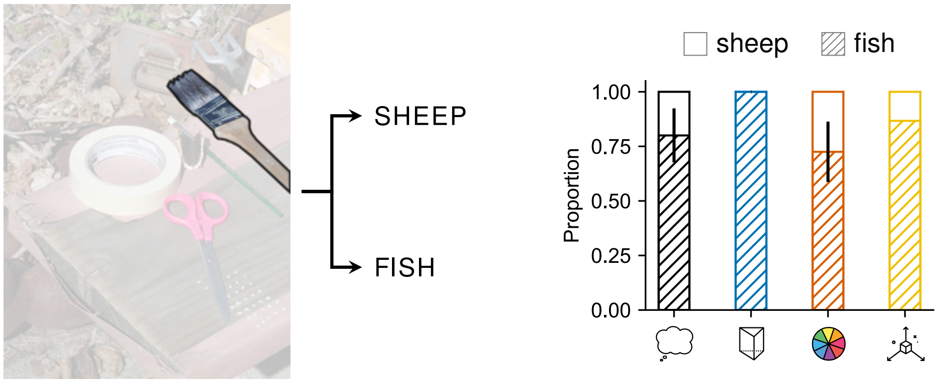

Figure S16: For the specific item (paint brush→[sheep/fish], left panel), the proportion of participant choosing one of the two options in the Pretense, Shape, and Color conditions, as well as representational similarity derived from CLIP embedding space.

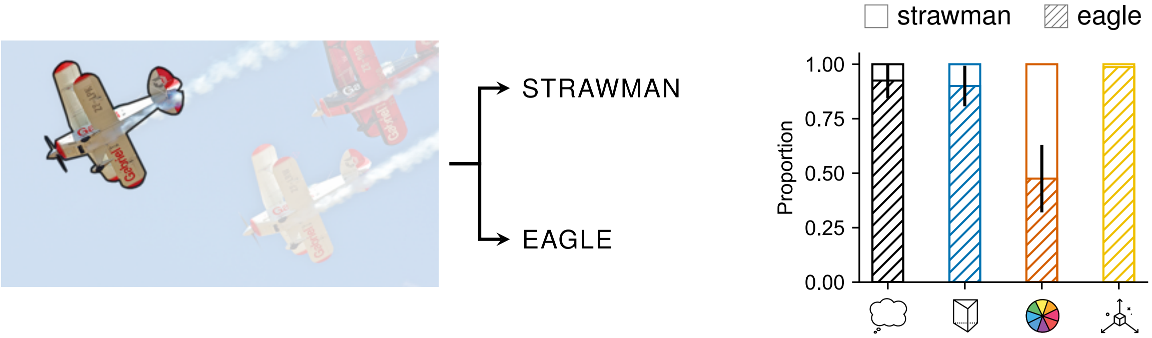

Figure S17: For the specific item (airplane→[strawman/eagle], left panel), the proportion of participant choosing one of the two options in the Pretense, Shape, and Color conditions, as well as representational similarity derived from CLIP embedding space.

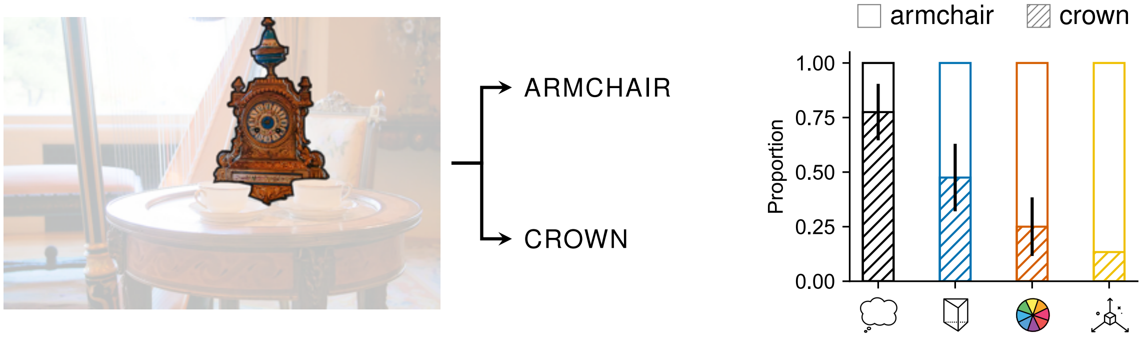

Figure S18: For the specific item (clock→[armchair/crown], left panel), the proportion of participant choosing one of the two options in the Pretense, Shape, and Color conditions, as well as representational similarity derived from CLIP embedding space.

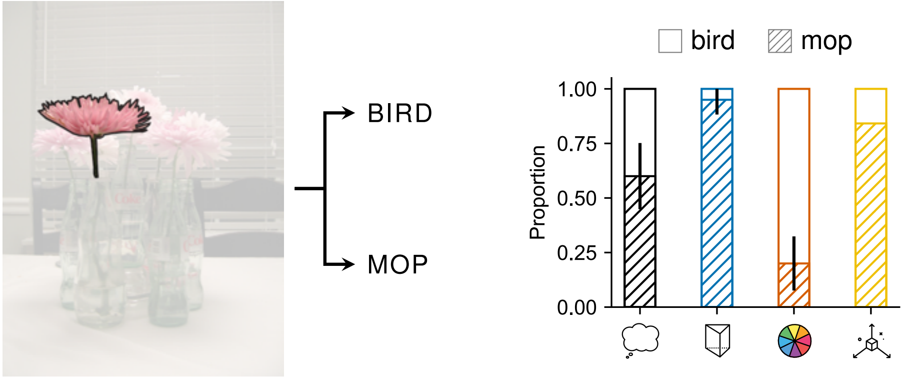

Figure S19: For the specific item (carnation→[bird/mop], left panel), the proportion of participant choosing one of the two options in the PRETENSE, SHAPE, and COLOR conditions, as well as representational similarity derived from CLIP embedding space.

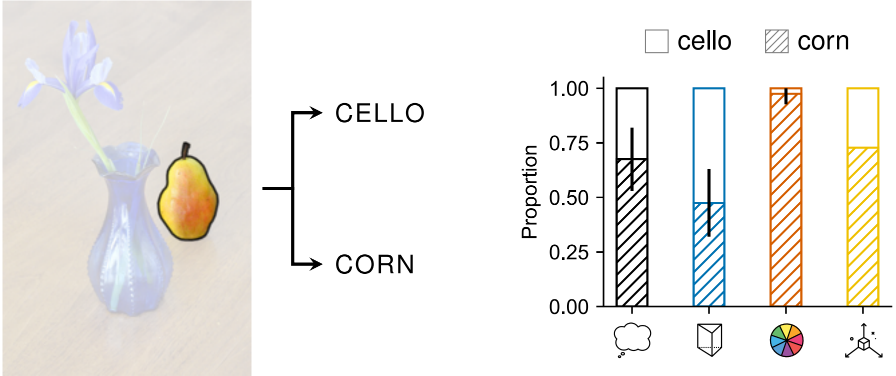

Figure S20: For the specific item (pear→[cello/corn], left panel), the proportion of participant choosing one of the two options in the PRETENSE, SHAPE, and COLOR conditions, as well as representational similarity derived from CLIP embedding space.

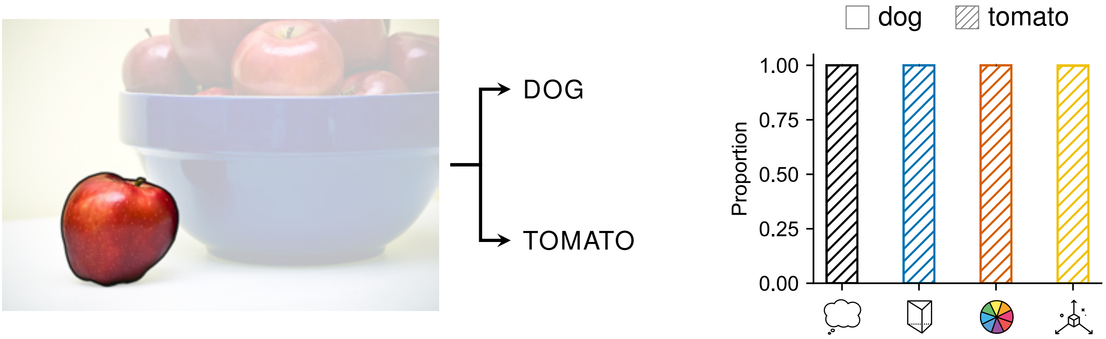

Figure S21: For the specific item (apple→[dog/tomato], left panel), the proportion of participant choosing one of the two options in the PRETENSE, SHAPE, and COLOR conditions, as well as representational similarity derived from CLIP embedding space.

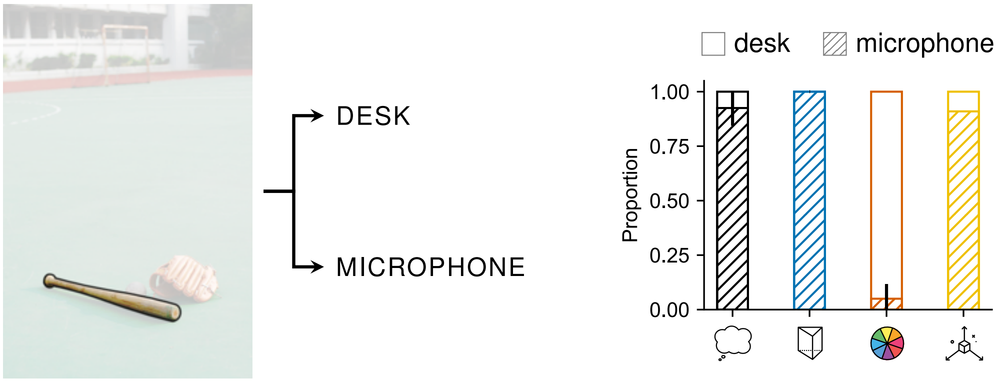

Figure S22: For the specific item (baseball bat→[desk/microphone], left panel), the proportion of participant choosing one of the two options in the Pretense, Shape, and Color conditions, as well as representational similarity derived from CLIP embedding space.

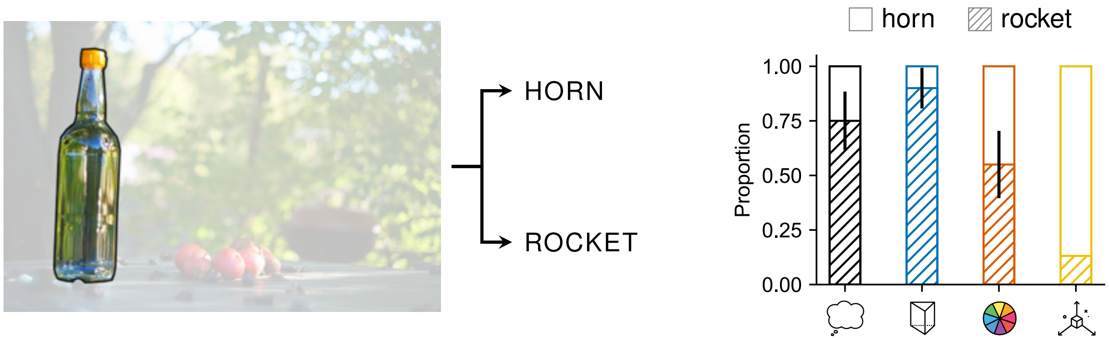

Figure S23: For the specific item (bottle→[horn/rocket], left panel), the proportion of participant choosing one of the two options in the Pretense, Shape, and Color conditions, as well as representational similarity derived from CLIP embedding space.

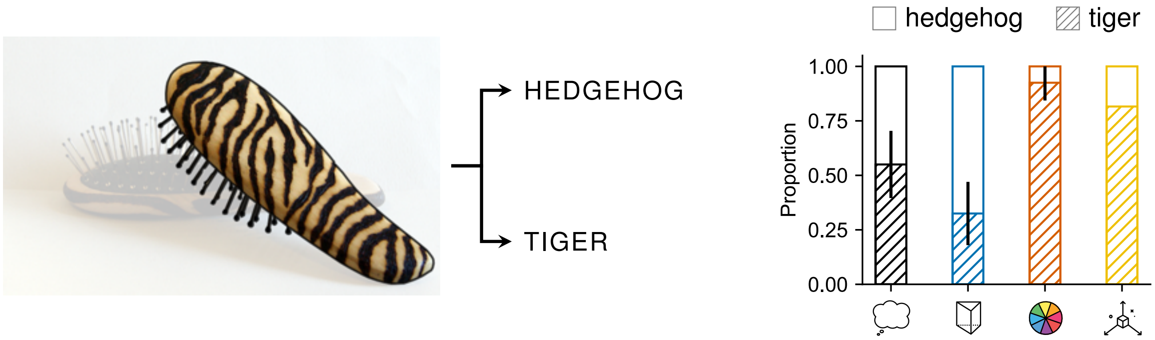

Figure S24: For the specific item (hairbrush→[hedgehog/tiger], left panel), the proportion of participant choosing one of the two options in the Pretense, Shape, and Color conditions, as well as representational similarity derived from CLIP embedding space.

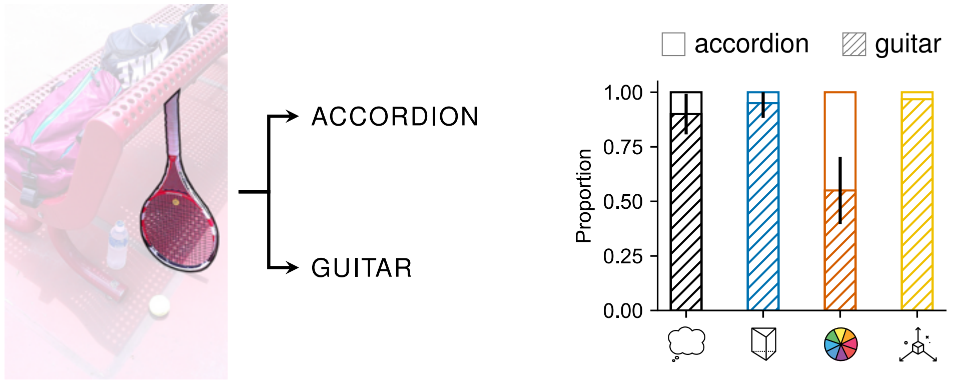

Figure S25: For the specific item (tennis racket→[accordion/guitar], left panel), the proportion of participant choosing one of the two options in the PRETENSE, SHAPE, and COLOR conditions, as well as representational similarity derived from CLIP embedding space.

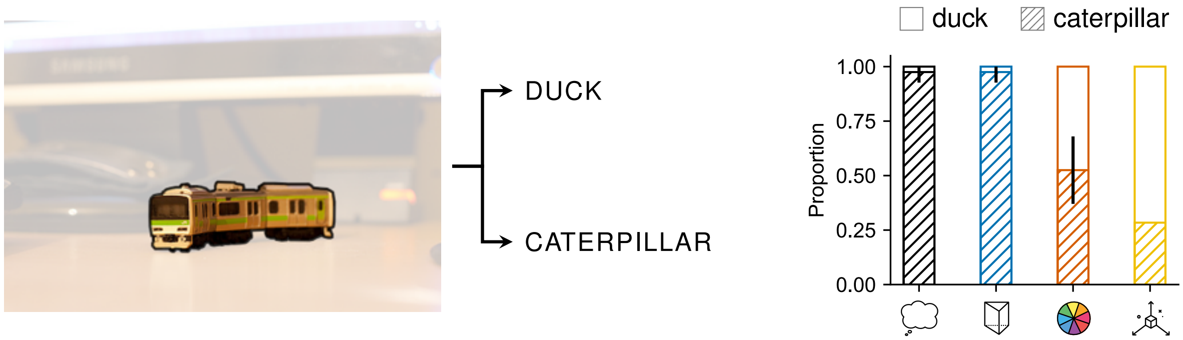

Figure S26: For the specific item (train→[duck/caterpillar], left panel), the proportion of participant choosing one of the two options in the PRETENSE, SHAPE, and COLOR conditions, as well as representational similarity derived from CLIP embedding space.

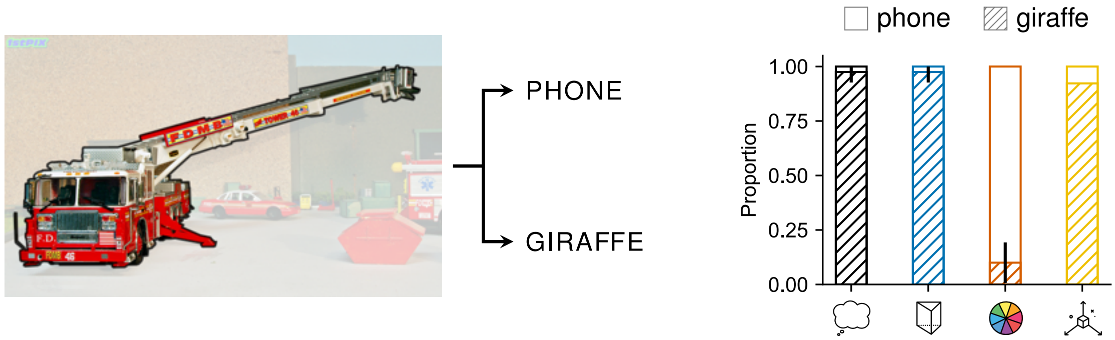

Figure S27: For the specific item (fire truck→[phone/giraffe], left panel), the proportion of participant choosing one of the two options in the PRETENSE, SHAPE, and COLOR conditions, as well as representational similarity derived from CLIP embedding space.

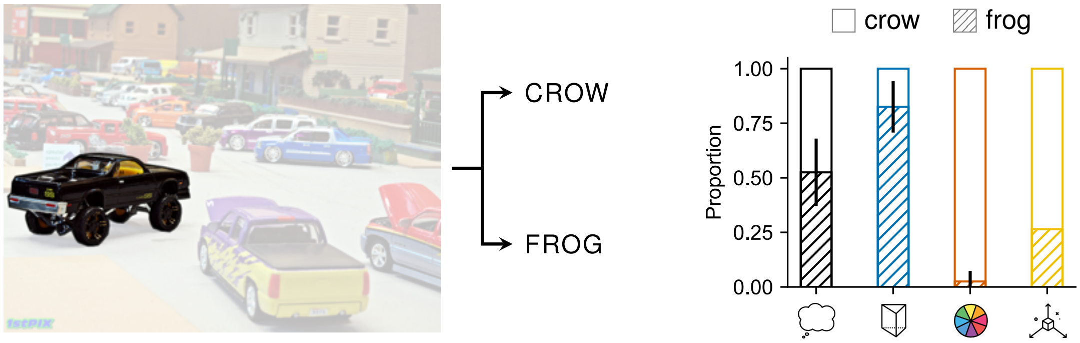

Figure S28: For the specific item (car→[crow/frog], left panel), the proportion of participant choosing one of the two options in the Pretense, Shape, and Color conditions, as well as representational similarity derived from CLIP embedding space.

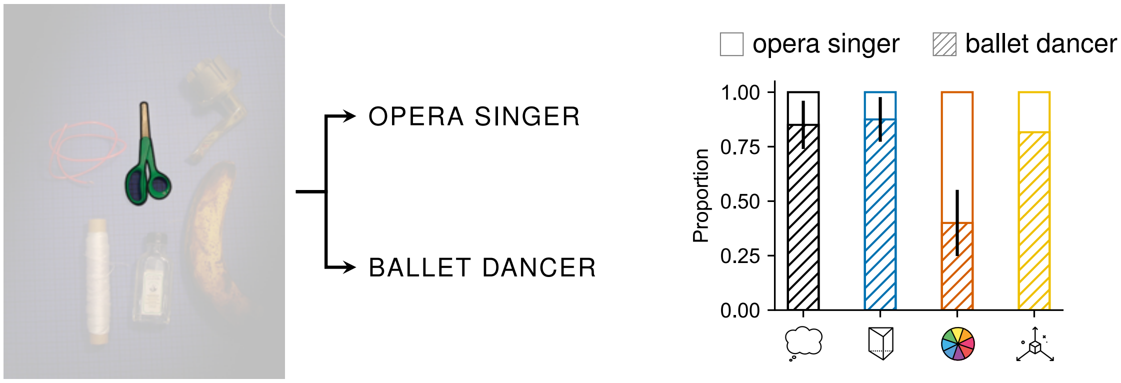

Figure S29: For the specific item (scissors→[opera singer/ballet dancer], left panel), the proportion of participant choosing one of the two options in the Pretense, Shape, and Color conditions, as well as representational similarity derived from CLIP embedding space.

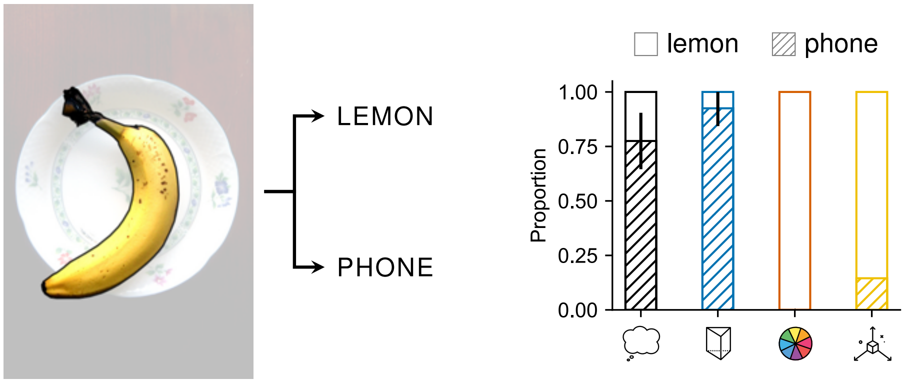

Figure S30: For the specific item (banana→[lemon/phone], left panel), the proportion of participant choosing one of the two options in the Pretense, Shape, and Color conditions, as well as representational similarity derived from CLIP embedding space.

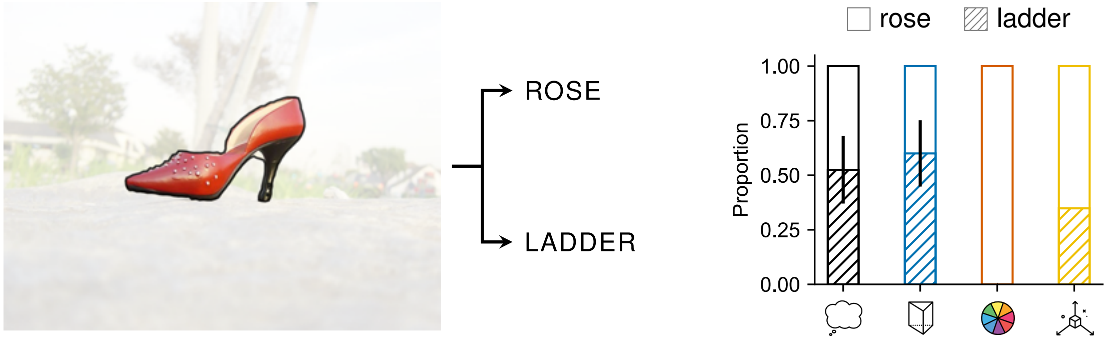

Figure S31: For the specific item (high heel→[rose/ladder], left panel), the proportion of participant choosing one of the two options in the PRETENSE, SHAPE, and COLOR conditions, as well as representational similarity derived from CLIP embedding space.

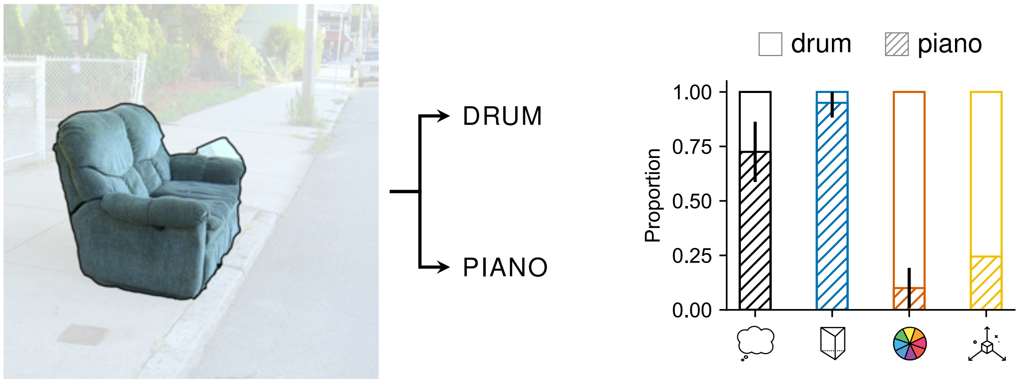

Figure S32: For the specific item (studio couch→[drum/piano], left panel), the proportion of participant choosing one of the two options in the PRETENSE, SHAPE, and COLOR conditions, as well as representational similarity derived from CLIP embedding space.

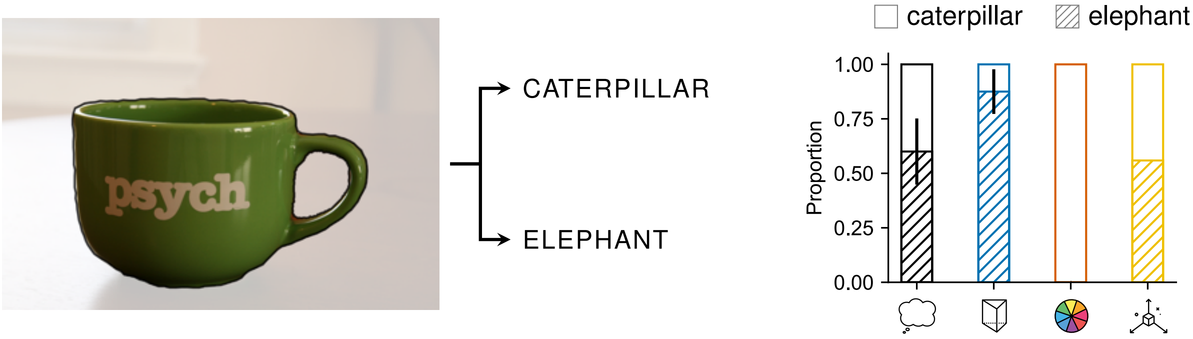

Figure S33: For the specific item (mug→[caterpillar/elephant], left panel), the proportion of participant choosing one of the two options in the PRETENSE, SHAPE, and COLOR conditions, as well as representational similarity derived from CLIP embedding space.

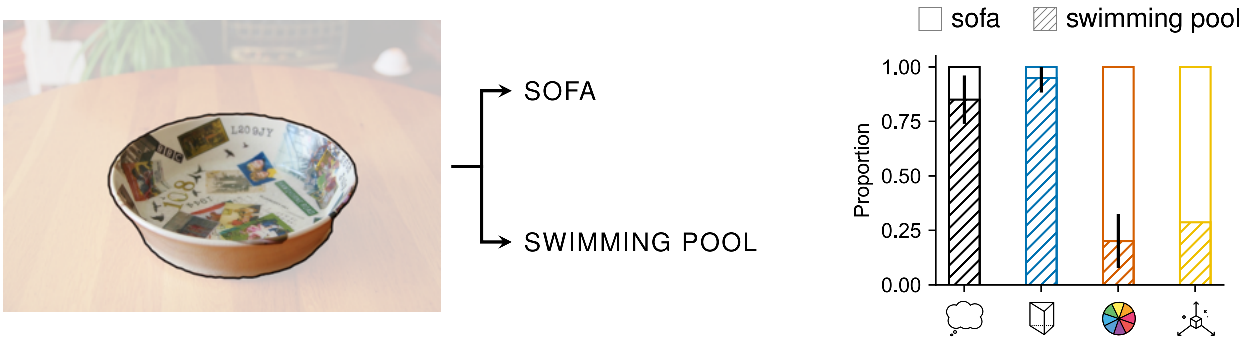

Figure S34: For the specific item (bowl→[sofa/swimming pool], left panel), the proportion of participant choosing one of the two options in the PRETENSE, SHAPE, and COLOR conditions, as well as representational similarity derived from CLIP embedding space.

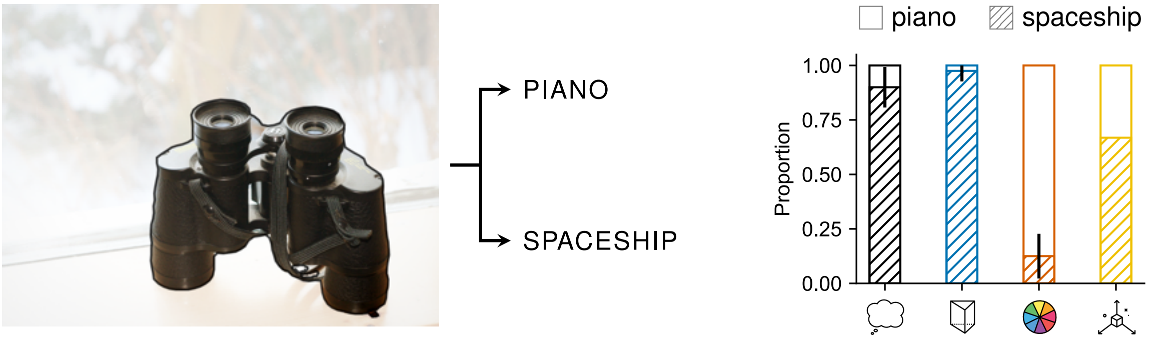

Figure S35: For the specific item (binoculars→[piano/spaceship], left panel), the proportion of participant choosing one of the two options in the PRETENSE, SHAPE, and COLOR conditions, as well as representational similarity derived from CLIP embedding space.

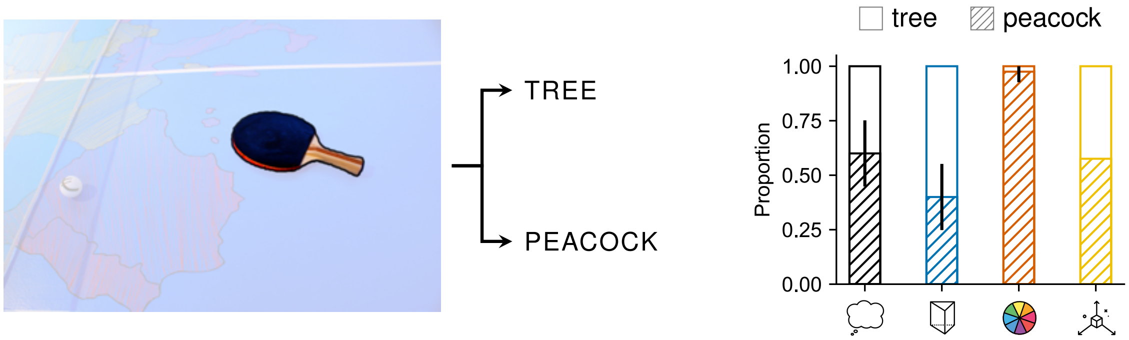

Figure S36: For the specific item (table tennis racket→[tree/peacock], left panel), the proportion of participant choosing one of the two options in the PRETENSE, SHAPE, and COLOR conditions, as well as representational similarity derived from CLIP embedding space.

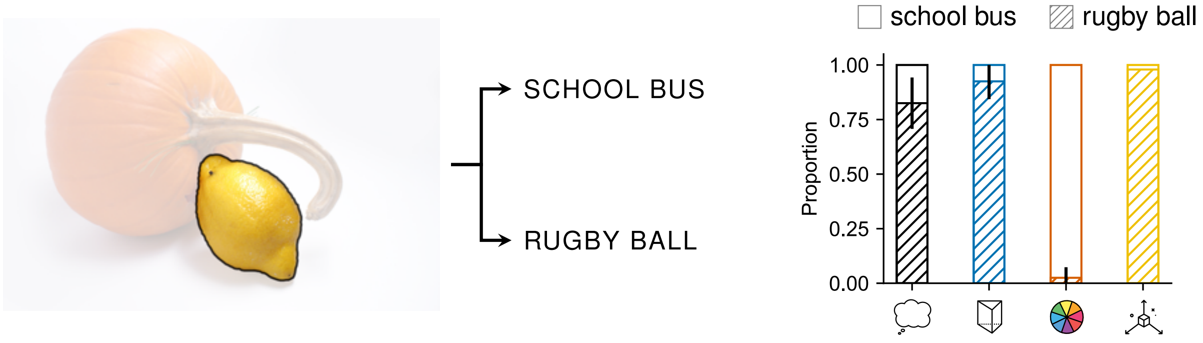

Figure S37: For the specific item (lemon→[school bus/rugby ball], left panel), the proportion of participant choosing one of the two options in the Pretense, Shape, and Color conditions, as well as representational similarity derived from CLIP embedding space.

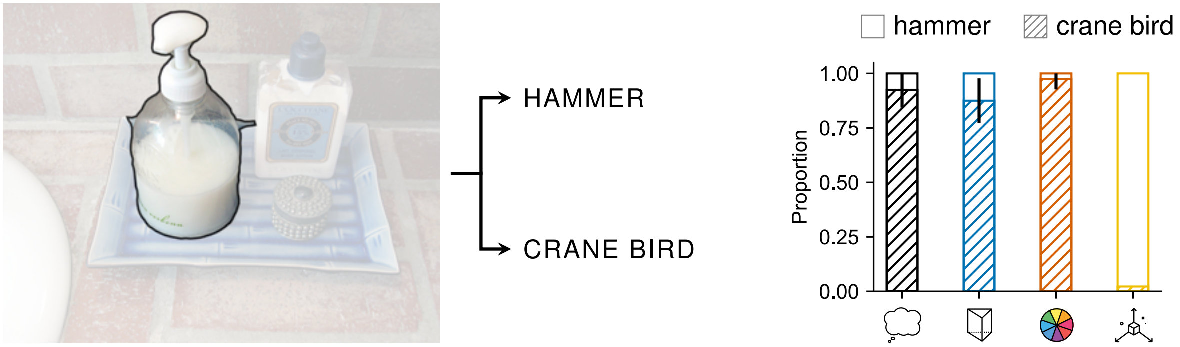

Figure S38: For the specific item (soap dispenser→[hammer/crane bird], left panel), the proportion of participant choosing one of the two options in the Pretense, Shape, and Color conditions, as well as representational similarity derived from CLIP embedding space.

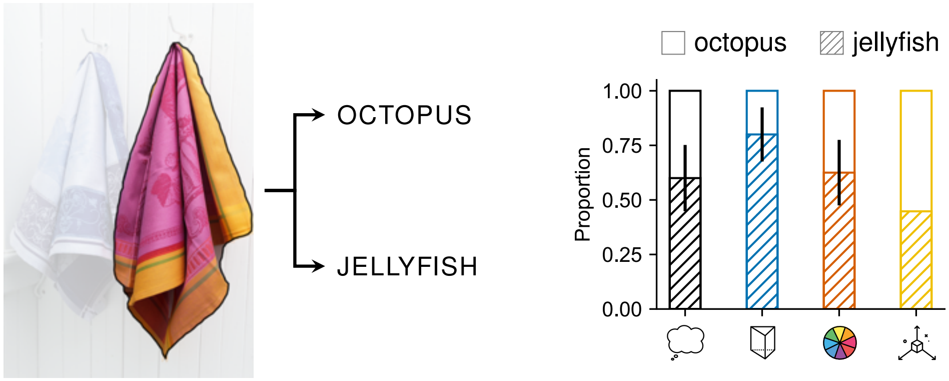

Figure S39: For the specific item (towel→[octopus/jellyfish], left panel), the proportion of participant choosing one of the two options in the Pretense, Shape, and Color conditions, as well as representational similarity derived from CLIP embedding space.

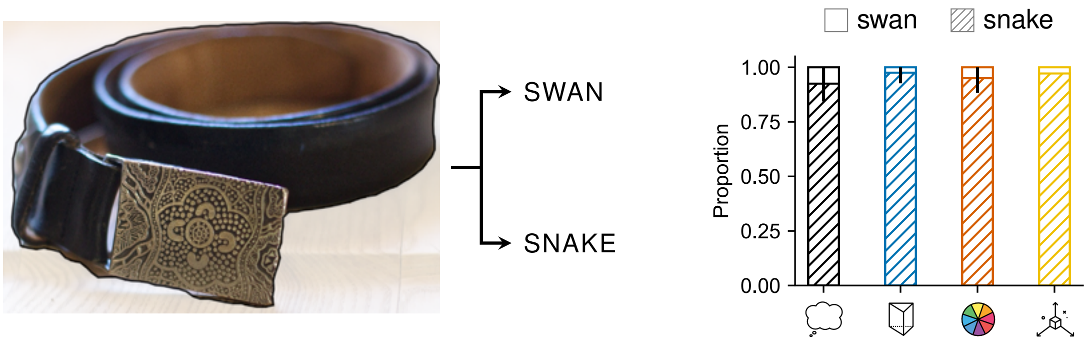

Figure S40: For the specific item (belt→[swan/snake], left panel), the proportion of participant choosing one of the two options in the PRETENSE, SHAPE, and COLOR conditions, as well as representational similarity derived from CLIP embedding space.

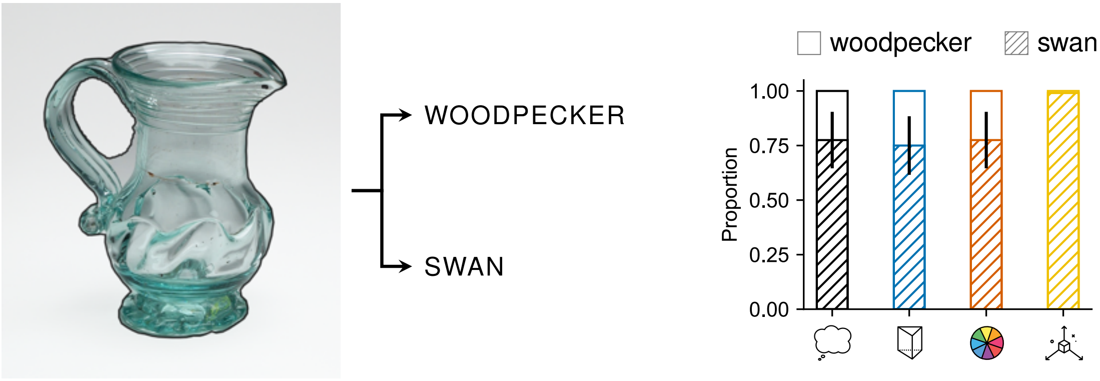

Figure S41: For the specific item (jug→[woodpecker/swan], left panel), the proportion of participant choosing one of the two options in the PRETENSE, SHAPE, and COLOR conditions, as well as representational similarity derived from CLIP embedding space.

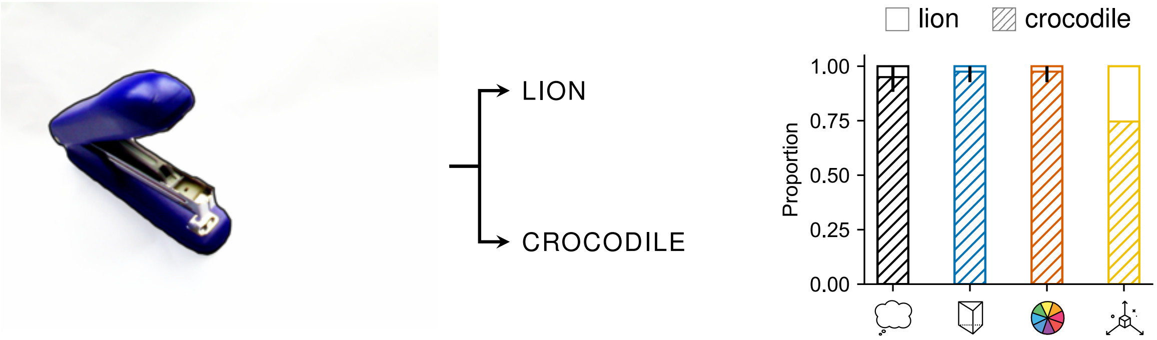

Figure S42: For the specific item (stapler→[lion/crocodile], left panel), the proportion of participant choosing one of the two options in the PRETENSE, SHAPE, and COLOR conditions, as well as representational similarity derived from CLIP embedding space.

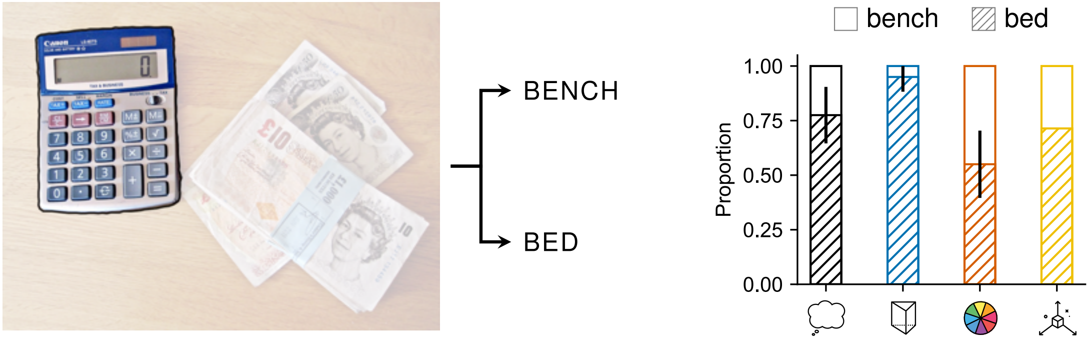

Figure S43: For the specific item (calculator→[bench/bed], left panel), the proportion of participant choosing one of the two options in the PRETENSE, SHAPE, and COLOR conditions, as well as representational similarity derived from CLIP embedding space.

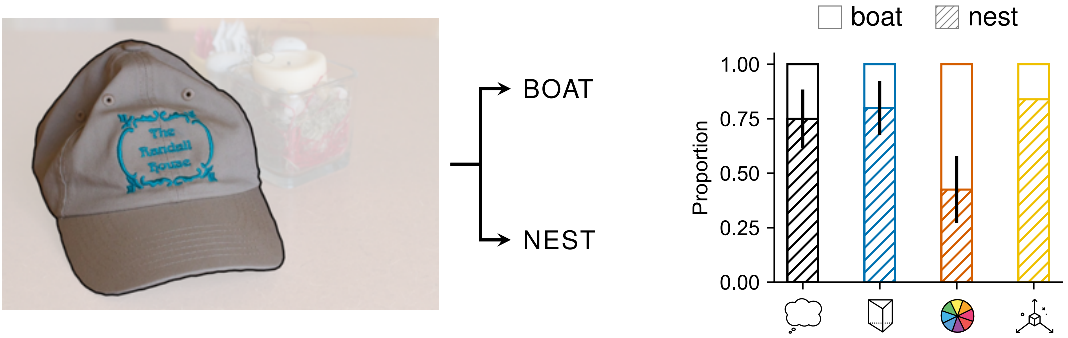

Figure S44: For the specific item (hat→[boat/nest], left panel), the proportion of participant choosing one of the two options in the PRETENSE, SHAPE, and COLOR conditions, as well as representational similarity derived from CLIP embedding space.

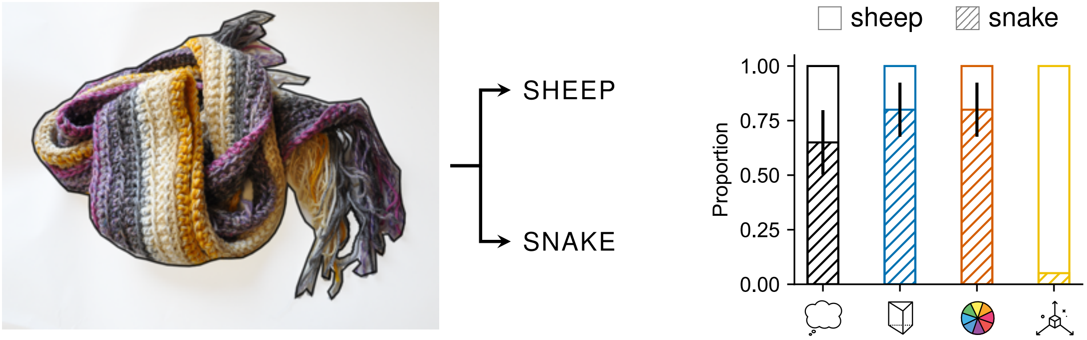

Figure S45: For the specific item (scarf→[sheep/snake], left panel), the proportion of participant choosing one of the two options in the PRETENSE, SHAPE, and COLOR conditions, as well as representational similarity derived from CLIP embedding space.

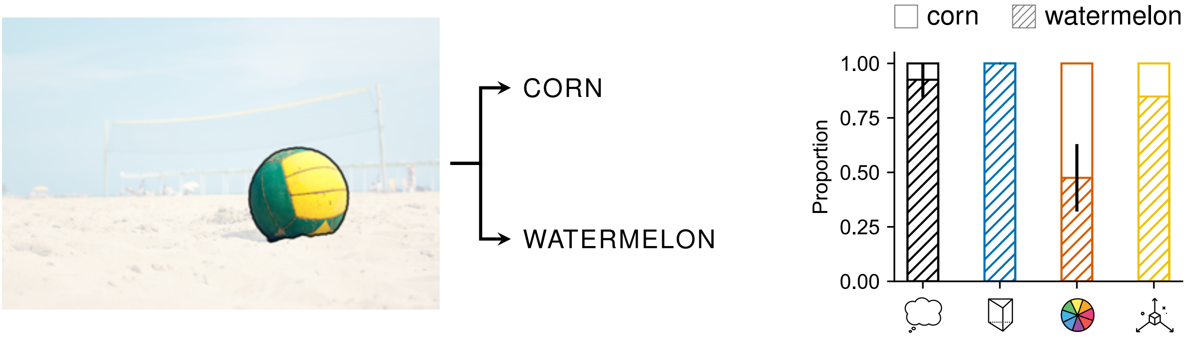

Figure S46: For the specific item (volleyball→[corn/watermelon], left panel), the proportion of participant choosing one of the two options in the PRETENSE, SHAPE, and COLOR conditions, as well as representational similarity derived from CLIP embedding space.

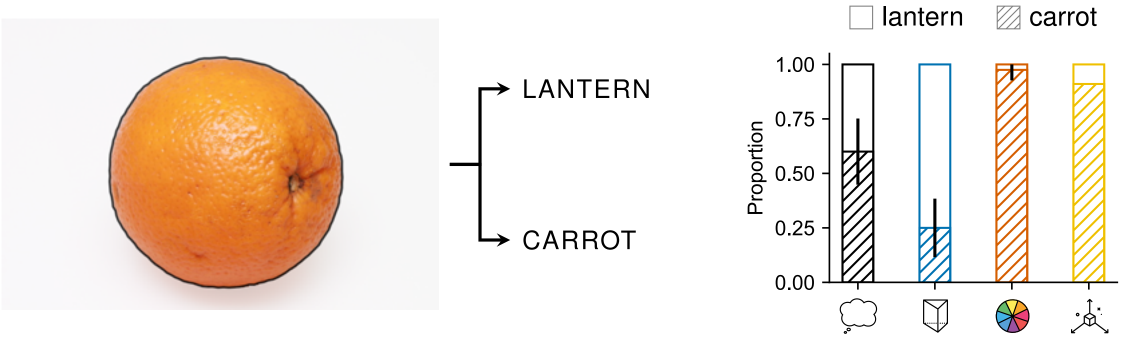

Figure S47: For the specific item (orange→[lantern/carrot], left panel), the proportion of participant choosing one of the two options in the PRETENSE, SHAPE, and COLOR conditions, as well as representational similarity derived from CLIP embedding space.

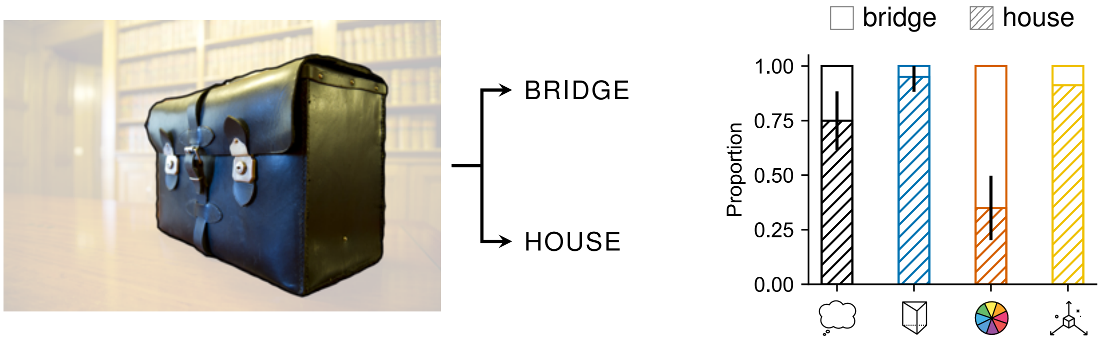

Figure S48: For the specific item (briefcase→[bridge/house], left panel), the proportion of participant choosing one of the two options in the PRETENSE, SHAPE, and COLOR conditions, as well as representational similarity derived from CLIP embedding space.

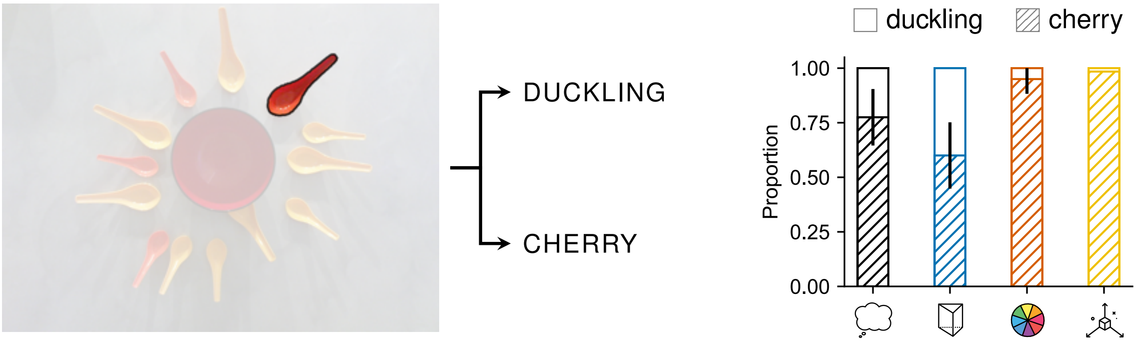

Figure S49: For the specific item (soup spoon→[duckling/cherry], left panel), the proportion of participant choosing one of the two options in the PRETENSE, SHAPE, and COLOR conditions, as well as representational similarity derived from CLIP embedding space.

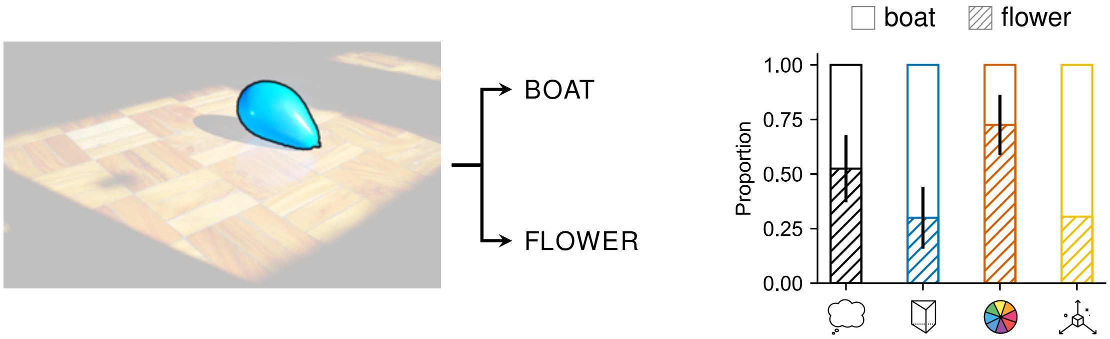

Figure S50: For the specific item (balloon→[boat/flower], left panel), the proportion of participant choosing one of the two options in the PRETENSE, SHAPE, and COLOR conditions, as well as representational similarity derived from CLIP embedding space.

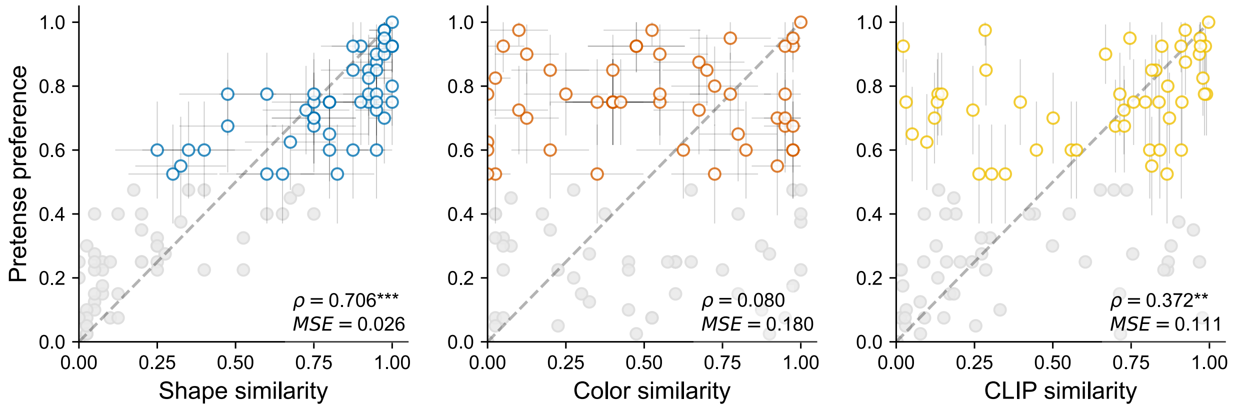

Figure S51: Correlation between pretense preferences and participant-based similarity measures of shape, color, as well as representational similarity derived from CLIP for items in Study 1. The dependent measures (choice proportion) for the two given options of each item are inherently center-symmetrical. The dis-preferred options were plotted in light gray dots, while the preferred pretend options were plotted in blue, red, and yellow for similarity preferences of shape judgment, color judgment, and CLIP embedding space. Error bars indicate 95% confidence intervals with normal approximation.

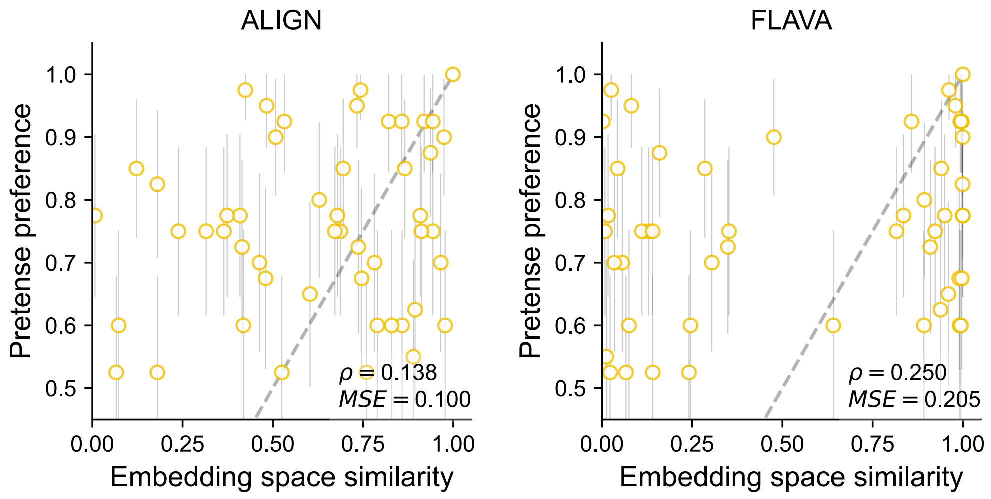

Figure S52: Correlation between pretense preferences and representational similarity derived from ALIGN and FLAVA. The dependent measures (choice proportion) for the two given options of each item are inherently center-symmetrical across both ends of the dotted-line. Spearman correlation (ρ) across items is based on the strength of preference for the preferred pretend options. Error bars indicate 95% confidence intervals with normal approximation.

C Supplemental Information for Study 2

Figure S1-S50 showcase the experimental materials used in the behavioral experiments and results from people and the multi-modal embedding space of CLIP (Radford et al., 2021). In each figure, the left panel shows the triad, the pretend object and two images of real objects. The right panel plots the proportion of participant choosing one of the two images of real objects in the PRETENSE, SHAPE, and COLOR conditions, as well as representational similarity derived from CLIP embedding space. See *Methods* section in the main text for details of the experimental procedure. All the error bars represent 95% confidence interval with normal approximation. Figure S103 plots people's pretense preferences against feature judgments in terms of shape and color, as well as representational similarity in CLIP embedding space, across all items. Figure S104 plots people's pretense preferences for items in Study 2 against representational similarity in the learned embedding space of two other representative multi-modal foundation models, ALIGN and FLAVA.

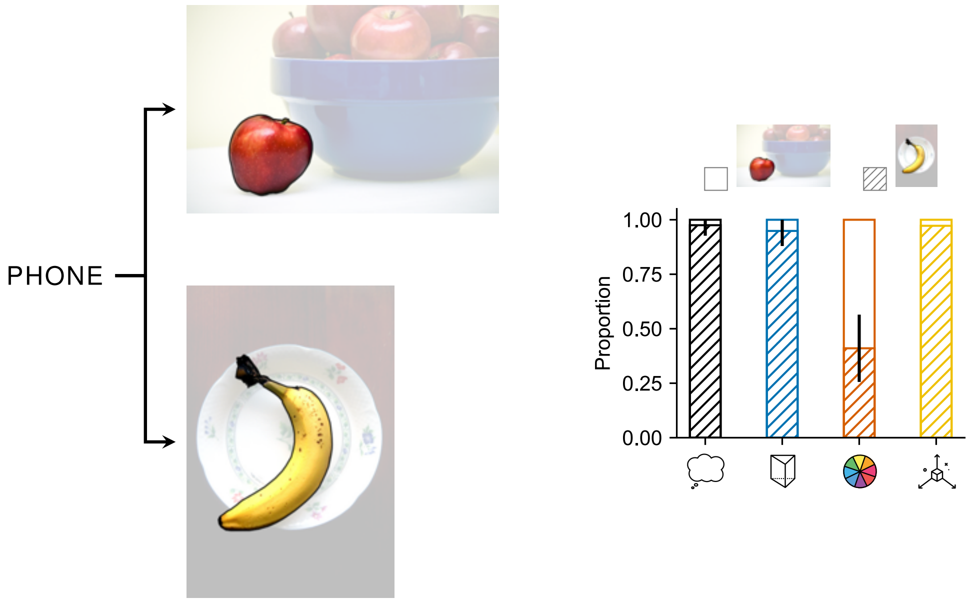

Figure S53: For the item (phone→[apple/banana], left panel) in Study 2, the proportion of participant choosing one of the two options in the Pretense, Shape, and Color conditions, as well as representational similarity derived from CLIP embedding space.

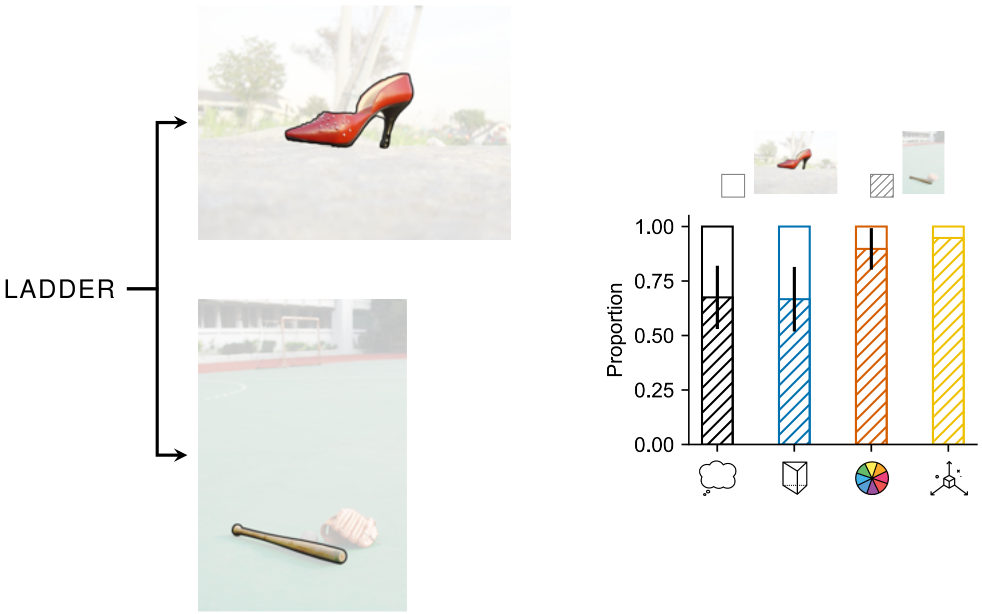

Figure S54: For the item (ladder→[high heel/baseball bat], left panel) in Study 2, the proportion of participant choosing one of the two options in the Pretense, Shape, and Color conditions, as well as representational similarity derived from CLIP embedding space.

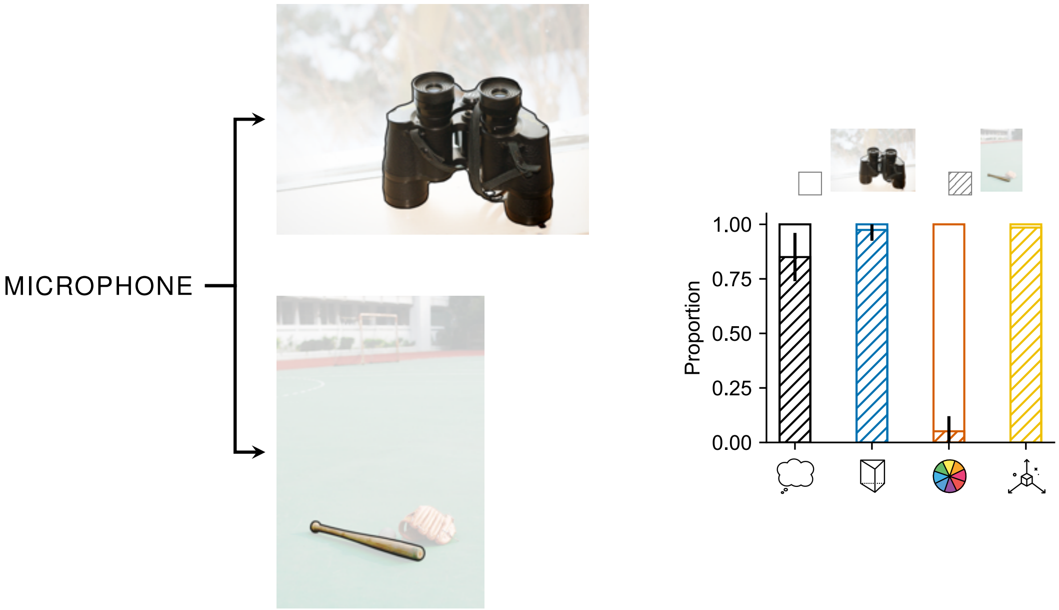

Figure S55: For the item (microphone→[binoculars/baseball bat], left panel) in Study 2, the proportion of participant choosing one of the two options in the Pretense, Shape, and Color conditions, as well as representational similarity derived from CLIP embedding space.

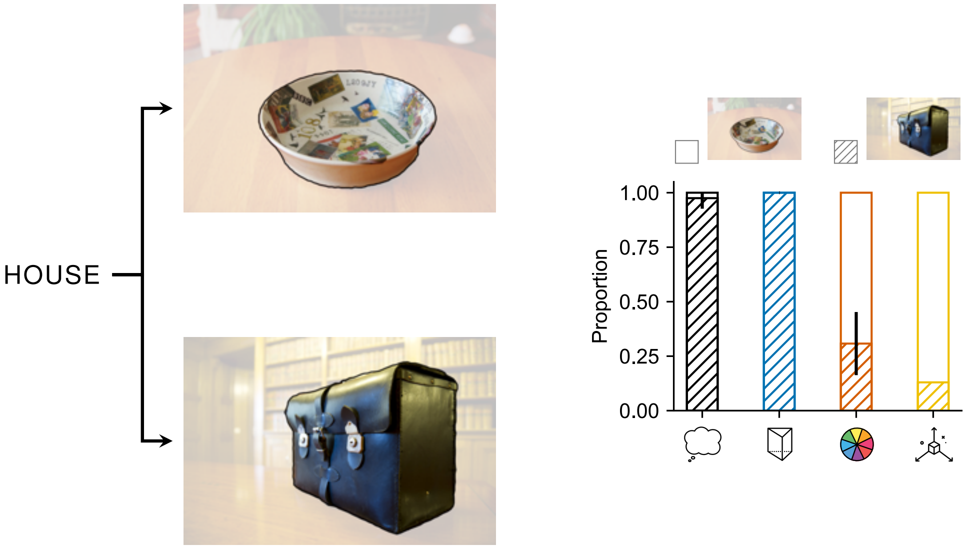

Figure S56: For the item (house→[bowl/briefcase], left panel) in Study 2, the proportion of participant choosing one of the two options in the Pretense, Shape, and Color conditions, as well as representational similarity derived from CLIP embedding space.

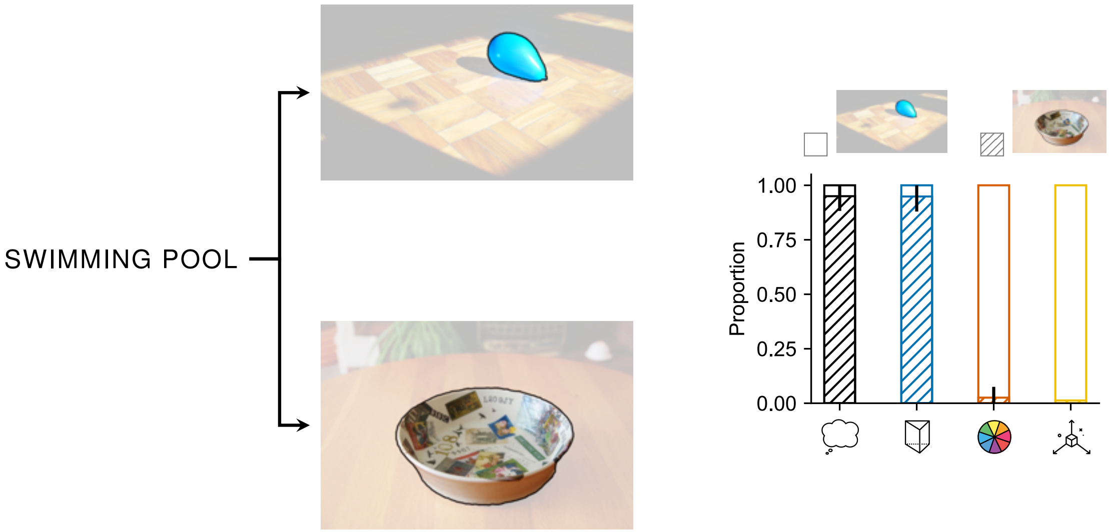

Figure S57: For the item (swimming pool→[balloon/bowl], left panel) in Study 2, the proportion of participant choosing one of the two options in the Pretense, Shape, and Color conditions, as well as representational similarity derived from CLIP embedding space.

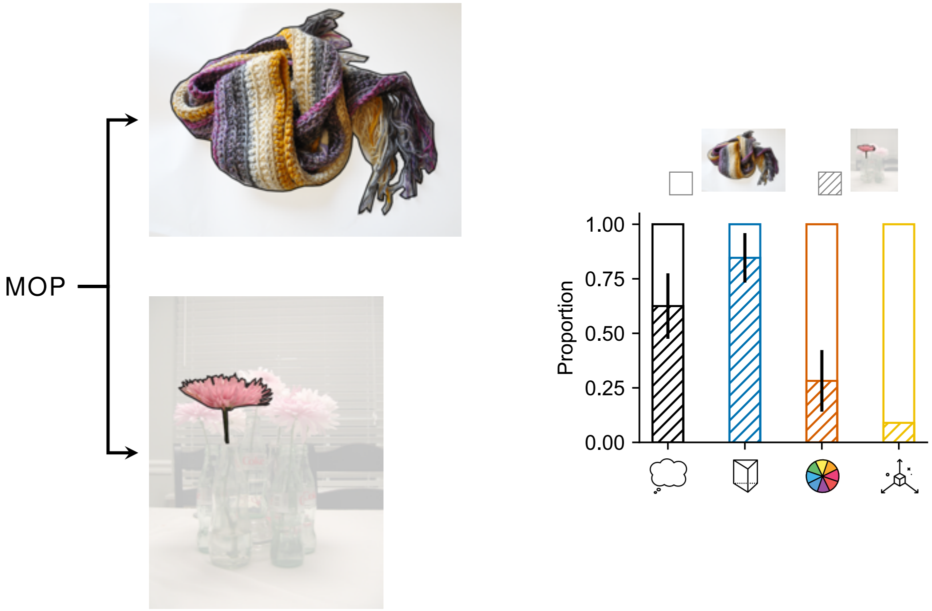

Figure S58: For the item (mop→[scarf/carnation], left panel) in Study 2, the proportion of participant choosing one of the two options in the Pretense, Shape, and Color conditions, as well as representational similarity derived from CLIP embedding space.

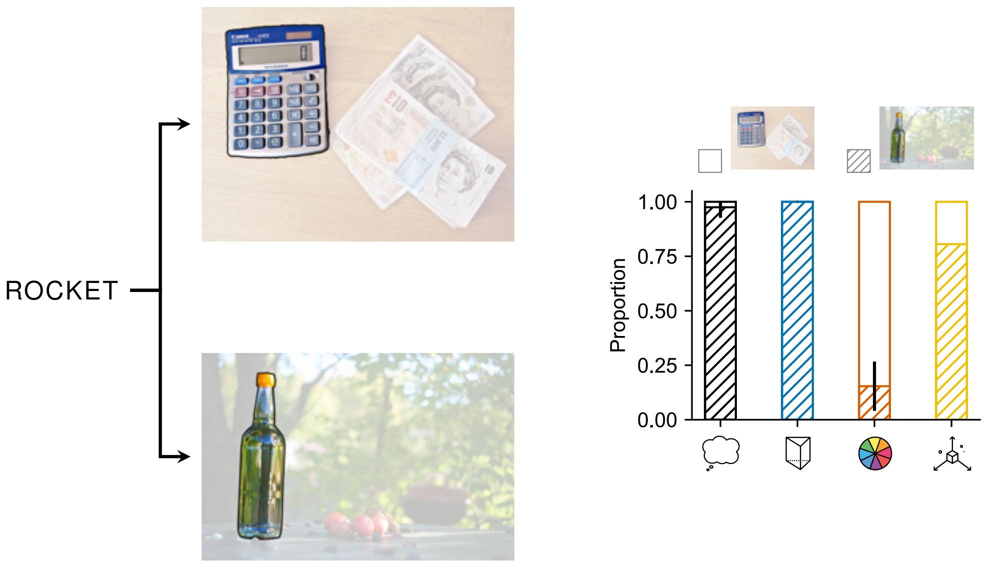

Figure S59: For the item (rocket→[calculator/bottle], left panel) in Study 2, the proportion of participant choosing one of the two options in the PRETENSE, SHAPE, and COLOR conditions, as well as representational similarity derived from CLIP embedding space.

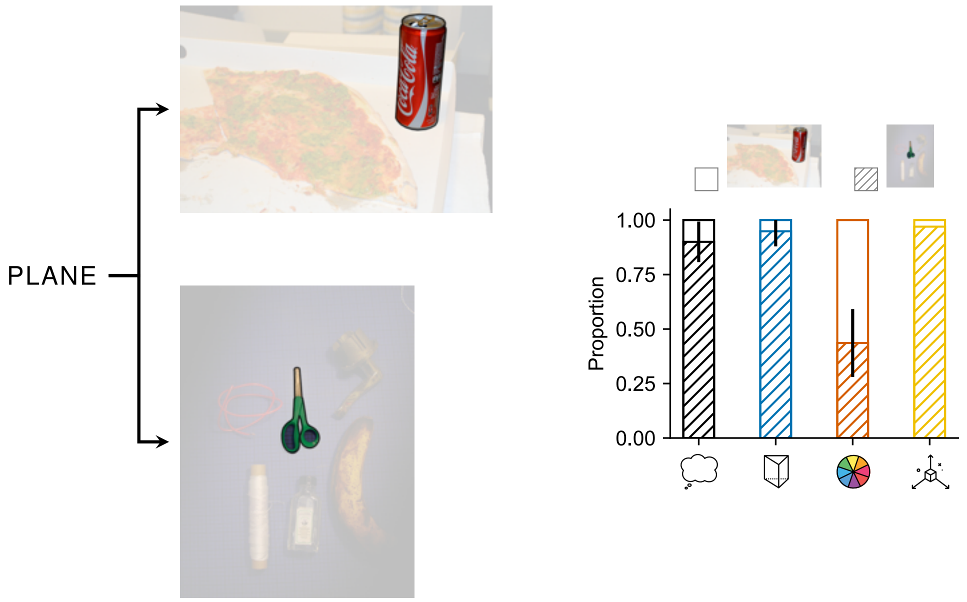

Figure S60: For the item (plane→[can/scissors], left panel) in Study 2, the proportion of participant choosing one of the two options in the PRETENSE, SHAPE, and COLOR conditions, as well as representational similarity derived from CLIP embedding space.

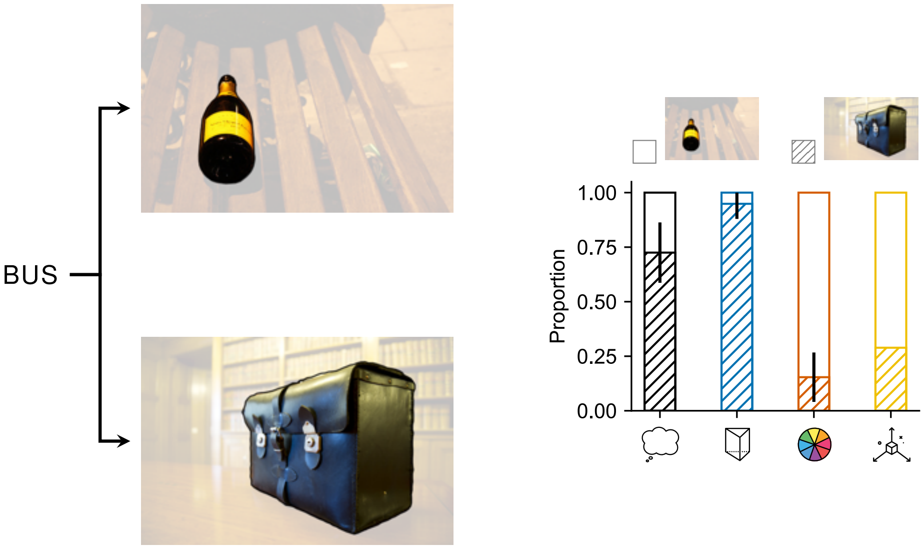

Figure S61: For the item (bus→[wine bottle/briefcase], left panel) in Study 2, the proportion of participant choosing one of the two options in the PRETENSE, SHAPE, and COLOR conditions, as well as representational similarity derived from CLIP embedding space.

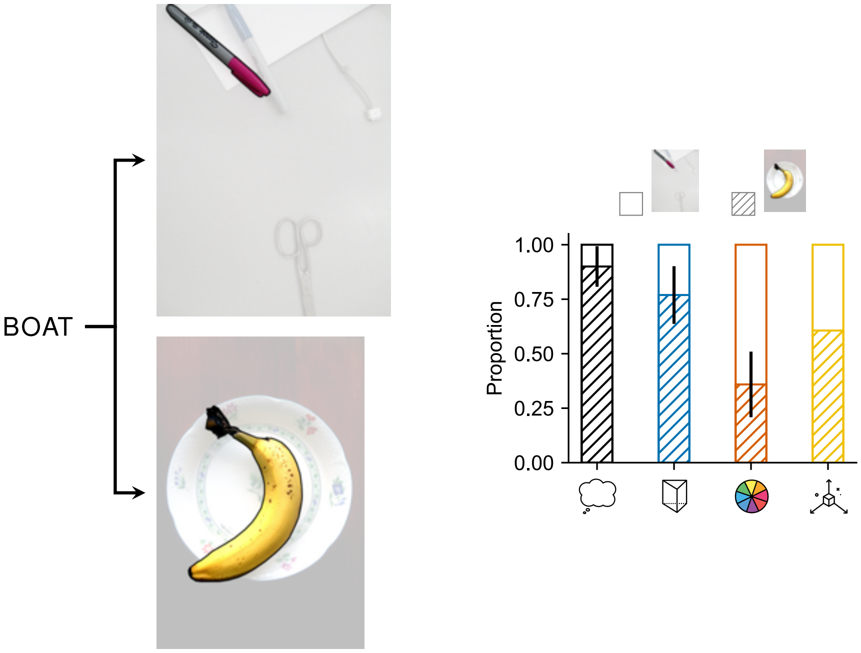

Figure S62: For the item (boat→[marker/banana], left panel) in Study 2, the proportion of participant choosing one of the two options in the PRETENSE, SHAPE, and COLOR conditions, as well as representational similarity derived from CLIP embedding space.

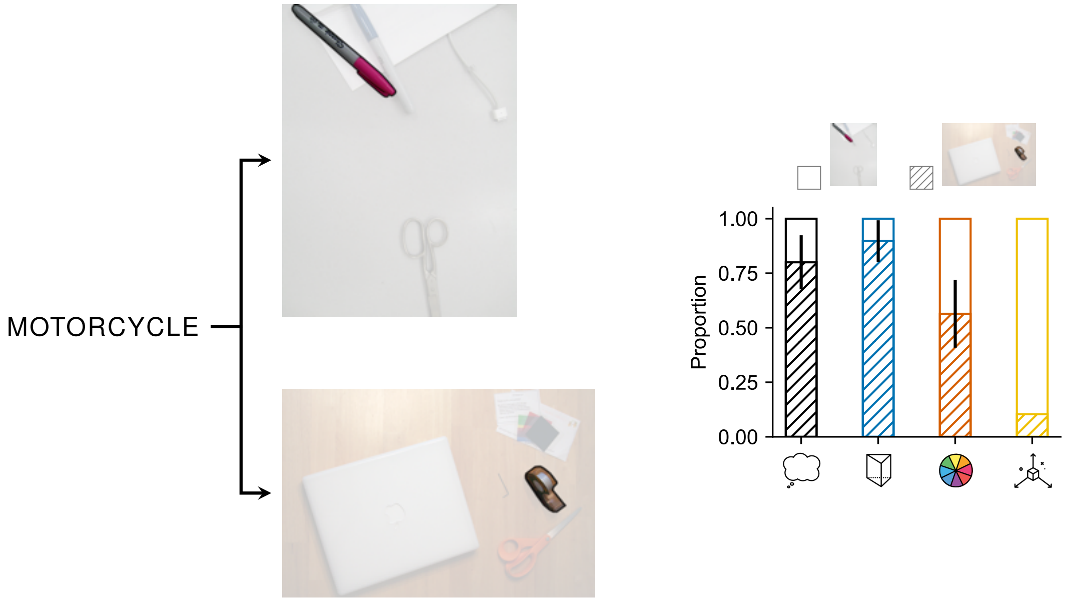

Figure S63: For the item (motorcycle→[marker/tape], left panel) in Study 2, the proportion of participant choosing one of the two options in the Pretense, Shape, and Color conditions, as well as representational similarity derived from CLIP embedding space.

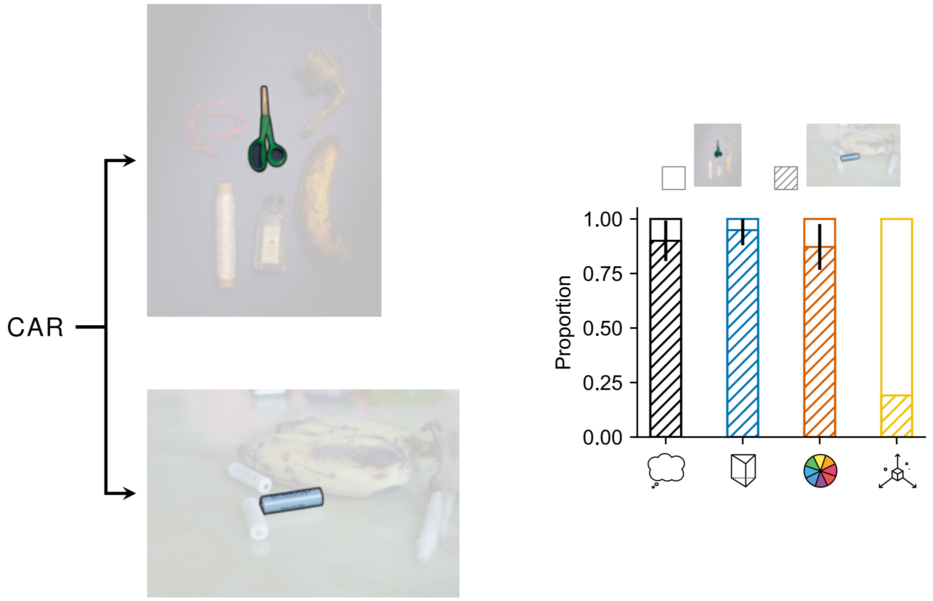

Figure S64: For the item (car→[scissors/battery], left panel) in Study 2, the proportion of participant choosing one of the two options in the Pretense, Shape, and Color conditions, as well as representational similarity derived from CLIP embedding space.

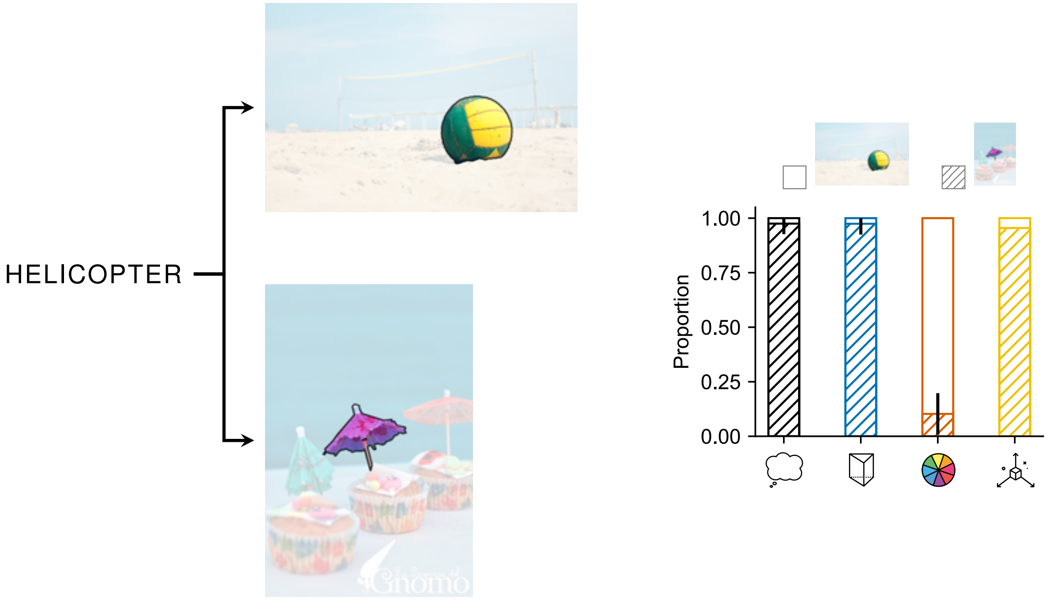

Figure S65: For the item (helicopter→[volleyball/umbrella], left panel) in Study 2, the proportion of participant choosing one of the two options in the PRETENSE, SHAPE, and COLOR conditions, as well as representational similarity derived from CLIP embedding space.

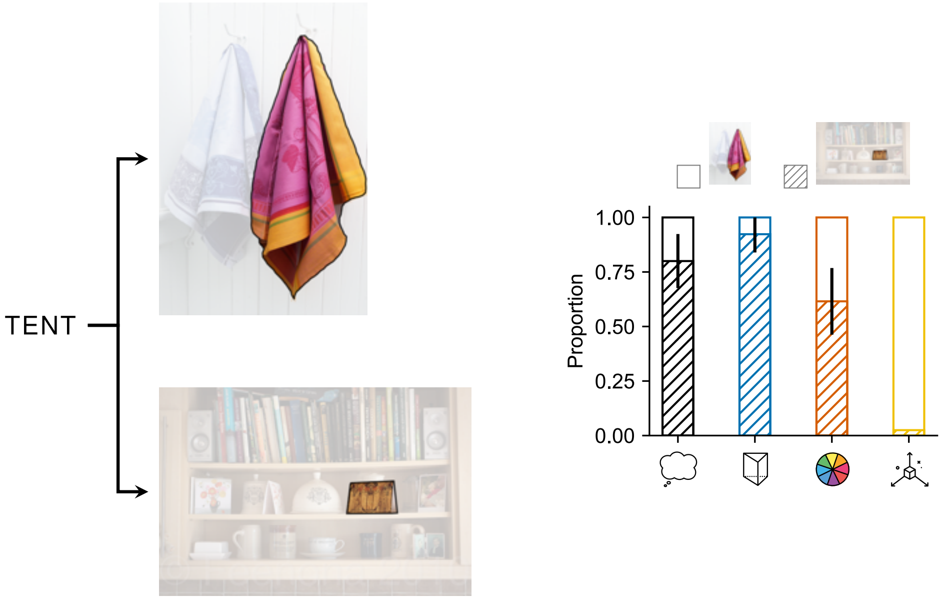

Figure S66: For the item (tent→[towel/card], left panel) in Study 2, the proportion of participant choosing one of the two options in the PRETENSE, SHAPE, and COLOR conditions, as well as representational similarity derived from CLIP embedding space.

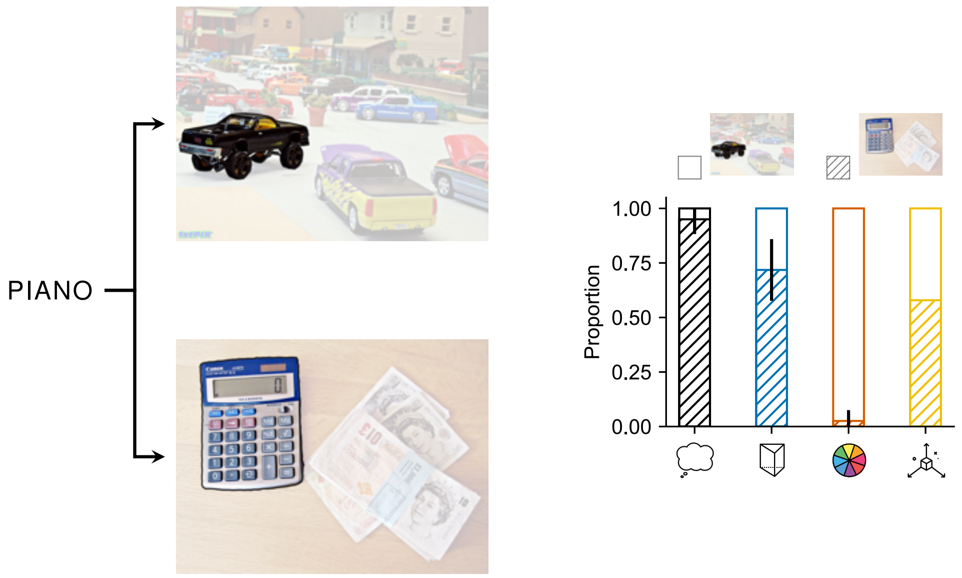

Figure S67: For the item (piano→[car/calculator], left panel) in Study 2, the proportion of participant choosing one of the two options in the Pretense, Shape, and Color conditions, as well as representational similarity derived from CLIP embedding space.

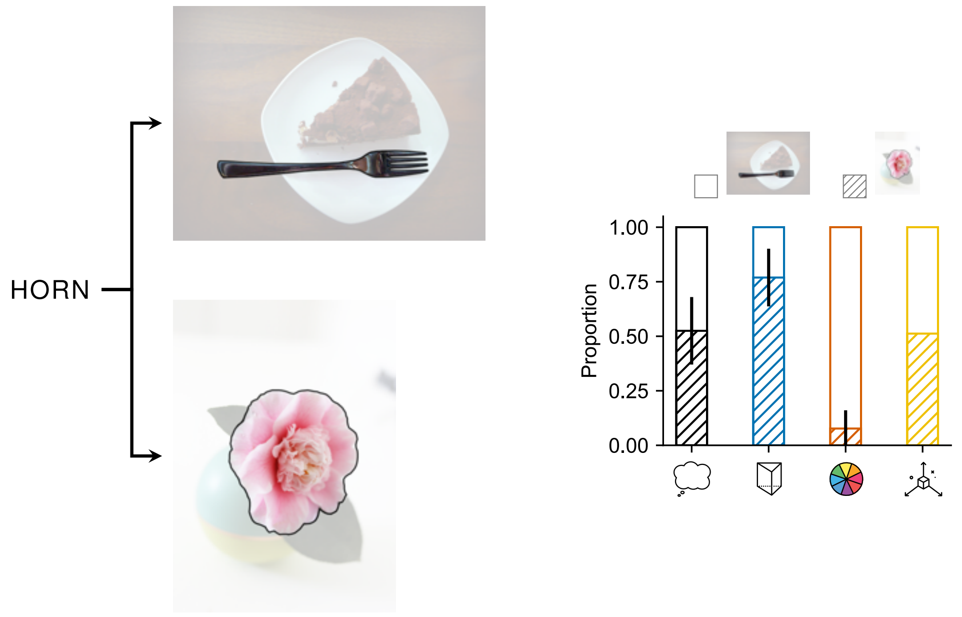

Figure S68: For the item (horn→[fork/rose], left panel) in Study 2, the proportion of participant choosing one of the two options in the Pretense, Shape, and Color conditions, as well as representational similarity derived from CLIP embedding space.

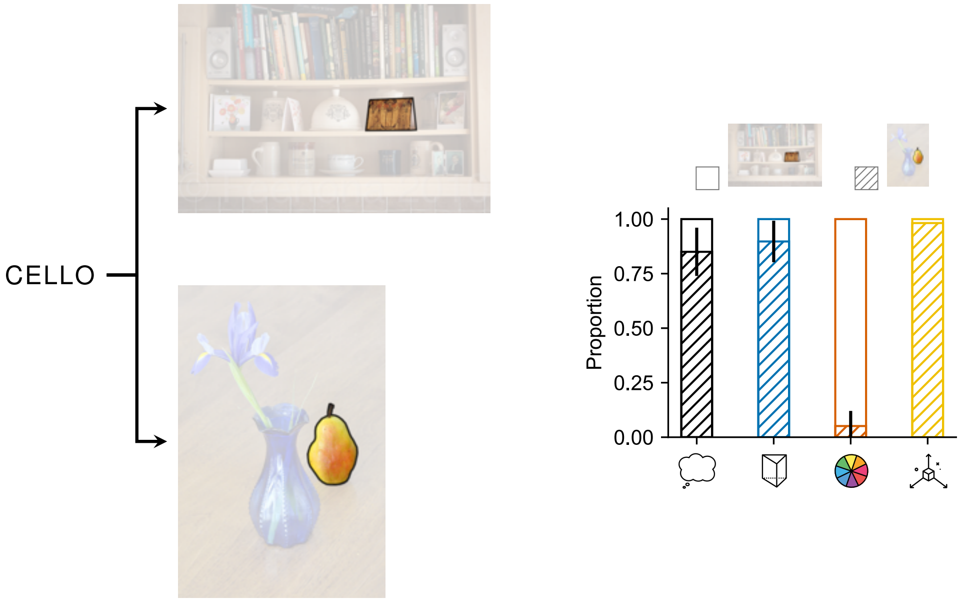

Figure S69: For the item (cello→[card/pear], left panel) in Study 2, the proportion of participant choosing one of the two options in the PRETENSE, SHAPE, and COLOR conditions, as well as representational similarity derived from CLIP embedding space.

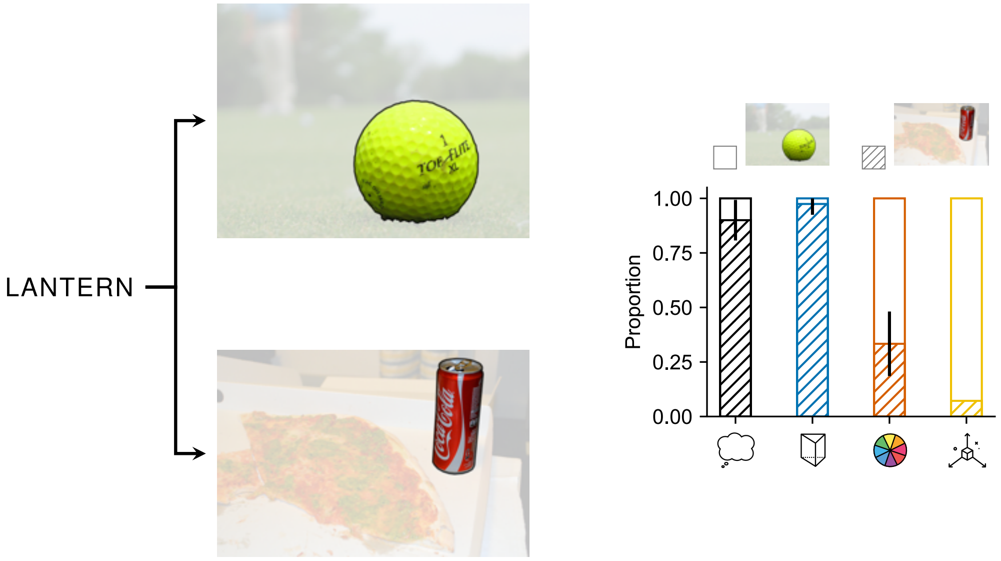

Figure S70: For the item (lantern→[golf ball/can], left panel) in Study 2, the proportion of participant choosing one of the two options in the PRETENSE, SHAPE, and COLOR conditions, as well as representational similarity derived from CLIP embedding space.

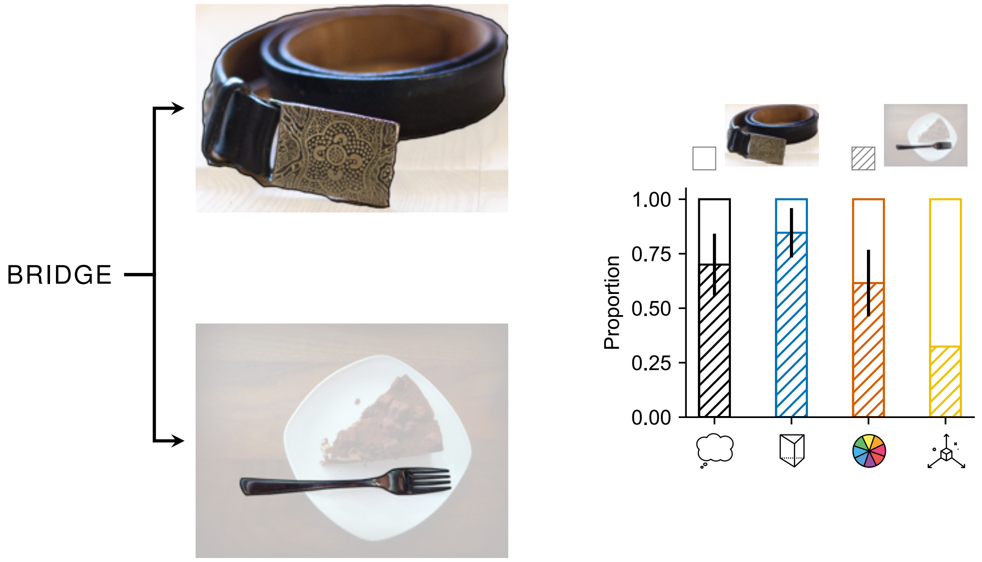

Figure S71: For the item (bridge→[belt/fork], left panel) in Study 2, the proportion of participant choosing one of the two options in the Pretense, Shape, and Color conditions, as well as representational similarity derived from CLIP embedding space.

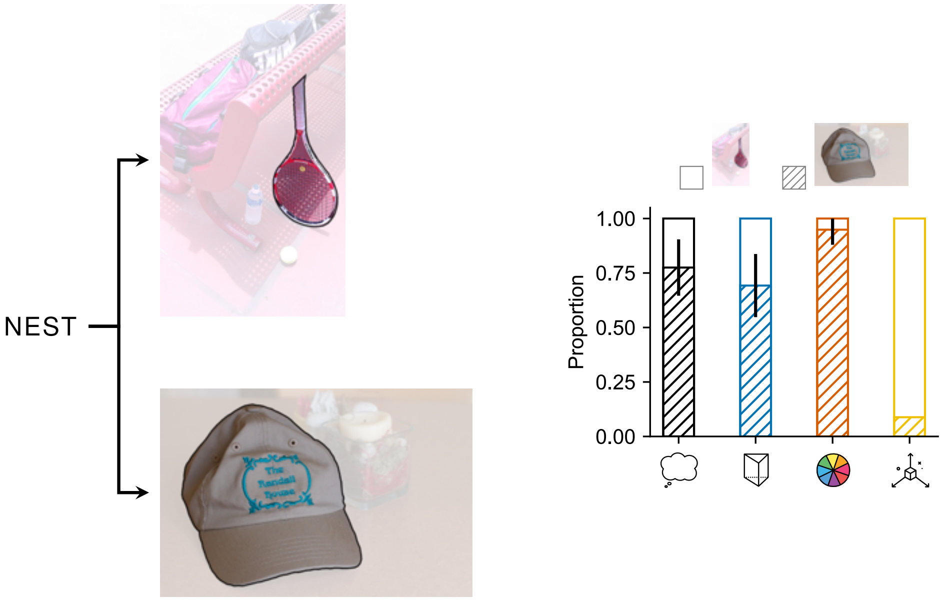

Figure S72: For the item (nest→[tennis racket/hat], left panel) in Study 2, the proportion of participant choosing one of the two options in the Pretense, Shape, and Color conditions, as well as representational similarity derived from CLIP embedding space.

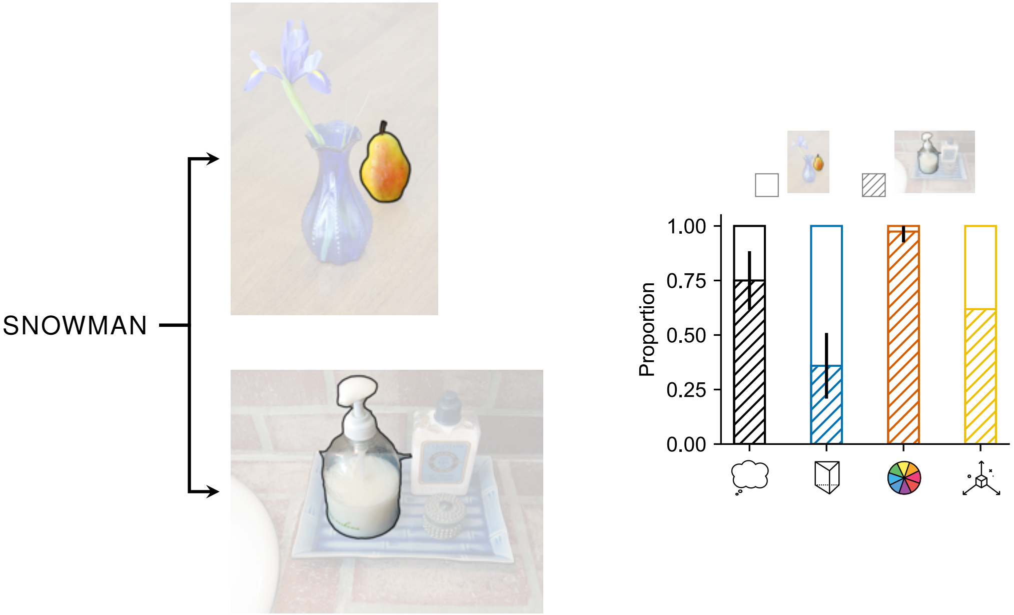

Figure S73: For the item (snowman→[pear/soap dispenser], left panel) in Study 2, the proportion of participant choosing one of the two options in the PRETENSE, SHAPE, and COLOR conditions, as well as representational similarity derived from CLIP embedding space.

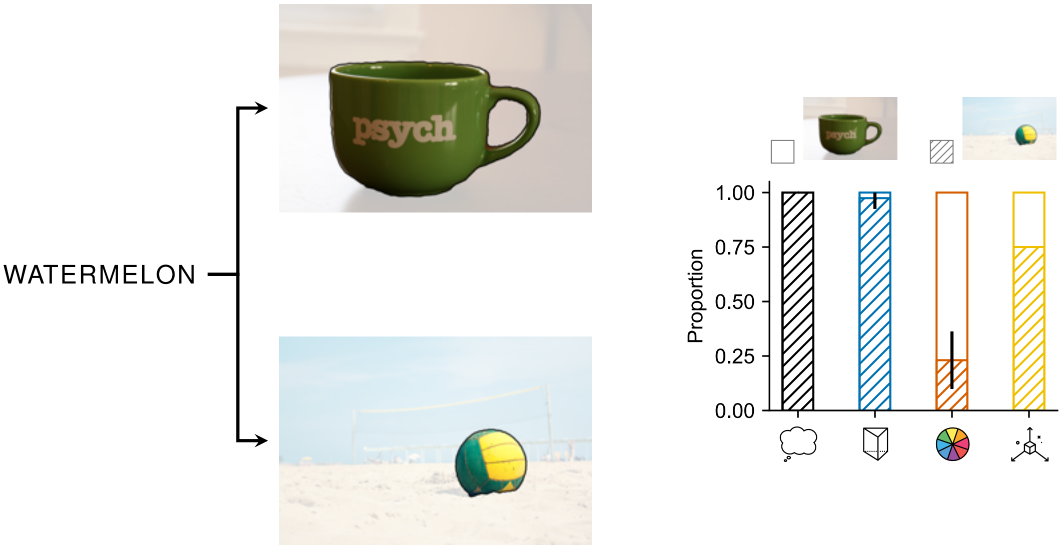

Figure S74: For the item (watermelon→[mug/volleyball], left panel) in Study 2, the proportion of participant choosing one of the two options in the PRETENSE, SHAPE, and COLOR conditions, as well as representational similarity derived from CLIP embedding space.

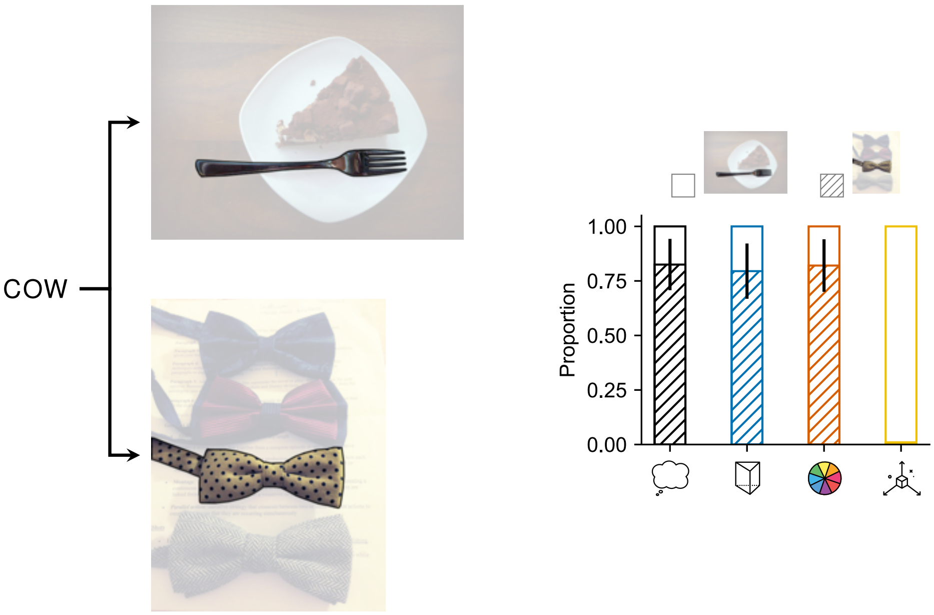

Figure S75: For the item (cow→[fork/bow-tie], left panel) in Study 2, the proportion of participant choosing one of the two options in the PRETENSE, SHAPE, and COLOR conditions, as well as representational similarity derived from CLIP embedding space.

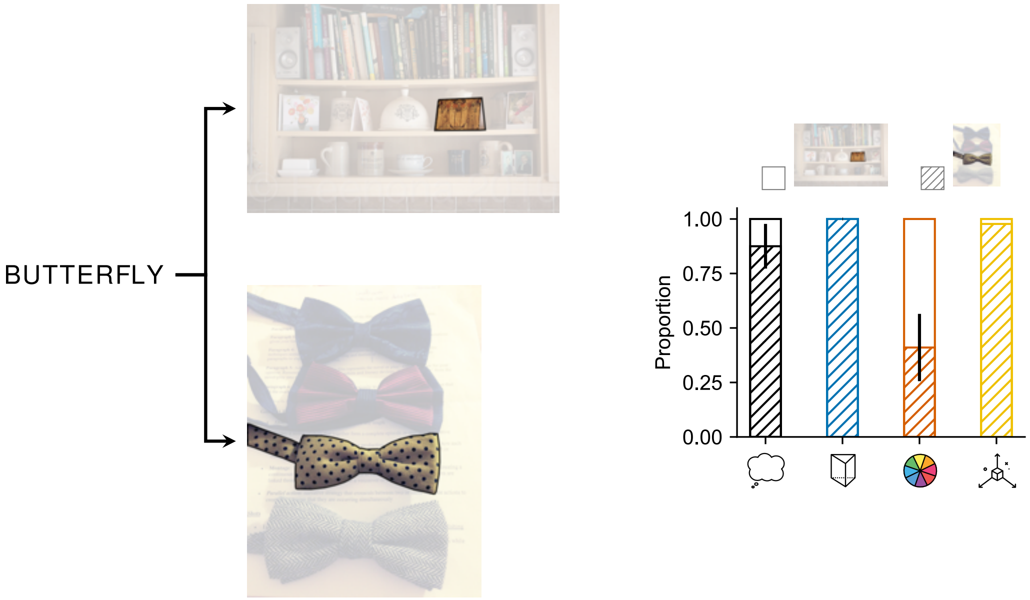

Figure S76: For the item (butterfly→[card/bow-tie], left panel) in Study 2, the proportion of participant choosing one of the two options in the PRETENSE, SHAPE, and COLOR conditions, as well as representational similarity derived from CLIP embedding space.

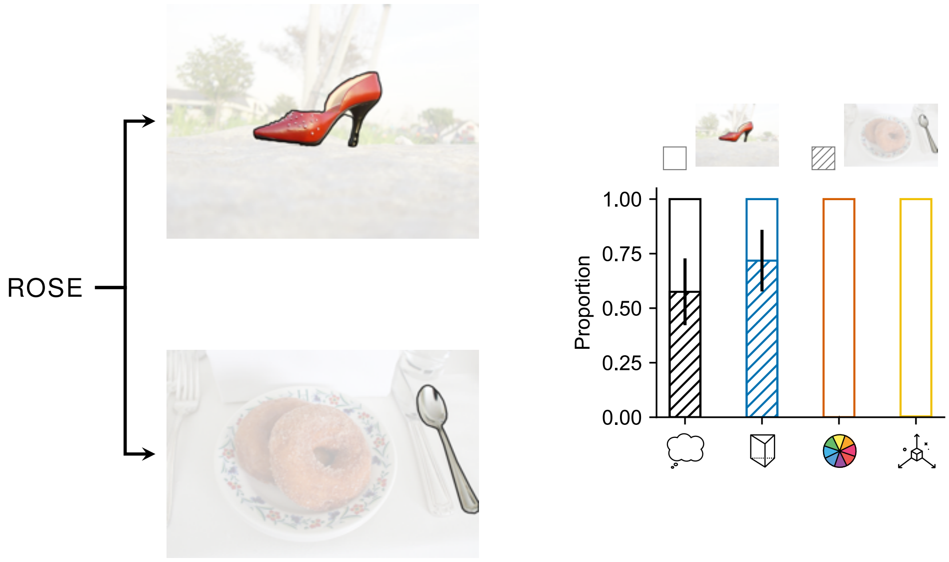

Figure S77: For the item (rose→[high heel/spoon], left panel) in Study 2, the proportion of participant choosing one of the two options in the PRETENSE, SHAPE, and COLOR conditions, as well as representational similarity derived from CLIP embedding space.

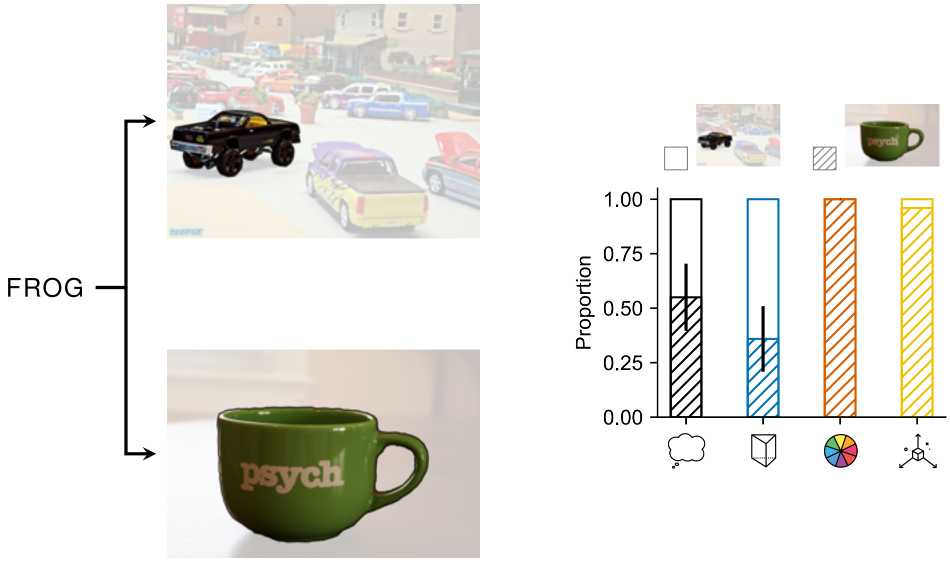

Figure S78: For the item (frog→[car/mug], left panel) in Study 2, the proportion of participant choosing one of the two options in the PRETENSE, SHAPE, and COLOR conditions, as well as representational similarity derived from CLIP embedding space.

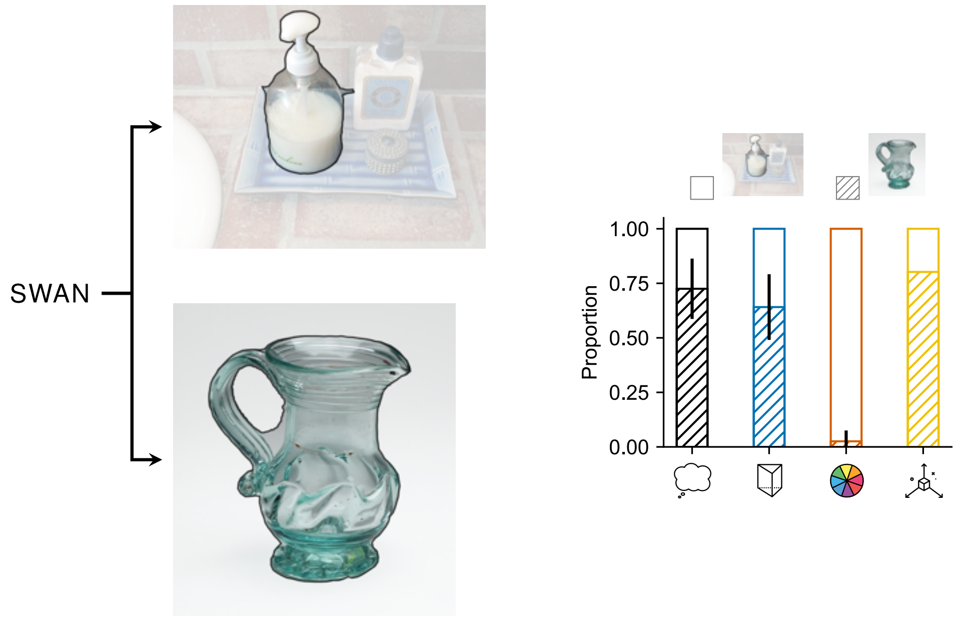

Figure S79: For the item (swan→[soap dispenser/jug], left panel) in Study 2, the proportion of participant choosing one of the two options in the PRETENSE, SHAPE, and COLOR conditions, as well as representational similarity derived from CLIP embedding space.

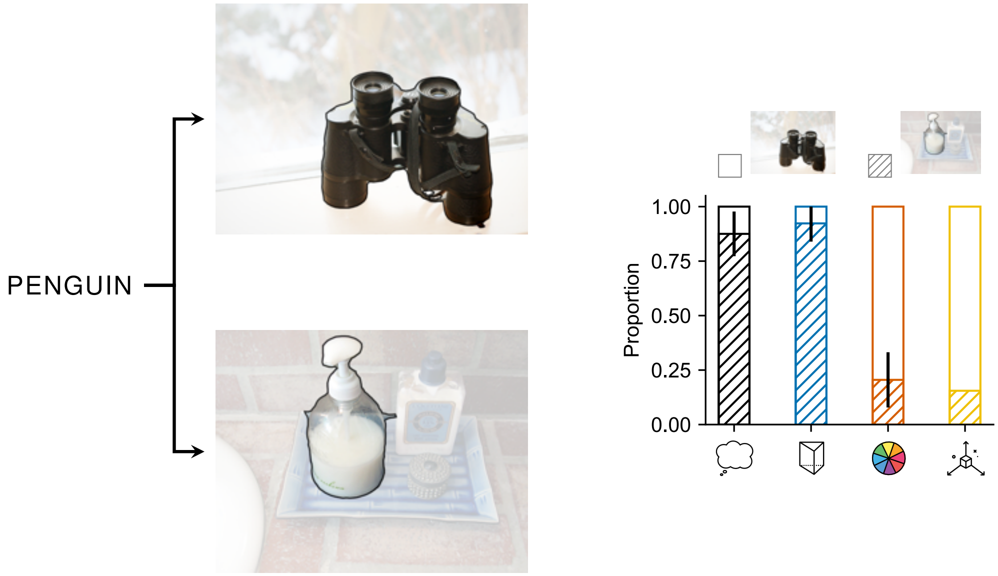

Figure S80: For the item (penguin→[binoculars/soap dispenser], left panel) in Study 2, the proportion of participant choosing one of the two options in the PRETENSE, SHAPE, and COLOR conditions, as well as representational similarity derived from CLIP embedding space.

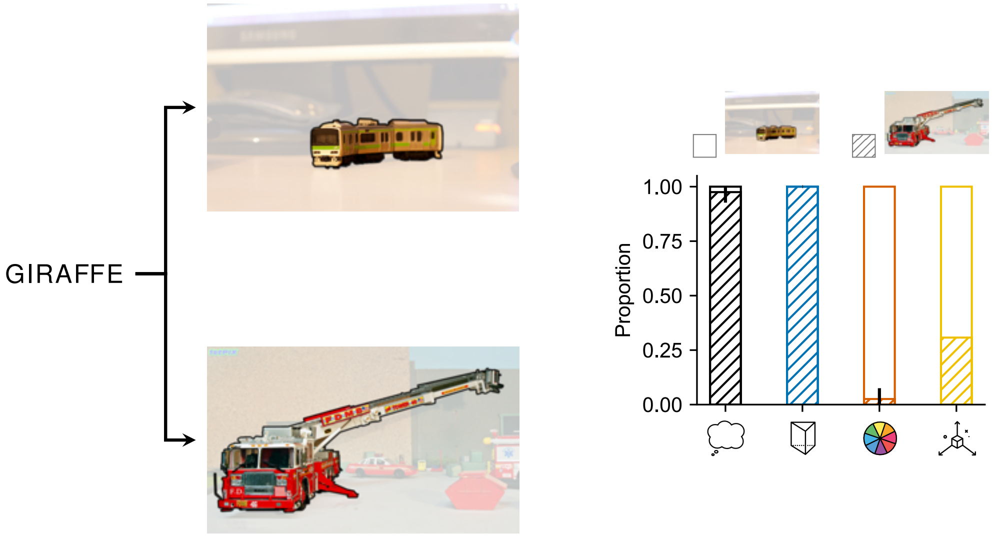

Figure S81: For the item (giraffe→[train/fire truck], left panel) in Study 2, the proportion of participant choosing one of the two options in the PRETENSE, SHAPE, and COLOR conditions, as well as representational similarity derived from CLIP embedding space.

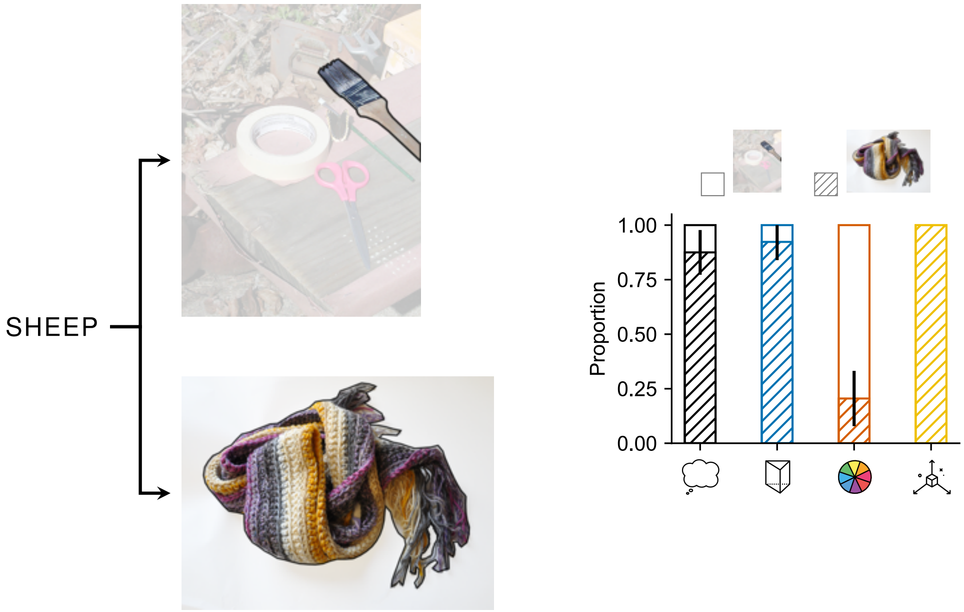

Figure S82: For the item (sheep→[paint brush/scarf], left panel) in Study 2, the proportion of participant choosing one of the two options in the PRETENSE, SHAPE, and COLOR conditions, as well as representational similarity derived from CLIP embedding space.

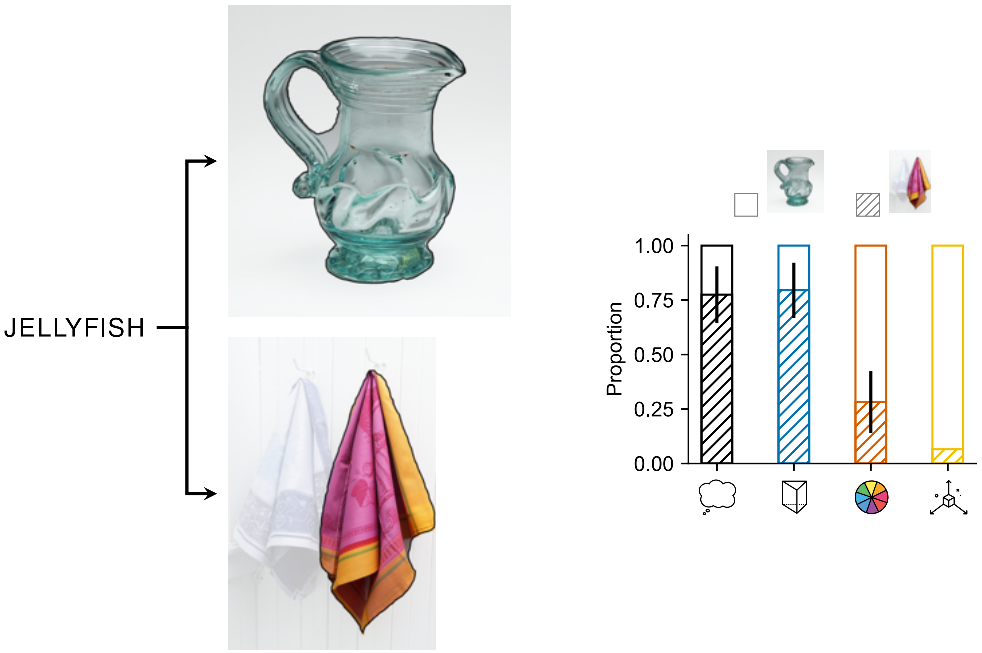

Figure S83: For the item (jellyfish→[jug/towel], left panel) in Study 2, the proportion of participant choosing one of the two options in the Pretense, Shape, and Color conditions, as well as representational similarity derived from CLIP embedding space.

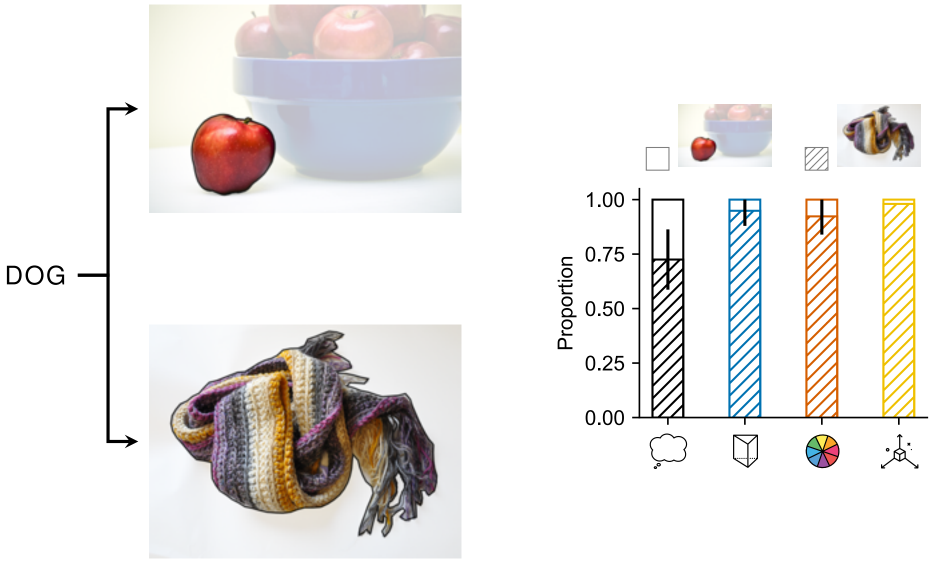

Figure S84: For the item (dog→[apple/scarf], left panel) in Study 2, the proportion of participant choosing one of the two options in the Pretense, Shape, and Color conditions, as well as representational similarity derived from CLIP embedding space.

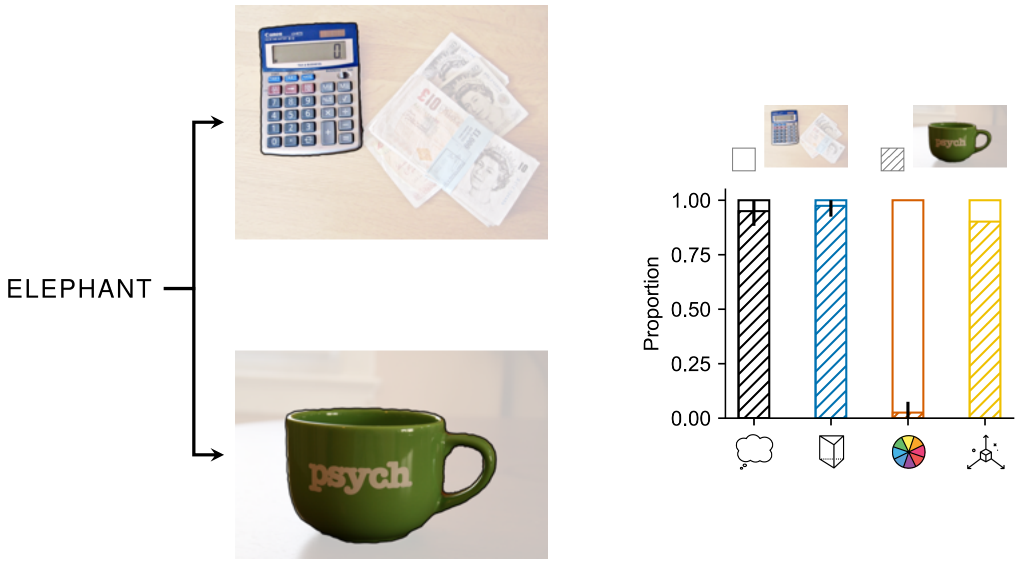

Figure S85: For the item (elephant→[calculator/mug], left panel) in Study 2, the proportion of participant choosing one of the two options in the Pretense, Shape, and Color conditions, as well as representational similarity derived from CLIP embedding space.

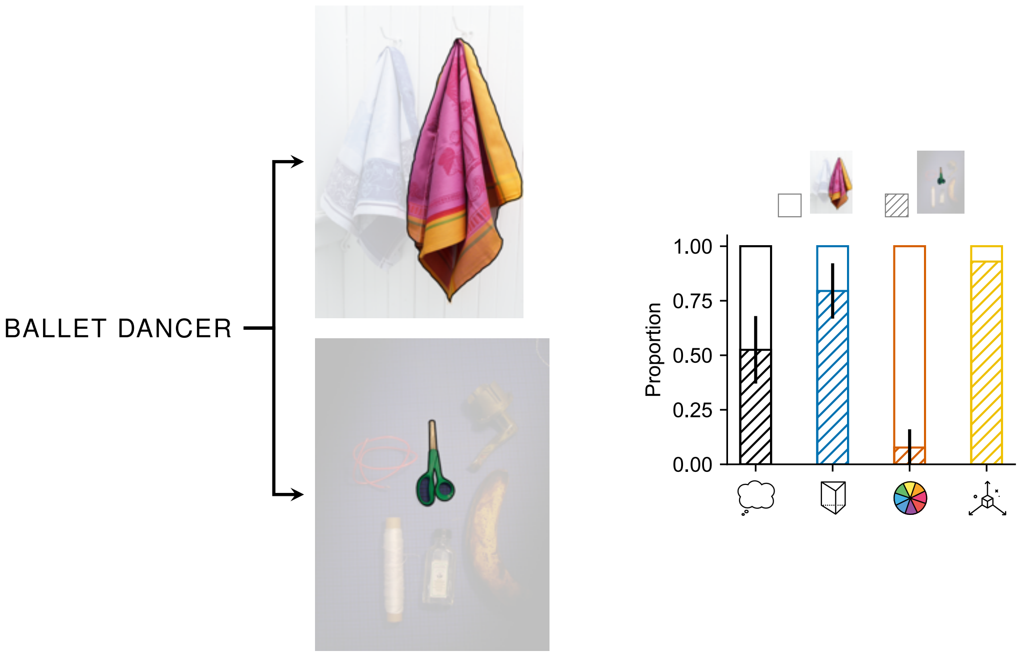

Figure S86: For the item (ballet dancer→[towel/scissors], left panel) in Study 2, the proportion of participant choosing one of the two options in the Pretense, Shape, and Color conditions, as well as representational similarity derived from CLIP embedding space.

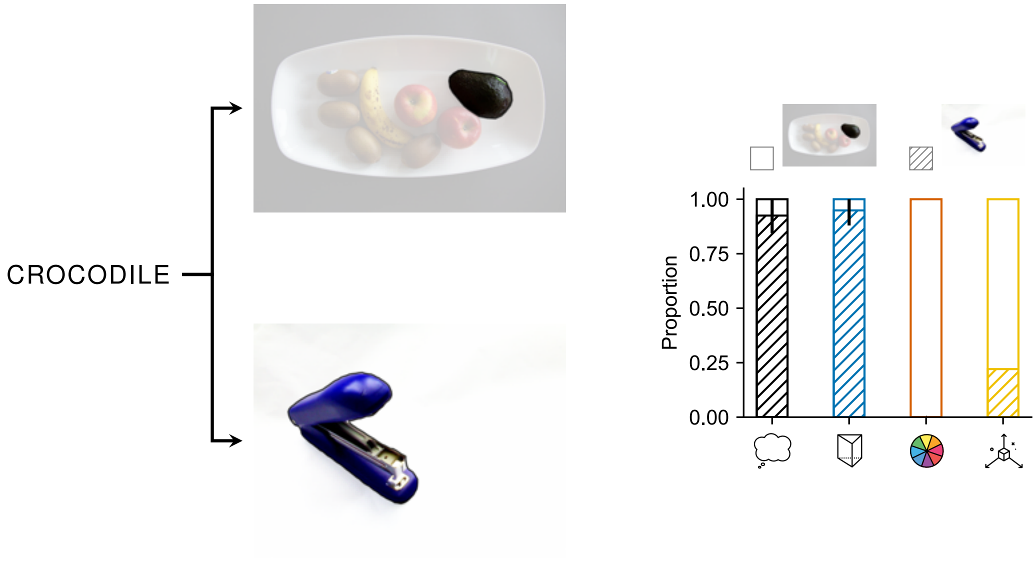

Figure S87: For the item (crocodile→[avocado/stapler], left panel) in Study 2, the proportion of participant choosing one of the two options in the PRETENSE, SHAPE, and COLOR conditions, as well as representational similarity derived from CLIP embedding space.

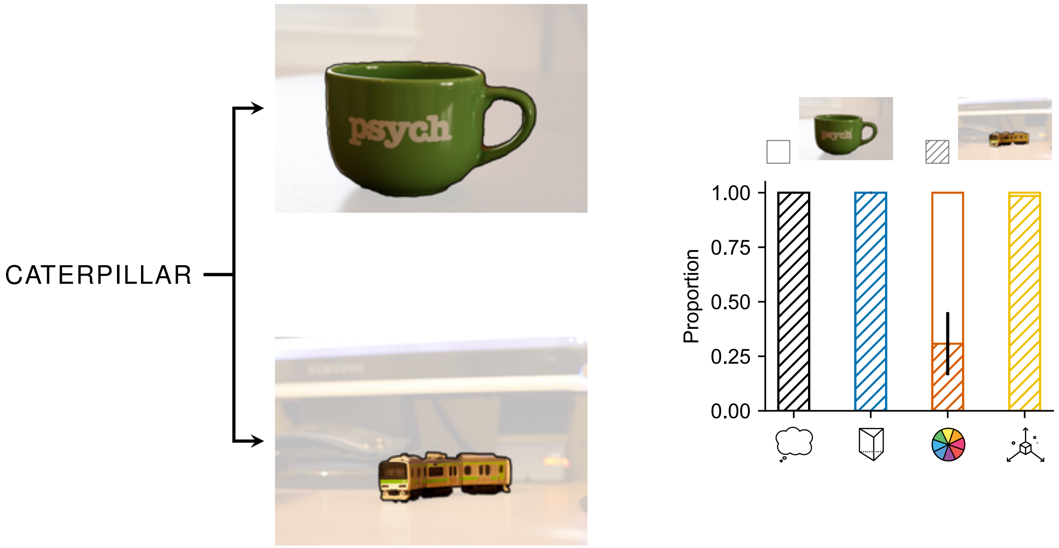

Figure S88: For the item (caterpillar→[mug/train], left panel) in Study 2, the proportion of participant choosing one of the two options in the PRETENSE, SHAPE, and COLOR conditions, as well as representational similarity derived from CLIP embedding space.

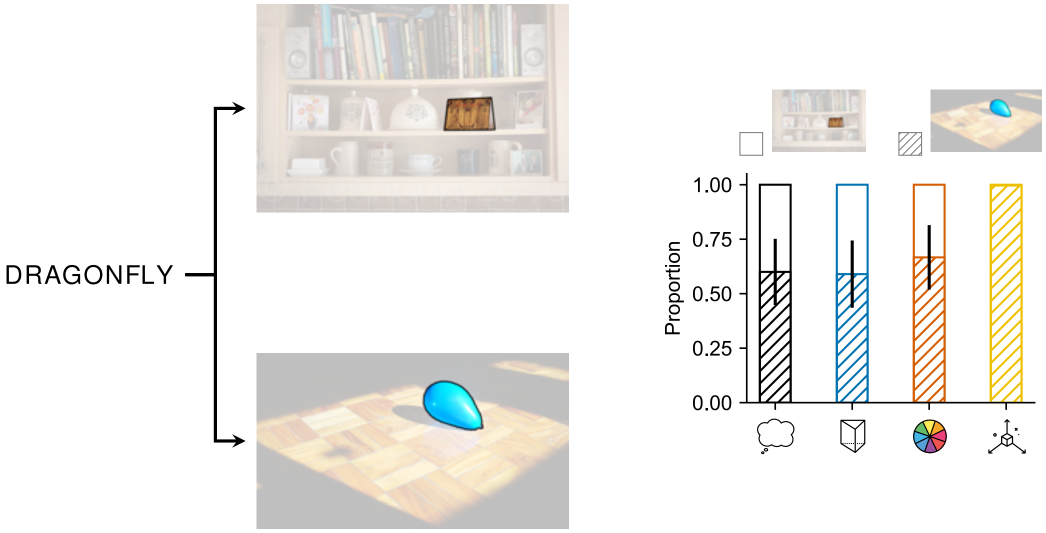

Figure S89: For the item (dragonfly→[card/balloon], left panel) in Study 2, the proportion of participant choosing one of the two options in the PRETENSE, SHAPE, and COLOR conditions, as well as representational similarity derived from CLIP embedding space.

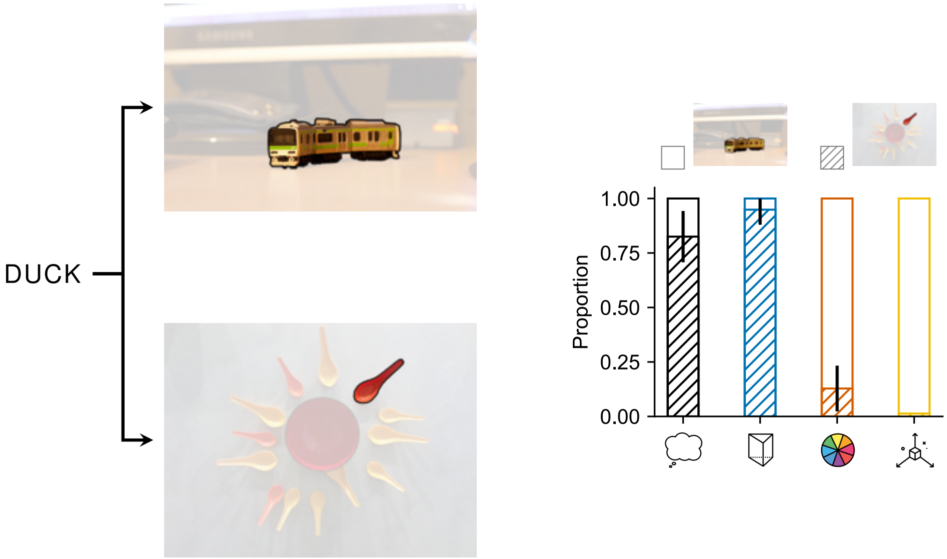

Figure S90: For the item (duck→[train/soup spoon], left panel) in Study 2, the proportion of participant choosing one of the two options in the PRETENSE, SHAPE, and COLOR conditions, as well as representational similarity derived from CLIP embedding space.

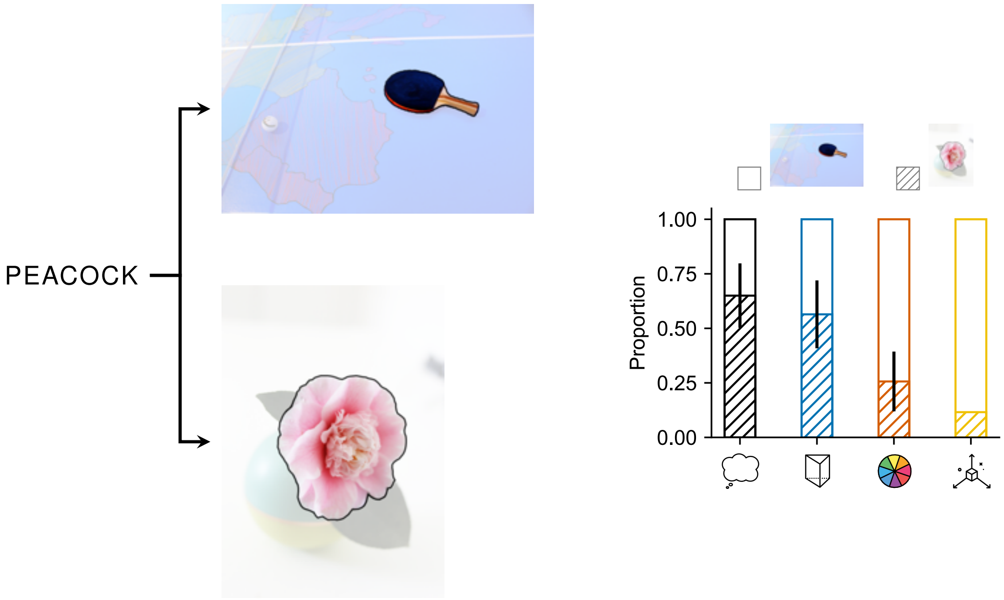

Figure S91: For the item (peacock→[table tennis racket/rose], left panel) in Study 2, the proportion of participant choosing one of the two options in the PRETENSE, SHAPE, and COLOR conditions, as well as representational similarity derived from CLIP embedding space.

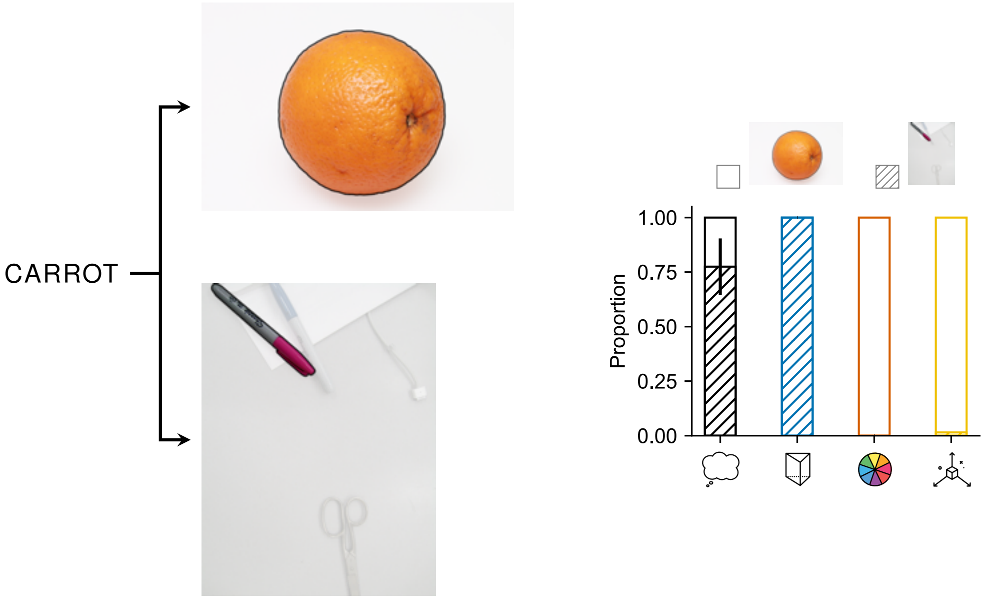

Figure S92: For the item (carrot→[orange/marker], left panel) in Study 2, the proportion of participant choosing one of the two options in the PRETENSE, SHAPE, and COLOR conditions, as well as representational similarity derived from CLIP embedding space.

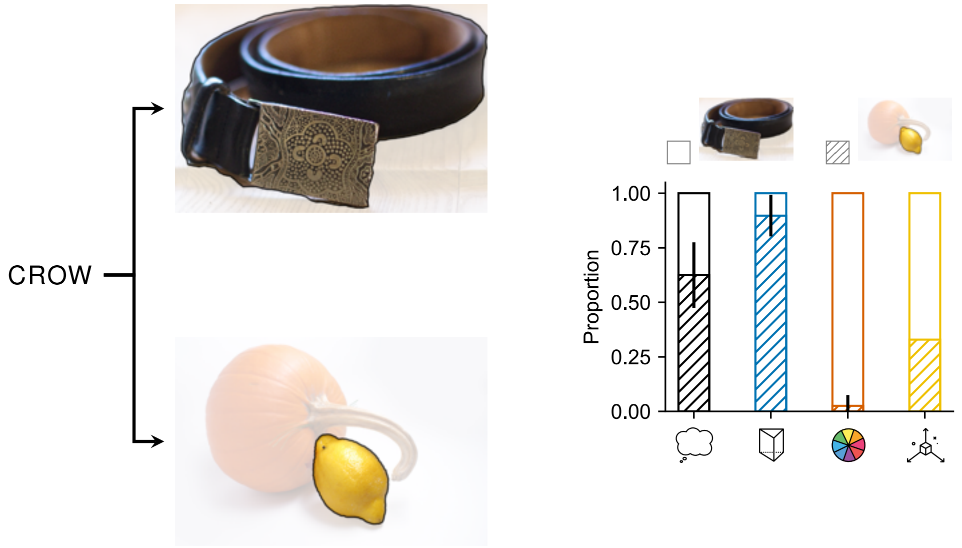

Figure S93: For the item (crow→[belt/lemon], left panel) in Study 2, the proportion of participant choosing one of the two options in the Pretense, Shape, and Color conditions, as well as representational similarity derived from CLIP embedding space.

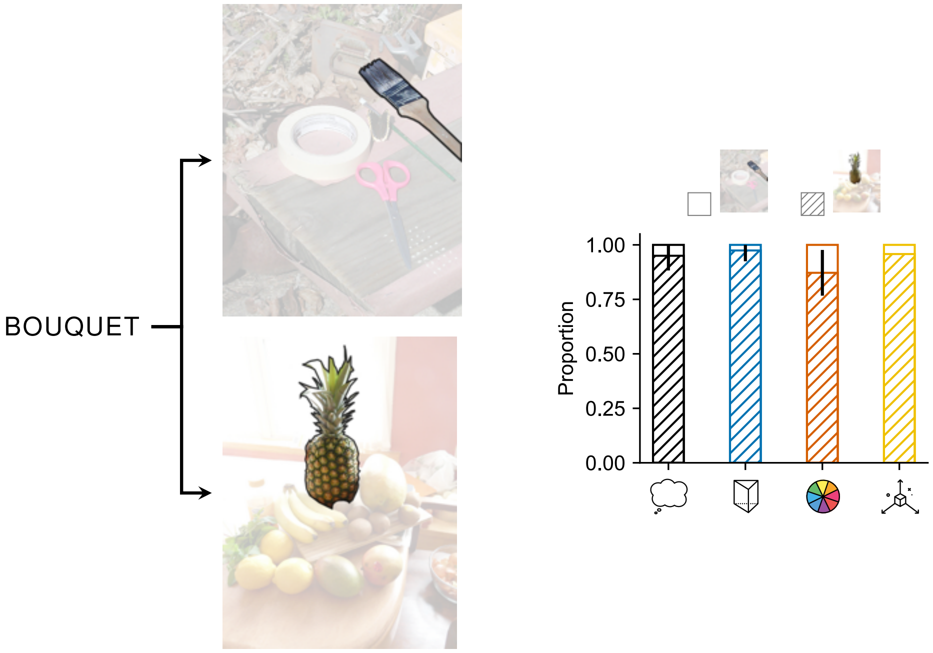

Figure S94: For the item (bouquet→[paint brush/pineapple], left panel) in Study 2, the proportion of participant choosing one of the two options in the Pretense, Shape, and Color conditions, as well as representational similarity derived from CLIP embedding space.

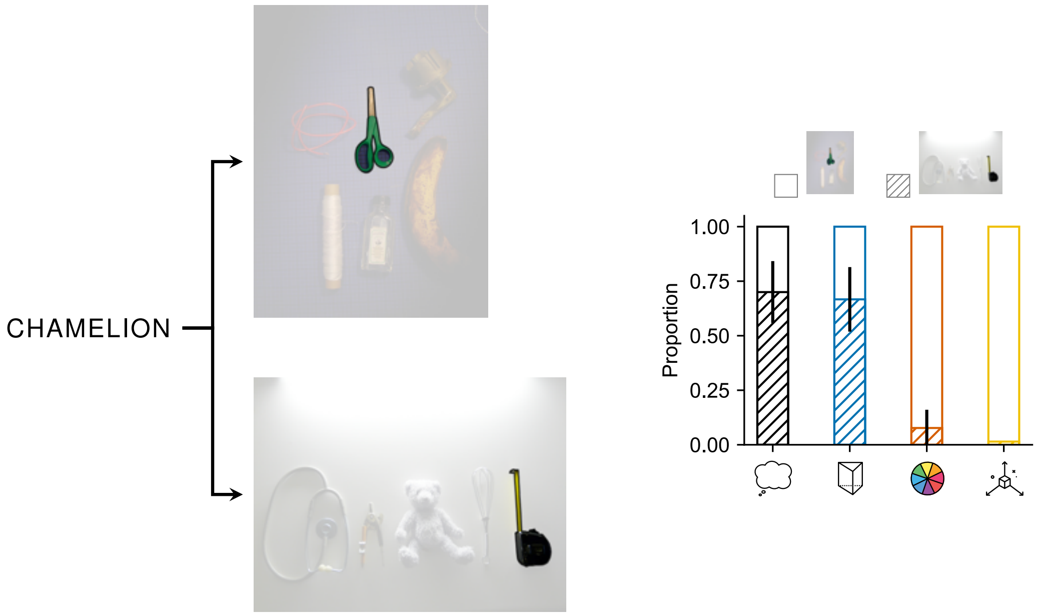

Figure S95: For the item (chamelion→[scissors/tape measure], left panel) in Study 2, the proportion of participant choosing one of the two options in the PRETENSE, SHAPE, and COLOR conditions, as well as representational similarity derived from CLIP embedding space.

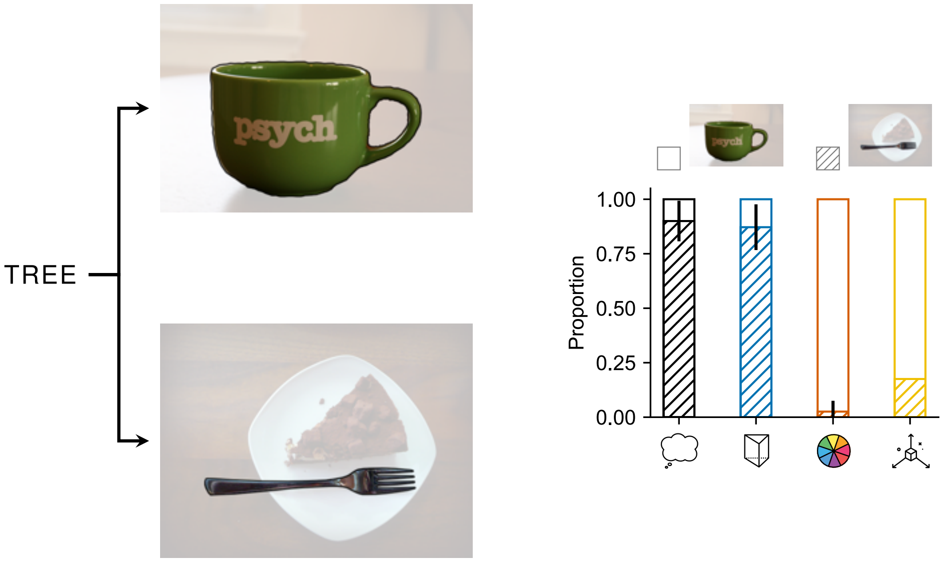

Figure S96: For the item (tree→[mug/fork], left panel) in Study 2, the proportion of participant choosing one of the two options in the PRETENSE, SHAPE, and COLOR conditions, as well as representational similarity derived from CLIP embedding space.

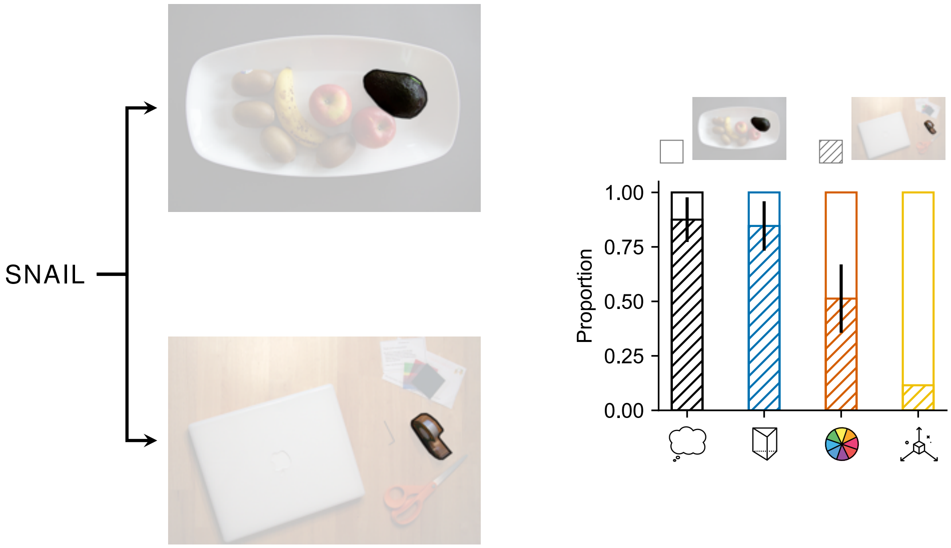

Figure S97: For the item (snail→[avocado/tape], left panel) in Study 2, the proportion of participant choosing one of the two options in the Pretense, Shape, and Color conditions, as well as representational similarity derived from CLIP embedding space.

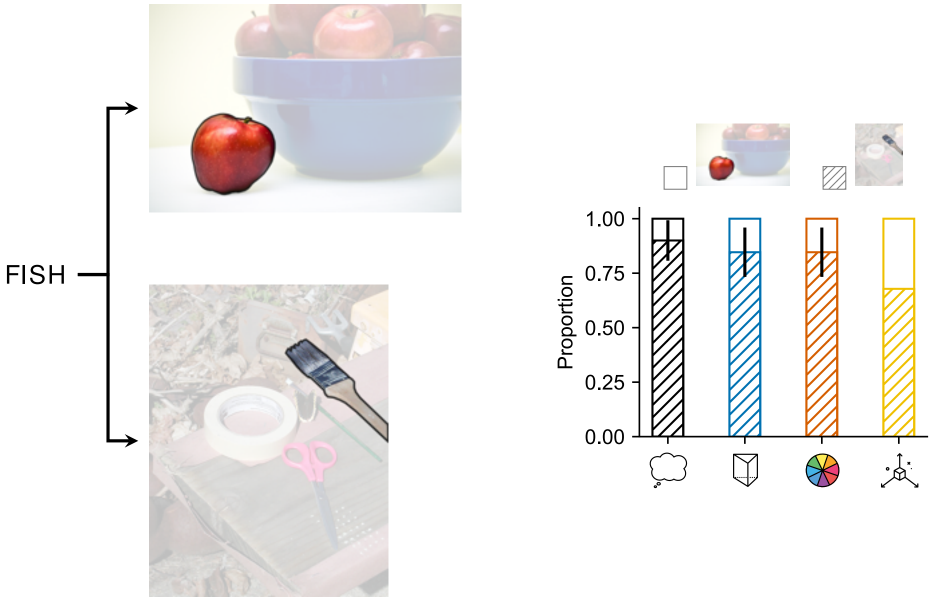

Figure S98: For the item (fish→[apple/paint brush], left panel) in Study 2, the proportion of participant choosing one of the two options in the Pretense, Shape, and Color conditions, as well as representational similarity derived from CLIP embedding space.

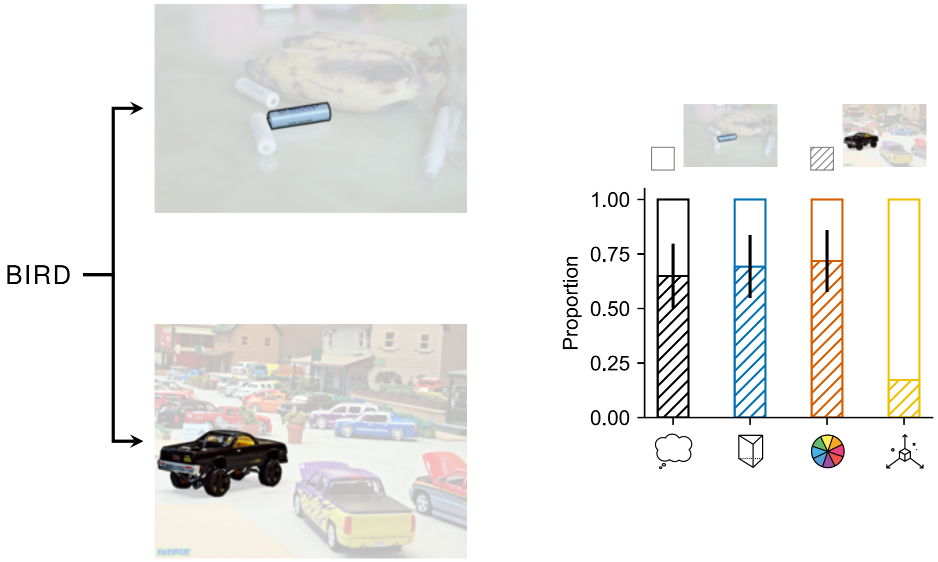

Figure S99: For the item (bird→[battery/car], left panel) in Study 2, the proportion of participant choosing one of the two options in the PRETENSE, SHAPE, and COLOR conditions, as well as representational similarity derived from CLIP embedding space.

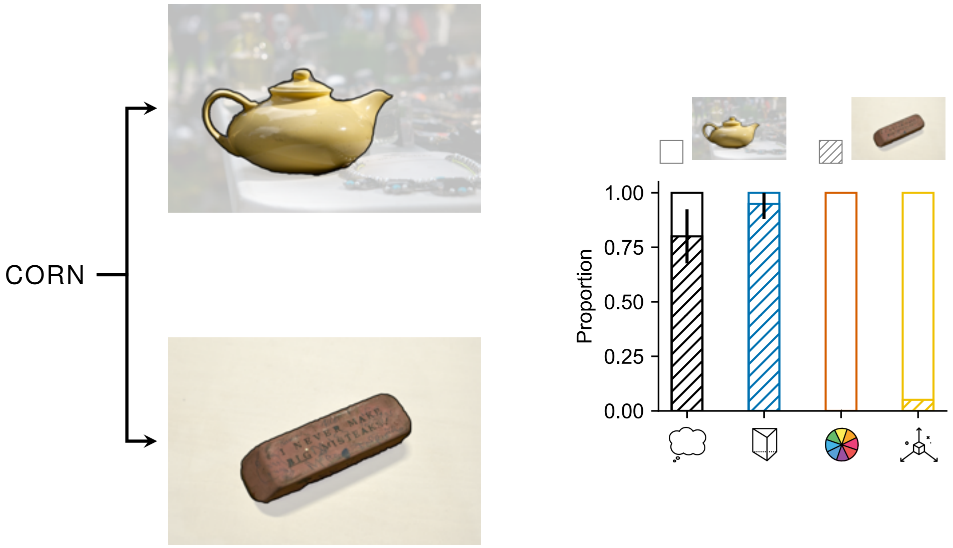

Figure S100: For the item (corn→[teapot/eraser], left panel) in Study 2, the proportion of participant choosing one of the two options in the PRETENSE, SHAPE, and COLOR conditions, as well as representational similarity derived from CLIP embedding space.

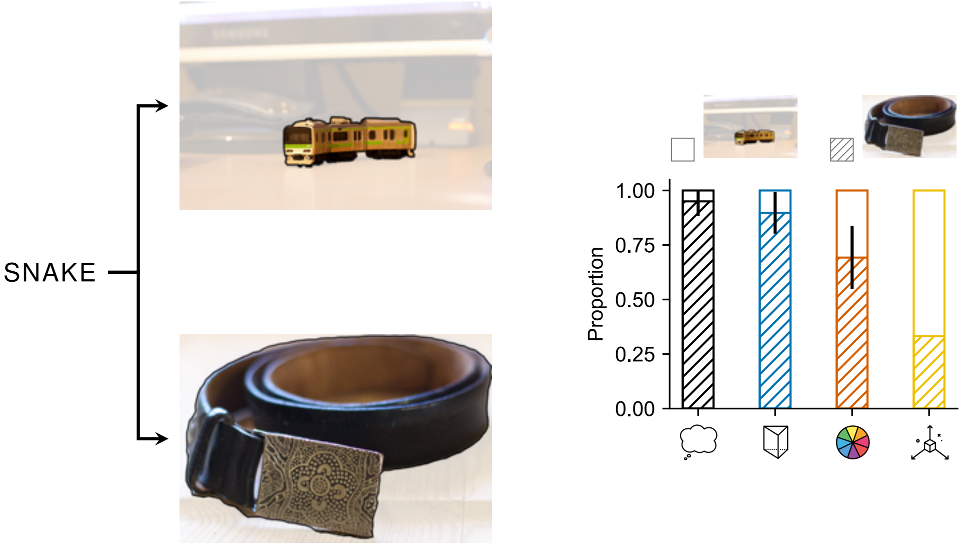

Figure S101: For the item (snake→[train/belt], left panel) in Study 2, the proportion of participant choosing one of the two options in the PRETENSE, SHAPE, and COLOR conditions, as well as representational similarity derived from CLIP embedding space.

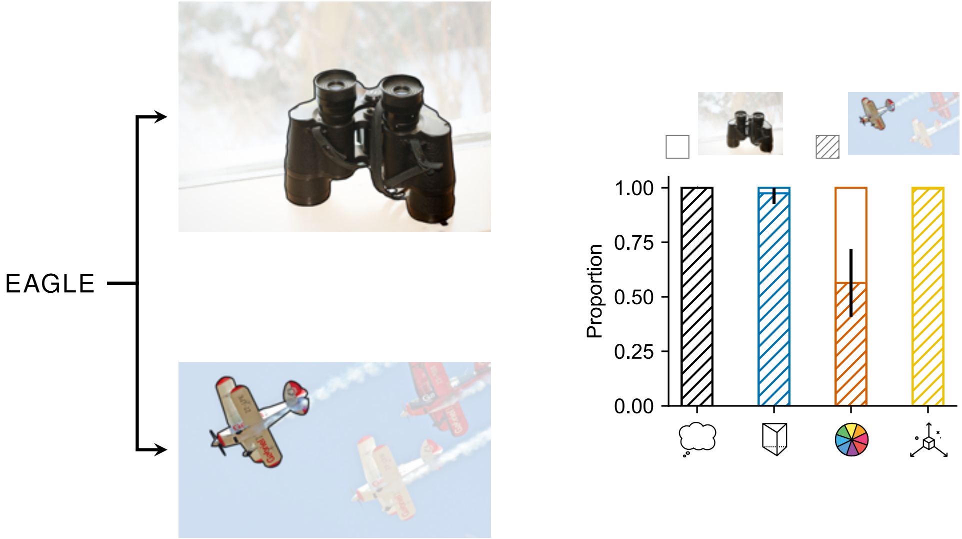

Figure S102: For the item (eagle→[binoculars/airplane], left panel) in Study 2, the proportion of participant choosing one of the two options in the PRETENSE, SHAPE, and COLOR conditions, as well as representational similarity derived from CLIP embedding space.

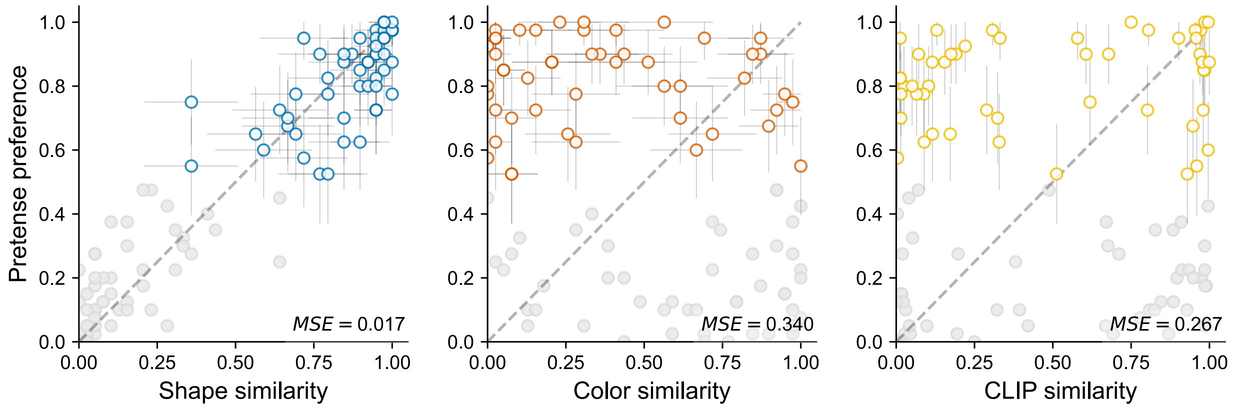

Figure S103: Correlation between pretense preferences and participant-based similarity measures of shape, color, as well as representational similarity derived from CLIP for items in Study 2. The dependent measures (choice proportion) for the two given options of each item are inherently center-symmetrical. The dis-preferred options were plotted in light gray dots, while the preferred pretend options were plotted in blue, red, and yellow for similarity preferences of shape judgment, color judgment, and CLIP embedding space. Error bars indicate 95% confidence intervals with normal approximation.

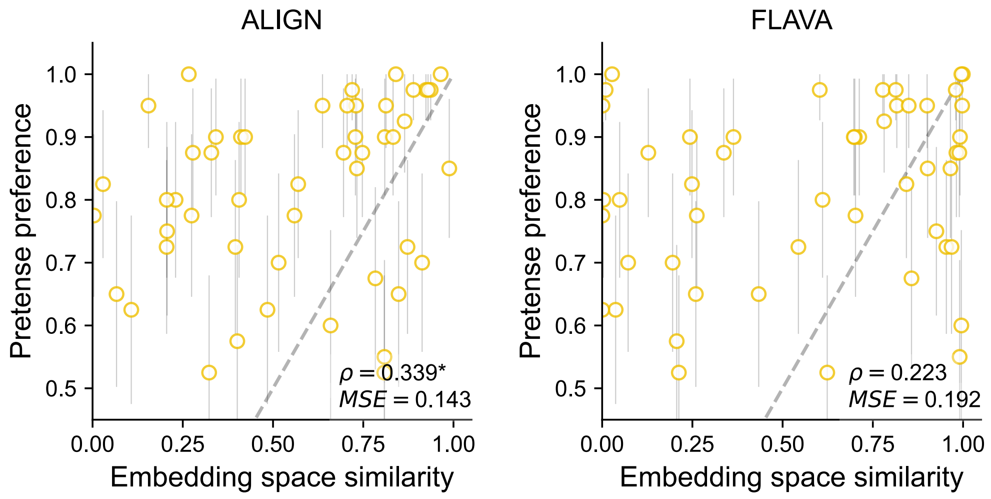

Figure S104: Correlation between pretense preferences and representational similarity derived from ALIGN and FLAVA. The dependent measures (choice proportion) for the two given options of each item are inherently center-symmetrical. Spearman correlation (ρ) across items is based on the strength of preference for the preferred pretend options. Error bars indicate 95% confidence intervals with normal approximation.

D Supplemental Information for Study 3

Figure S105-S154 show the experimental materials used in the free-form generation task together with the frequency distribution of the pretend objects that participants come up with in the study. For each figure, the left panel visualize the stimulus, while the right panel lists the free-form generated options sorted by their relative frequency among the total responses. The frequency count of each option is annotated as superscript.

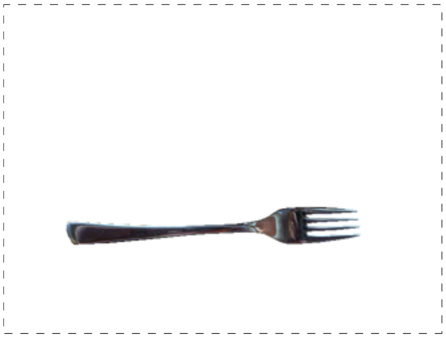

spoon$^{(5)}$, comb$^{(5)}$, spear$^{(3)}$, trident$^{(3)}$, knife$^{(3)}$, rake$^{(2)}$, sword$^{(2)}$, rocket ship$^{(1)}$, metal detector$^{(1)}$, skewer$^{(1)}$, teeth$^{(1)}$, hair pick$^{(1)}$, pirates plank$^{(1)}$, space ship$^{(1)}$, level$^{(1)}$, pool noodle$^{(1)}$, tree$^{(1)}$, spaceship$^{(1)}$, scalpel$^{(1)}$, person$^{(1)}$, battering ram$^{(1)}$, olive branch$^{(1)}$, hair brush$^{(1)}$, miss$^{(1)}$

Figure S105: Participants's free-form generation of pretend objects for the item fork. Frequency count of the term for a pretend option is annotated as superscript.

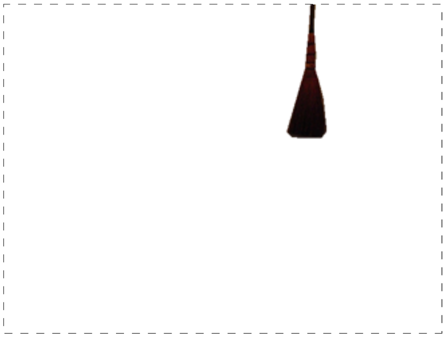

horse$^{(4)}$, dress$^{(3)}$, mop$^{(2)}$, guitar$^{(2)}$, dance partner$^{(2)}$, very small aeronautic vessel$^{(1)}$, vacuum$^{(1)}$, witch hat$^{(1)}$, wall deco$^{(1)}$, ride$^{(1)}$, rocket$^{(1)}$, paintbrush$^{(1)}$, shoe$^{(1)}$, scarecrow$^{(1)}$, hair$^{(1)}$, lacrosse racket$^{(1)}$, long dress$^{(1)}$, spoon$^{(1)}$, tree$^{(1)}$, limbo bar$^{(1)}$, person$^{(1)}$, witches broom$^{(1)}$, tail$^{(1)}$, baseball bat$^{(1)}$, paint brush$^{(1)}$, power washer$^{(1)}$, brush$^{(1)}$, shotgun or a guitar$^{(1)}$, bike$^{(1)}$, jackhammer$^{(1)}$, trumpet$^{(1)}$, funnel$^{(1)}$

Figure S106: Participants's free-form generation of pretend objects for the item broom. Frequency count of the term for a pretend option is annotated as superscript.

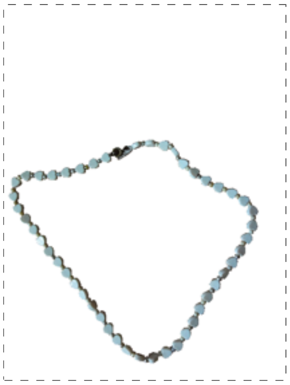

rope$^{(7)}$, bracelet$^{(3)}$, handcuffs$^{(3)}$, snake$^{(3)}$, lasso$^{(2)}$, torture device...$^{(1)}$, restraints$^{(1)}$, braclet$^{(1)}$, collar$^{(1)}$, mouth$^{(1)}$, earrings$^{(1)}$, ants$^{(1)}$, chain$^{(1)}$, tooth$^{(1)}$, door chain$^{(1)}$, curtain tie$^{(1)}$, yarn$^{(1)}$, candy$^{(1)}$, fence seen from above$^{(1)}$, map$^{(1)}$, pearl necklace$^{(1)}$, particle accelerator$^{(1)}$, nobel prize$^{(1)}$, hair tie$^{(1)}$, hula hoop$^{(1)}$, licorice$^{(1)}$, bicycle chain$^{(1)}$

Figure S107: Participants's free-form generation of pretend objects for the item necklace. Frequency count of the term for a pretend option is annotated as superscript.

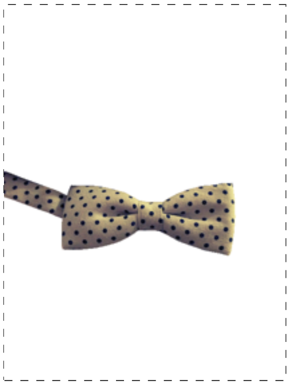

butterfly$^{(3)}$, headband$^{(3)}$, tie$^{(2)}$, necklace$^{(2)}$, hair bow$^{(2)}$, translocation device$^{(1)}$, ribbon$^{(1)}$, handcuffs$^{(1)}$, snail$^{(1)}$, suit$^{(1)}$, barrette$^{(1)}$, barrett$^{(1)}$, fence$^{(1)}$, belt$^{(1)}$, turtle$^{(1)}$, ring$^{(1)}$, cracker$^{(1)}$, gift wrap$^{(1)}$, bungee cord$^{(1)}$, kite ribbon$^{(1)}$, personality changing collar$^{(1)}$, leash$^{(1)}$, animal$^{(1)}$, helicopter blade$^{(1)}$, bracelet$^{(1)}$, tiny pillows$^{(1)}$, dog collar$^{(1)}$, hairbow$^{(1)}$, mustache$^{(1)}$, helicopter$^{(1)}$, top hat$^{(1)}$, listening device$^{(1)}$, tiny cat$^{(1)}$

Figure S108: Participants's free-form generation of pretend objects for the item bow-tie. Frequency count of the term for a pretend option is annotated as superscript.

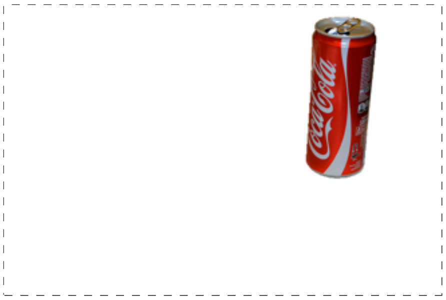

jar$^{(2)}$, cup$^{(2)}$, butterbeer$^{(1)}$, hockey puck$^{(1)}$, tupperware$^{(1)}$, microphone$^{(1)}$, grenade$^{(1)}$, piggy bank$^{(1)}$, phone$^{(1)}$, battery$^{(1)}$, building$^{(1)}$, sentry$^{(1)}$, log$^{(1)}$, telescope$^{(1)}$, roller$^{(1)}$, rolling pin$^{(1)}$, granade$^{(1)}$, ladder$^{(1)}$, model building$^{(1)}$, pillar$^{(1)}$, plastic$^{(1)}$, brick$^{(1)}$, red bull$^{(1)}$, hammer$^{(1)}$, confetti shooter$^{(1)}$, magic potion$^{(1)}$, silo$^{(1)}$, oil canister$^{(1)}$, salt shaker$^{(1)}$, audio speaker$^{(1)}$, tower$^{(1)}$, stove$^{(1)}$, trash collector$^{(1)}$, flash bomb$^{(1)}$, shoe$^{(1)}$, bird$^{(1)}$, submarine$^{(1)}$, storage tank$^{(1)}$

Figure S109: Participants's free-form generation of pretend objects for the item can. Frequency count of the term for a pretend option is annotated as superscript.

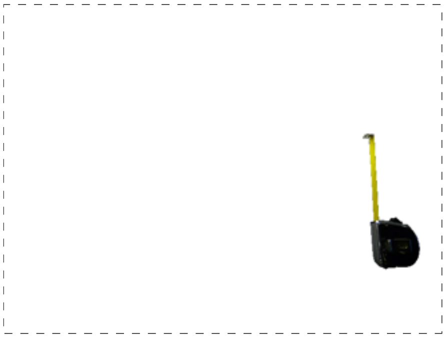

snail$^{(6)}$, ruler$^{(2)}$, giraffe$^{(2)}$, snake$^{(2)}$, sword$^{(2)}$, snake that lives in a shell like a crab$^{(1)}$, stick$^{(1)}$, radio antenna$^{(1)}$, chameleon$^{(1)}$, worm$^{(1)}$, measuring tape$^{(1)}$, door stop$^{(1)}$, duck$^{(1)}$, golf club$^{(1)}$, flag$^{(1)}$, laser$^{(1)}$, ostriche$^{(1)}$, turtle$^{(1)}$, coffee$^{(1)}$, utensil$^{(1)}$, ladder$^{(1)}$, light saber$^{(1)}$, weird animal like a giraffe$^{(1)}$, antenna$^{(1)}$, lazer$^{(1)}$, lightsaber$^{(1)}$, cobra$^{(1)}$, whip$^{(1)}$, level$^{(1)}$, flamingo$^{(1)}$, forklift$^{(1)}$

Figure S110: Participants's free-form generation of pretend objects for the item tape measure. Frequency count of the term for a pretend option is annotated as superscript.

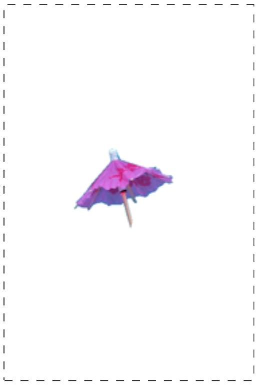

shield$^{(5)}$, hat$^{(3)}$, boat$^{(2)}$, parachute$^{(2)}$, parasol$^{(2)}$, cocktail decoration$^{(1)}$, raincoat$^{(1)}$, cover$^{(1)}$, spoon$^{(1)}$, hair accessory$^{(1)}$, flower$^{(1)}$, drink$^{(1)}$, barbie toy$^{(1)}$, toffee$^{(1)}$, roof$^{(1)}$, firework$^{(1)}$, tire$^{(1)}$, open purse$^{(1)}$, baloon$^{(1)}$, bug hut$^{(1)}$, broken hat$^{(1)}$, toy$^{(1)}$, gazebo$^{(1)}$, fairy roof$^{(1)}$, spinning top$^{(1)}$, awning$^{(1)}$, satellite$^{(1)}$, gun$^{(1)}$, sword$^{(1)}$, space ship$^{(1)}$, ceiling$^{(1)}$

Figure S111: Participants's free-form generation of pretend objects for the item umbrella. Frequency count of the term for a pretend option is annotated as superscript.

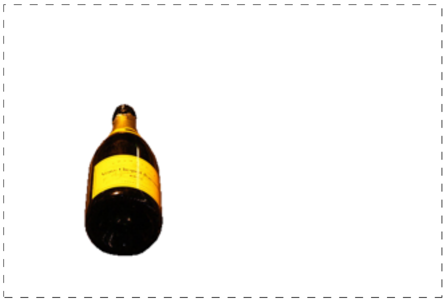

rocket$^{(2)}$, vase$^{(2)}$, rolling pin$^{(2)}$, bat$^{(2)}$, baseball bat$^{(2)}$, weapon$^{(1)}$, vinegar bottle$^{(1)}$, microscope$^{(1)}$, beer$^{(1)}$, roller (workout item)$^{(1)}$, microphone$^{(1)}$, gas$^{(1)}$, wine glass$^{(1)}$, bowling pin$^{(1)}$, cup$^{(1)}$, perfume$^{(1)}$, bmx jump$^{(1)}$, elixir$^{(1)}$, flashlight$^{(1)}$, barrel$^{(1)}$, club$^{(1)}$, spaceship$^{(1)}$, tool$^{(1)}$, fat cat$^{(1)}$, boat horn$^{(1)}$, magic potion$^{(1)}$, maraca$^{(1)}$, pool cue$^{(1)}$, medicine vial$^{(1)}$, juggling club$^{(1)}$, submarine$^{(1)}$, aluminum can$^{(1)}$, arm rest$^{(1)}$, cave man's club$^{(1)}$, phone$^{(1)}$

Figure S112: Participants's free-form generation of pretend objects for the item wine bottle. Frequency count of the term for a pretend option is annotated as superscript.

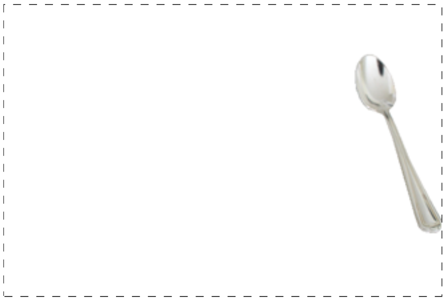

shovel$^{(4)}$, wand$^{(4)}$, fork$^{(2)}$, hammer$^{(2)}$, tool$^{(2)}$, microphone$^{(2)}$, special mirror that lets you enter another dimension$^{(1)}$, magic wand$^{(1)}$, fingernail$^{(1)}$, weapon$^{(1)}$, bottle$^{(1)}$, makeup tool$^{(1)}$, trowel$^{(1)}$, rocket ship$^{(1)}$, rocket$^{(1)}$, pencil$^{(1)}$, drumstick$^{(1)}$, diving board$^{(1)}$, dental mirror$^{(1)}$, slide$^{(1)}$, sword$^{(1)}$, vanity mirror$^{(1)}$, scalpal$^{(1)}$, person$^{(1)}$, relic from long ago$^{(1)}$, paring knife$^{(1)}$, eye depuffer$^{(1)}$, wizards wand$^{(1)}$, paddle$^{(1)}$, mirror$^{(1)}$

Figure S113: Participants's free-form generation of pretend objects for the item spoon. Frequency count of the term for a pretend option is annotated as superscript.

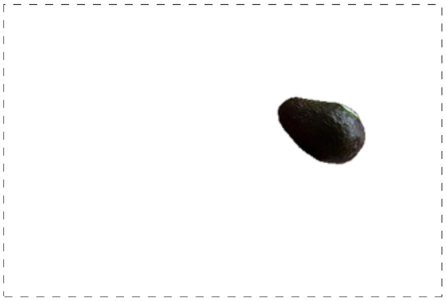

rock$^{(7)}$, seed$^{(2)}$, hand grenade$^{(2)}$, baseball$^{(1)}$, pepper$^{(1)}$, precious stone$^{(1)}$, earring$^{(1)}$, phone$^{(1)}$, tomato$^{(1)}$, dog toy$^{(1)}$, stone$^{(1)}$, pear$^{(1)}$, moon rock$^{(1)}$, escape pod$^{(1)}$, ball$^{(1)}$, mouse$^{(1)}$, small boulder$^{(1)}$, green egg$^{(1)}$, planet$^{(1)}$, pit$^{(1)}$, baby$^{(1)}$, geode$^{(1)}$, bomb$^{(1)}$, dragon egg$^{(1)}$, alien egg$^{(1)}$, apple$^{(1)}$, foam roller$^{(1)}$, frag grenade$^{(1)}$, icecube$^{(1)}$, cupcake$^{(1)}$, space egg$^{(1)}$, distant planet$^{(1)}$

Figure S114: Participants's free-form generation of pretend objects for the item avocado. Frequency count of the term for a pretend option is annotated as superscript.

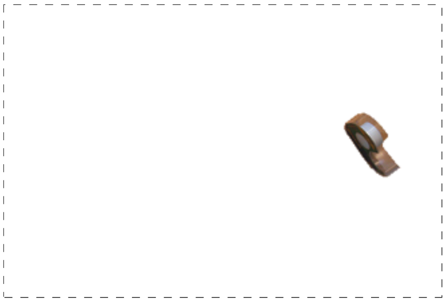

snail$^{(7)}$, glue$^{(4)}$, spyglass$^{(1)}$, kneepad$^{(1)}$, gun$^{(1)}$, staple$^{(1)}$, tool$^{(1)}$, rope$^{(1)}$, paper$^{(1)}$, bee$^{(1)}$, measuring tape$^{(1)}$, fake nail$^{(1)}$, toys$^{(1)}$, cookie mold$^{(1)}$, monster head$^{(1)}$, piece of wood$^{(1)}$, nothing$^{(1)}$, paper clip$^{(1)}$, taco$^{(1)}$, magic lasso$^{(1)}$, fish$^{(1)}$, waterfall$^{(1)}$, smoking pipe$^{(1)}$, lace$^{(1)}$, fly paper$^{(1)}$, measuring device$^{(1)}$, automat$^{(1)}$, one-eyed animal$^{(1)}$, spider-mans webs$^{(1)}$, phone$^{(1)}$, whistle$^{(1)}$

Figure S115: Participants's free-form generation of pretend objects for the item tape. Frequency count of the term for a pretend option is annotated as superscript.

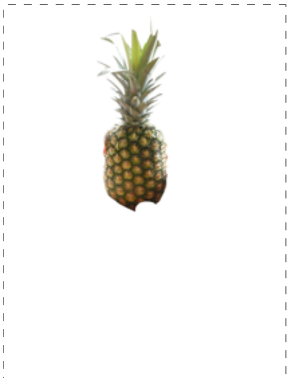

crown$^{(3)}$, head$^{(3)}$, tree$^{(3)}$, decoration$^{(2)}$, hat$^{(2)}$, flower$^{(2)}$, grenade$^{(2)}$, crazy dude who shoots spikes$^{(1)}$, banana$^{(1)}$, bomb$^{(1)}$, ponytail$^{(1)}$, haircut$^{(1)}$, coconut$^{(1)}$, pear$^{(1)}$, volvano$^{(1)}$, cherry bomb$^{(1)}$, head with hair$^{(1)}$, latern$^{(1)}$, dragon$^{(1)}$, women with an exotic hat$^{(1)}$, alien creature$^{(1)}$, person$^{(1)}$, wig$^{(1)}$, palm tree$^{(1)}$, woman wearing a hat$^{(1)}$, insect$^{(1)}$, melon$^{(1)}$, baby$^{(1)}$, wine bottle$^{(1)}$, queen's crown$^{(1)}$

Figure S116: Participants's free-form generation of pretend objects for the item pineapple. Frequency count of the term for a pretend option is annotated as superscript.

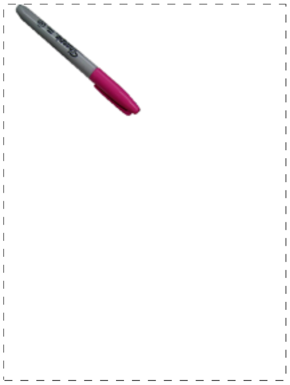

pen$^{(3)}$, magic wand$^{(3)}$, pencil$^{(3)}$, knife$^{(2)}$, laser pointer$^{(2)}$, wand$^{(2)}$, ruler$^{(2)}$, log$^{(2)}$, missile$^{(2)}$, container of smelling salts$^{(1)}$, lipstick$^{(1)}$, eyebrow$^{(1)}$, spear$^{(1)}$, rocket ship$^{(1)}$, gun barrel pointed at sky$^{(1)}$, makeup$^{(1)}$, tree$^{(1)}$, spy camera$^{(1)}$, stylus$^{(1)}$, stick$^{(1)}$, arrow$^{(1)}$, spoon$^{(1)}$, rocket$^{(1)}$, microphone$^{(1)}$, pointer$^{(1)}$, wolverine claw$^{(1)}$, chalk$^{(1)}$, nail$^{(1)}$

Figure S117: Participants's free-form generation of pretend objects for the item marker. Frequency count of the term for a pretend option is annotated as superscript.

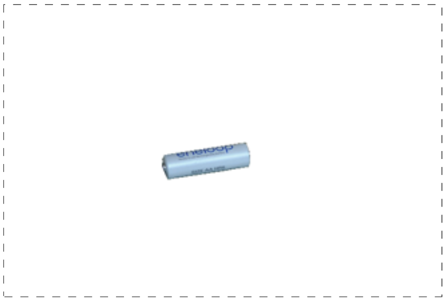

candy$^{(3)}$, log$^{(3)}$, dynamite$^{(2)}$, pill$^{(2)}$, rolling toy$^{(1)}$, roller$^{(1)}$, secret document storage$^{(1)}$, electricity$^{(1)}$, stair$^{(1)}$, gold$^{(1)}$, snack$^{(1)}$, charger$^{(1)}$, chapstick$^{(1)}$, sharpener$^{(1)}$, building block$^{(1)}$, flashlight$^{(1)}$, paper towels$^{(1)}$, robot$^{(1)}$, spaceship engine$^{(1)}$, cargo$^{(1)}$, stapler$^{(1)}$, magic bean$^{(1)}$, c4 charge$^{(1)}$, bomb$^{(1)}$, tootsie roll$^{(1)}$, jewel$^{(1)}$, vape$^{(1)}$, rolling pin$^{(1)}$, plutonium$^{(1)}$, can$^{(1)}$, futon$^{(1)}$, capsule$^{(1)}$, cartridge$^{(1)}$, papyrus$^{(1)}$

Figure S118: Participants's free-form generation of pretend objects for the item battery. Frequency count of the term for a pretend option is annotated as superscript.

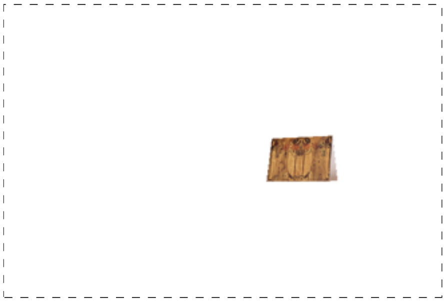

tent$^{(7)}$, door$^{(3)}$, secret message$^{(2)}$, letter$^{(2)}$, wood$^{(2)}$, purse$^{(2)}$, the packaging from a container of takeout$^{(1)}$, picture$^{(1)}$, tarantula$^{(1)}$, hat$^{(1)}$, visor$^{(1)}$, card$^{(1)}$, house$^{(1)}$, wall$^{(1)}$, placemat$^{(1)}$, hightech tablet$^{(1)}$, fence$^{(1)}$, id badge$^{(1)}$, tunnel$^{(1)}$, tiny tent$^{(1)}$, book$^{(1)}$, ticket$^{(1)}$, wallet$^{(1)}$, shuriken$^{(1)}$, spellbook$^{(1)}$, art$^{(1)}$, news paper$^{(1)}$, church facade$^{(1)}$

Figure S119: Participants's free-form generation of pretend objects for the item card. Frequency count of the term for a pretend option is annotated as superscript.

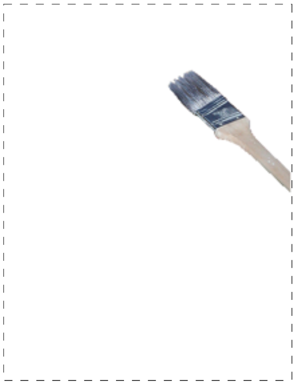

wand$^{(2)}$, paint roller$^{(2)}$, sword$^{(2)}$, makeup brush$^{(2)}$, broom$^{(2)}$, duster$^{(2)}$, toe tickler$^{(1)}$, hair brush$^{(1)}$, powder brush$^{(1)}$, stick person with crazy hair$^{(1)}$, corner$^{(1)}$, cleaning brush$^{(1)}$, belt$^{(1)}$, spounge$^{(1)}$, diving board$^{(1)}$, gun barrel$^{(1)}$, toothbrush$^{(1)}$, kindling$^{(1)}$, microphone$^{(1)}$, grass$^{(1)}$, massager$^{(1)}$, paddle$^{(1)}$, ice scraper for windows$^{(1)}$, tool$^{(1)}$, dog paw$^{(1)}$, plant$^{(1)}$, wing$^{(1)}$, peacock tail$^{(1)}$, canvas$^{(1)}$, beard trimmer$^{(1)}$, fork$^{(1)}$, magic wand$^{(1)}$, birds tail$^{(1)}$, agricultural shovel$^{(1)}$

Figure S120: Participants's free-form generation of pretend objects for the item paint brush. Frequency count of the term for a pretend option is annotated as superscript.

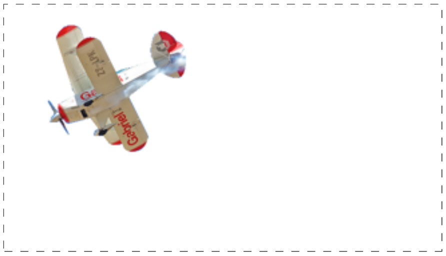

bird$^{(5)}$, spaceship$^{(3)}$, blimp$^{(2)}$, rocket$^{(2)}$, fan$^{(2)}$, toy$^{(2)}$, space ship$^{(2)}$, mechanical bird$^{(1)}$, spy robot$^{(1)}$, tree ornament$^{(1)}$, cardboard$^{(1)}$, helicopter$^{(1)}$, child's toy$^{(1)}$, eagle$^{(1)}$, kite$^{(1)}$, castle$^{(1)}$, alien$^{(1)}$, nothing other than plane$^{(1)}$, sandwich$^{(1)}$, ball$^{(1)}$, submarine$^{(1)}$, dart$^{(1)}$, big fan$^{(1)}$, needle$^{(1)}$, carnival ride$^{(1)}$, drone$^{(1)}$, life raft$^{(1)}$, dragon$^{(1)}$, ufo$^{(1)}$

Figure S121: Participants's free-form generation of pretend objects for the item airplane. Frequency count of the term for a pretend option is annotated as superscript.

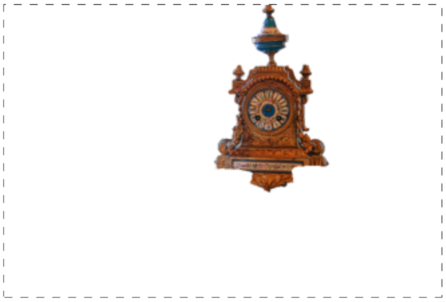

time machine$^{(2)}$, watch$^{(2)}$, ancient computer$^{(1)}$, headpiece$^{(1)}$, cogsworth from beauty and the beast$^{(1)}$, emblem$^{(1)}$, tv$^{(1)}$, lamp$^{(1)}$, stool$^{(1)}$, doll house$^{(1)}$, buddhist monk$^{(1)}$, safe$^{(1)}$, robot$^{(1)}$, treasure$^{(1)}$, house$^{(1)}$, sundial$^{(1)}$, temple$^{(1)}$, hiking$^{(1)}$, bomb$^{(1)}$, strange alien artifact$^{(1)}$, ancient god$^{(1)}$, furniture$^{(1)}$, grand father clock$^{(1)}$, secret code$^{(1)}$, lock for a secret door$^{(1)}$, statue$^{(1)}$, fairy castle$^{(1)}$, birdfeeder$^{(1)}$, picture$^{(1)}$, time travel device$^{(1)}$, energy device$^{(1)}$, pickaxe$^{(1)}$, wall art$^{(1)}$, universal docking station for tiny interplanetary travelers$^{(1)}$, figurehead$^{(1)}$, alien face$^{(1)}$, ancient building$^{(1)}$, church facade$^{(1)}$

Figure S122: Participants's free-form generation of pretend objects for the item clock. Frequency count of the term for a pretend option is annotated as superscript.

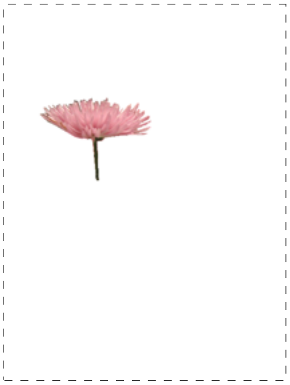

umbrella$^{(7)}$, rose$^{(3)}$, fan$^{(2)}$, duster$^{(2)}$, parachute$^{(2)}$, huge tree$^{(1)}$, parachute for ants$^{(1)}$, fairy$^{(1)}$, sunflower$^{(1)}$, hat$^{(1)}$, creature$^{(1)}$, firework$^{(1)}$, wheel$^{(1)}$, ballerina$^{(1)}$, small tree$^{(1)}$, pompom$^{(1)}$, umbrella turned upward$^{(1)}$, planet$^{(1)}$, strawberry$^{(1)}$, ingredient to a potion$^{(1)}$, food$^{(1)}$, broom$^{(1)}$, feather$^{(1)}$, daisy$^{(1)}$, pen$^{(1)}$, paint brush$^{(1)}$, lilypad$^{(1)}$, lolipop$^{(1)}$, parasol$^{(1)}$

Figure S123: Participants's free-form generation of pretend objects for the item carnation. Frequency count of the term for a pretend option is annotated as superscript.

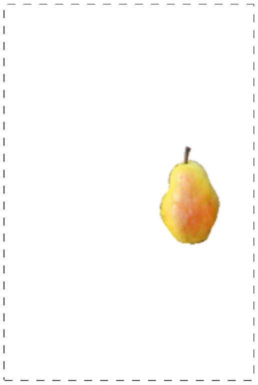

apple$^{(4)}$, grenade$^{(4)}$, ball$^{(3)}$, avocado$^{(2)}$, guitar$^{(2)}$, rock$^{(2)}$, beautiful lady$^{(1)}$, bush$^{(1)}$, food$^{(1)}$, violin$^{(1)}$, bananas$^{(1)}$, make up sponge$^{(1)}$, green pepper$^{(1)}$, barrel$^{(1)}$, bomb$^{(1)}$, tennis ball$^{(1)}$, hedgehog$^{(1)}$, soul$^{(1)}$, monster egg$^{(1)}$, weird apple$^{(1)}$, mango$^{(1)}$, hot pepper$^{(1)}$, tooth$^{(1)}$, paper holder$^{(1)}$, duckling$^{(1)}$, grape$^{(1)}$, stress ball$^{(1)}$, bug$^{(1)}$, baby chick$^{(1)}$

Figure S124: Participants's free-form generation of pretend objects for the item pear. Frequency count of the term for a pretend option is annotated as superscript.

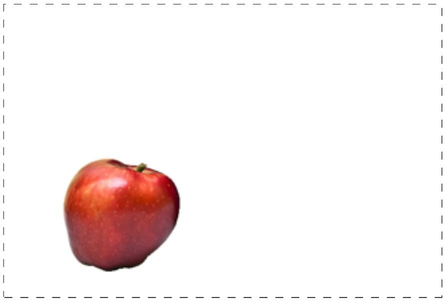

ball$^{(7)}$, rock$^{(4)}$, pear$^{(2)}$, heart$^{(2)}$, ruby$^{(2)}$, football$^{(1)}$, hot coals$^{(1)}$, button$^{(1)}$, eye$^{(1)}$, orange$^{(1)}$, mango$^{(1)}$, snow ball$^{(1)}$, apple$^{(1)}$, doorstop$^{(1)}$, monster egg$^{(1)}$, fake fruit$^{(1)}$, planet$^{(1)}$, women smiling$^{(1)}$, pet$^{(1)}$, bowling ball$^{(1)}$, gem$^{(1)}$, ceramic molding$^{(1)}$, grapefruit$^{(1)}$, paperweight$^{(1)}$, small galaxy that we are the gods of$^{(1)}$, baseball$^{(1)}$, cupcake$^{(1)}$, asteroid$^{(1)}$

Figure S125: Participants's free-form generation of pretend objects for the item apple. Frequency count of the term for a pretend option is annotated as superscript.

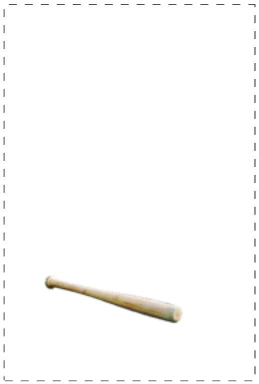

cane$^{(4)}$, club$^{(2)}$, bottle$^{(2)}$, sword$^{(2)}$, huge dildo$^{(1)}$, boat oar$^{(1)}$, baton$^{(1)}$, toothpick$^{(1)}$, horse$^{(1)}$, ledge$^{(1)}$, tennis racket$^{(1)}$, oar$^{(1)}$, pencil$^{(1)}$, log$^{(1)}$, gun barrel$^{(1)}$, pool stick$^{(1)}$, pen$^{(1)}$, door prop$^{(1)}$, lightsaber$^{(1)}$, qtip$^{(1)}$, tree trunk$^{(1)}$, toy$^{(1)}$, cigarette$^{(1)}$, rat$^{(1)}$, fence post$^{(1)}$, airplane that lost its wings$^{(1)}$, stick$^{(1)}$, car jack lever$^{(1)}$, rolling pin$^{(1)}$, bone$^{(1)}$, golf club$^{(1)}$, mystical sword$^{(1)}$, plane$^{(1)}$, roller$^{(1)}$

Figure S126: Participants's free-form generation of pretend objects for the item baseball bat. Frequency count of the term for a pretend option is annotated as superscript.

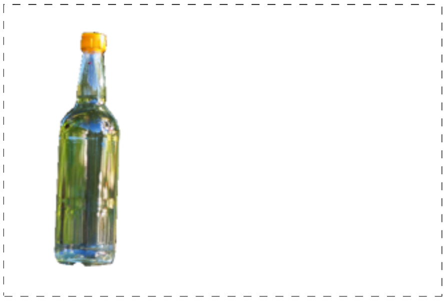

rocket$^{(3)}$, telescope$^{(3)}$, rolling pin$^{(2)}$, baseball bat$^{(2)}$, broken$^{(1)}$, can$^{(1)}$, spyglass$^{(1)}$, wine$^{(1)}$, pole$^{(1)}$, bus$^{(1)}$, glass$^{(1)}$, garden statue$^{(1)}$, latern$^{(1)}$, bird feeder$^{(1)}$, art$^{(1)}$, support$^{(1)}$, microphone$^{(1)}$, magnifying glass$^{(1)}$, mirror$^{(1)}$, anicent message$^{(1)}$, tool$^{(1)}$, glass fish tank$^{(1)}$, nightstick$^{(1)}$, drumstick$^{(1)}$, mountain$^{(1)}$, bat$^{(1)}$, cone$^{(1)}$, person$^{(1)}$, spy glass$^{(1)}$, vessel$^{(1)}$, vase$^{(1)}$, alive$^{(1)}$, phone$^{(1)}$, green lake$^{(1)}$

Figure S127: Participants's free-form generation of pretend objects for the item bottle. Frequency count of the term for a pretend option is annotated as superscript.

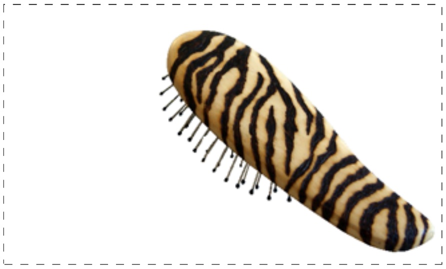

caterpillar$^{(5)}$, centipede$^{(4)}$, comb$^{(3)}$, slipper$^{(3)}$, microphone$^{(3)}$, catepillar$^{(2)}$, insect$^{(2)}$, porcupine$^{(2)}$, cat$^{(1)}$, lint brush$^{(1)}$, alien creature$^{(1)}$, belly$^{(1)}$, snail$^{(1)}$, starfish$^{(1)}$, caterpiller$^{(1)}$, car$^{(1)}$, letter holder$^{(1)}$, spider$^{(1)}$, zebra paw$^{(1)}$, massager$^{(1)}$, knife$^{(1)}$, millipede$^{(1)}$, dogbrush$^{(1)}$, hedgehog tiger$^{(1)}$

Figure S128: Participants's free-form generation of pretend objects for the item hairbrush. Frequency count of the term for a pretend option is annotated as superscript.

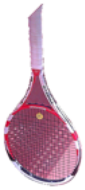

fly swatter$^{(5)}$, guitar$^{(3)}$, baseball bat$^{(2)}$, sword$^{(2)}$, paddle$^{(2)}$, cheese grater$^{(1)}$, table tennis racket$^{(1)}$, cane$^{(1)}$, net$^{(1)}$, gum$^{(1)}$, sieve$^{(1)}$, snow shoe$^{(1)}$, building$^{(1)}$, drop of water$^{(1)}$, flyswatter$^{(1)}$, mask$^{(1)}$, bubble wand$^{(1)}$, plate$^{(1)}$, play ground$^{(1)}$, spatula$^{(1)}$, plant$^{(1)}$, stump$^{(1)}$, face shield$^{(1)}$, fan$^{(1)}$, mirror$^{(1)}$, tire flap$^{(1)}$, banjo$^{(1)}$, baseball glove$^{(1)}$, broom$^{(1)}$, frying pan$^{(1)}$, perfume bottle$^{(1)}$

Figure S129: Participants's free-form generation of pretend objects for the item tennis racket. Frequency count of the term for a pretend option is annotated as superscript.

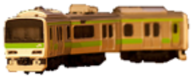

worm$^{(3)}$, bus$^{(3)}$, snake$^{(3)}$, caterpillar$^{(3)}$, truck$^{(2)}$, really long car$^{(1)}$, streetcar$^{(1)}$, radio$^{(1)}$, car$^{(1)}$, rollerskate$^{(1)}$, dragon$^{(1)}$, plane$^{(1)}$, hospital$^{(1)}$, tunnel$^{(1)}$, robot$^{(1)}$, big caterpillar$^{(1)}$, fence$^{(1)}$, toy$^{(1)}$, row housing$^{(1)}$, trolley$^{(1)}$, mechanical caterpillar$^{(1)}$, space train$^{(1)}$, mobile home$^{(1)}$, centipede$^{(1)}$, automobile$^{(1)}$, space vehicle$^{(1)}$, seat$^{(1)}$, giant caterpillar$^{(1)}$, subway$^{(1)}$, space ship$^{(1)}$, residential$^{(1)}$

Figure S130: Participants's free-form generation of pretend objects for the item train. Frequency count of the term for a pretend option is annotated as superscript.

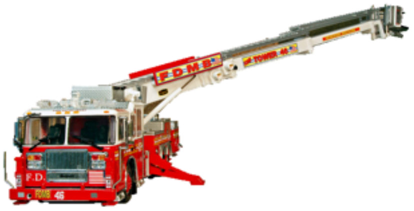

dinosaur$^{(3)}$, crane$^{(3)}$, toy$^{(2)}$, some kind of prehistoric creature with a long trunk$^{(1)}$, garbage truck$^{(1)}$, vacuum cleaner$^{(1)}$, trash tuck$^{(1)}$, telephone pole$^{(1)}$, fridge$^{(1)}$, ambulance$^{(1)}$, construction crane$^{(1)}$, shark$^{(1)}$, ladder$^{(1)}$, toaster$^{(1)}$, machine$^{(1)}$, gun turret$^{(1)}$, backpack$^{(1)}$, phone with antenna$^{(1)}$, house$^{(1)}$, step stool$^{(1)}$, robot$^{(1)}$, brontosaurus$^{(1)}$, animal$^{(1)}$, elevator$^{(1)}$, transformer$^{(1)}$, construction vehicle$^{(1)}$, cherry picker$^{(1)}$, mining equipment$^{(1)}$, alien bug$^{(1)}$, pool inflatable$^{(1)}$, giant dog$^{(1)}$, boat$^{(1)}$, spaceship$^{(1)}$, godzilla$^{(1)}$, drawbridge$^{(1)}$

Figure S131: Participants's free-form generation of pretend objects for the item fire truck. Frequency count of the term for a pretend option is annotated as superscript.

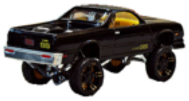

truck$^{(5)}$, spaceship$^{(3)}$, toy$^{(3)}$, flying car$^{(2)}$, tank$^{(2)}$, bomb diffusing robot$^{(1)}$, skateboard$^{(1)}$, taxi$^{(1)}$, jumbotron$^{(1)}$, wagon$^{(1)}$, food delivery cart$^{(1)}$, submarine$^{(1)}$, noting other than car$^{(1)}$, cheetah$^{(1)}$, animal$^{(1)}$, stamp$^{(1)}$, war tank$^{(1)}$, drink transporter$^{(1)}$, bar$^{(1)}$, carriage$^{(1)}$, blackboard eraser$^{(1)}$, ant$^{(1)}$, train$^{(1)}$, bench$^{(1)}$, monster$^{(1)}$, wheelchair$^{(1)}$, kaiju monster$^{(1)}$, go kart$^{(1)}$, cow$^{(1)}$, trailer$^{(1)}$

Figure S132: Participants's free-form generation of pretend objects for the item car. Frequency count of the term for a pretend option is annotated as superscript.

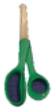

knife$^{(4)}$, sword$^{(3)}$, alligator$^{(2)}$, delicious snack$^{(1)}$, fork$^{(1)}$, bird$^{(1)}$, plant$^{(1)}$, eyes$^{(1)}$, paper$^{(1)}$, teaspoon & tablespoon$^{(1)}$, comb$^{(1)}$, spear$^{(1)}$, car$^{(1)}$, decoration$^{(1)}$, rocket$^{(1)}$, person$^{(1)}$, wieldy knife$^{(1)}$, headphones$^{(1)}$, utensils$^{(1)}$, measuring spoons hooked together$^{(1)}$, insect$^{(1)}$, mickey mouse$^{(1)}$, throwing star$^{(1)}$, fish$^{(1)}$, lizard$^{(1)}$, dart$^{(1)}$, gymnast$^{(1)}$, door knob$^{(1)}$, duck toy$^{(1)}$, tail$^{(1)}$, ornament$^{(1)}$, claws$^{(1)}$, castanets$^{(1)}$, avocado wedges$^{(1)}$

Figure S133: Participants's free-form generation of pretend objects for the item scissors. Frequency count of the term for a pretend option is annotated as superscript.

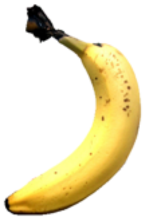

phone$^{(8)}$, boomerang$^{(5)}$, telephone$^{(5)}$, frisbee$^{(2)}$, moon$^{(2)}$, plaintain$^{(1)}$, lime$^{(1)}$, cellphone$^{(1)}$, gun$^{(1)}$, ear ring$^{(1)}$, pear$^{(1)}$, hotdog$^{(1)}$, confetti cannon$^{(1)}$, hook$^{(1)}$, smile$^{(1)}$, clown$^{(1)}$, sex toy$^{(1)}$, slug$^{(1)}$, high heel$^{(1)}$, fungal amalgamation$^{(1)}$, digging tool$^{(1)}$, apple$^{(1)}$, boomerand$^{(1)}$

Figure S134: Participants's free-form generation of pretend objects for the item banana. Frequency count of the term for a pretend option is annotated as superscript.

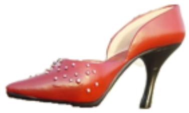

phone$^{(5)}$, weapon$^{(4)}$, hammer$^{(3)}$, tennis shoe$^{(2)}$, boat$^{(2)}$, playground slide$^{(2)}$, magic slipper$^{(1)}$, flats$^{(1)}$, wine stopper$^{(1)}$, glass slipper$^{(1)}$, sesame$^{(1)}$, door stop$^{(1)}$, mountain$^{(1)}$, sledding hill$^{(1)}$, drag racer$^{(1)}$, glove$^{(1)}$, horn$^{(1)}$, hill$^{(1)}$, weird triangle$^{(1)}$, cellphone$^{(1)}$, slipper$^{(1)}$, spike trap (upside down)$^{(1)}$, gun$^{(1)}$, dagger$^{(1)}$, clog$^{(1)}$, eraser$^{(1)}$, ninjas throwing knife$^{(1)}$, cat lying down$^{(1)}$

Figure S135: Participants's free-form generation of pretend objects for the item high heel. Frequency count of the term for a pretend option is annotated as superscript.

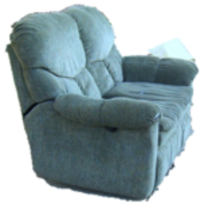

bed$^{(6)}$, chair$^{(4)}$, car$^{(3)}$, boat$^{(3)}$, couch creature that eats people when they sit on it$^{(1)}$, slipper$^{(1)}$, ship$^{(1)}$, monster$^{(1)}$, garbage can$^{(1)}$, fort$^{(1)}$, couch$^{(1)}$, mountain$^{(1)}$, spaceship$^{(1)}$, talking couch$^{(1)}$, pillow$^{(1)}$, people$^{(1)}$, two people hugging$^{(1)}$, nest$^{(1)}$, main base of a fort$^{(1)}$, castle$^{(1)}$, cloud$^{(1)}$, pilot's seat in airplane$^{(1)}$, grumpy coworker$^{(1)}$, armchair$^{(1)}$, sled$^{(1)}$, bear$^{(1)}$, space ship$^{(1)}$, two men sitting$^{(1)}$

Figure S136: Participants's free-form generation of pretend objects for the item studio couch. Frequency count of the term for a pretend option is annotated as superscript.

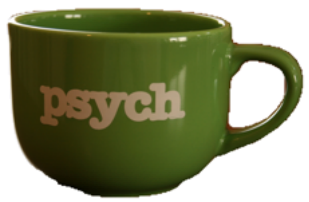

bowl$^{(8)}$, hat$^{(3)}$, cup$^{(3)}$, pool$^{(3)}$, hot tub$^{(2)}$, swimming pool$^{(2)}$, cauldron$^{(1)}$, glass$^{(1)}$, goblet$^{(1)}$, lid$^{(1)}$, mug$^{(1)}$, ornament$^{(1)}$, small bucket$^{(1)}$, soup bowl$^{(1)}$, tub$^{(1)}$, tea kettle$^{(1)}$, mountain for toys to climb$^{(1)}$, crucible$^{(1)}$, flower pot$^{(1)}$, bird's nest$^{(1)}$, dog bowl$^{(1)}$, kippah$^{(1)}$, planter$^{(1)}$, well$^{(1)}$, witch's hole$^{(1)}$

Figure S137: Participants's free-form generation of pretend objects for the item mug. Frequency count of the term for a pretend option is annotated as superscript.

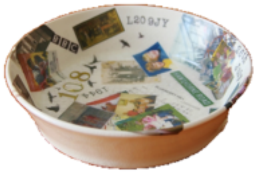

hat$^{(9)}$, plate$^{(4)}$, swimming pool$^{(4)}$, cup$^{(2)}$, boat$^{(2)}$, upside down drum$^{(1)}$, steering wheel$^{(1)}$, pool toy$^{(1)}$, barrel$^{(1)}$, tub$^{(1)}$, bowl$^{(1)}$, chair$^{(1)}$, clock$^{(1)}$, newspaper$^{(1)}$, skate park$^{(1)}$, bathtub$^{(1)}$, stamp$^{(1)}$, sink$^{(1)}$, pot$^{(1)}$, hole$^{(1)}$, pool$^{(1)}$, key container$^{(1)}$, helmet$^{(1)}$, children's pool$^{(1)}$

Figure S138: Participants's free-form generation of pretend objects for the item bowl. Frequency count of the term for a pretend option is annotated as superscript.

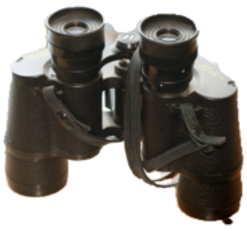

telescope$^{(6)}$, glasses$^{(2)}$, night vision goggles$^{(2)}$, goggles$^{(2)}$, robot$^{(2)}$, mountain$^{(2)}$, teleportation device that transports you to the spot you see$^{(1)}$, spider$^{(1)}$, science tool$^{(1)}$, gun$^{(1)}$, vr$^{(1)}$, smiley face$^{(1)}$, laser pointers$^{(1)}$, baby$^{(1)}$, double microphone$^{(1)}$, tree trunks$^{(1)}$, big glasses$^{(1)}$, r2 3p0$^{(1)}$, butterfly creature$^{(1)}$, telephone$^{(1)}$, camera$^{(1)}$, crab$^{(1)}$, tower$^{(1)}$, teleport machine$^{(1)}$, space station$^{(1)}$, art piece$^{(1)}$, space ships steering wheel$^{(1)}$, device to look into your past$^{(1)}$, rocket$^{(1)}$, laser gun$^{(1)}$

Figure S139: Participants's free-form generation of pretend objects for the item binoculars. Frequency count of the term for a pretend option is annotated as superscript.

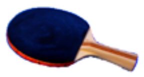

paddle$^{(4)}$, fan$^{(4)}$, tennis racket$^{(2)}$, fly swatter$^{(2)}$, mirror$^{(2)}$, spatula$^{(2)}$, spanking paddle$^{(1)}$, plate$^{(1)}$, tooth$^{(1)}$, sauce pan$^{(1)}$, diving board$^{(1)}$, missile$^{(1)}$, boat paddle$^{(1)}$, skateboard$^{(1)}$, pancake flipper$^{(1)}$, purse$^{(1)}$, forest$^{(1)}$, frying pan$^{(1)}$, hairbrush$^{(1)}$, cutting board$^{(1)}$, rattle$^{(1)}$, defibrillator$^{(1)}$, helicopter$^{(1)}$, pancake turner$^{(1)}$, fighting arena$^{(1)}$, ukulele$^{(1)}$, bat$^{(1)}$, book stand$^{(1)}$, butt scratcher$^{(1)}$, road to the lake$^{(1)}$

Figure S140: Participants's free-form generation of pretend objects for the item table tennis racket. Frequency count of the term for a pretend option is annotated as superscript.

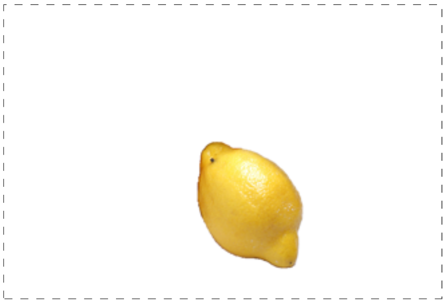

grenade$^{(4)}$, tennis ball$^{(4)}$, ball$^{(3)}$, lime$^{(2)}$, orange$^{(2)}$, bird$^{(2)}$, football$^{(2)}$, huge ball of snot$^{(1)}$, head$^{(1)}$, guava$^{(1)}$, sun$^{(1)}$, pod$^{(1)}$, cork$^{(1)}$, dome$^{(1)}$, slug$^{(1)}$, planet$^{(1)}$, seed$^{(1)}$, nose$^{(1)}$, geode$^{(1)}$, marshmallow peep$^{(1)}$, duck$^{(1)}$, yellow bird$^{(1)}$, chicken chick$^{(1)}$, spinning top$^{(1)}$, watermelon$^{(1)}$, anime characters special move (rasengan - naruto)$^{(1)}$, cupcake$^{(1)}$, pirate trunk$^{(1)}$

Figure S141: Participants's free-form generation of pretend objects for the item lemon. Frequency count of the term for a pretend option is annotated as superscript.

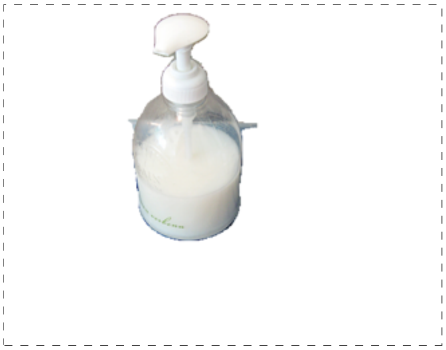

penguin$^{(2)}$, perfume bottle$^{(2)}$, collection$^{(1)}$, barsoap$^{(1)}$, potion bottle$^{(1)}$, sauce container$^{(1)}$, milk carton$^{(1)}$, dinosaur$^{(1)}$, hand sanatizer$^{(1)}$, lotion pump$^{(1)}$, robot$^{(1)}$, shampoo$^{(1)}$, bowl$^{(1)}$, faucet$^{(1)}$, space gun$^{(1)}$, fire extinguisher$^{(1)}$, car$^{(1)}$, small baseball bat$^{(1)}$, gun$^{(1)}$, diving board$^{(1)}$, snowman$^{(1)}$, lava dispenser$^{(1)}$, marshmallow creme dispenser$^{(1)}$, animal$^{(1)}$, poison$^{(1)}$, coffee creamer$^{(1)}$, crystal ball$^{(1)}$, baby duck$^{(1)}$, washing machine$^{(1)}$, milk churn$^{(1)}$, coffee pot with spigot$^{(1)}$, rocket$^{(1)}$, thallium$^{(1)}$, eternal youth elixir$^{(1)}$, lotion$^{(1)}$, candy dispenser$^{(1)}$, water gun$^{(1)}$, duck$^{(1)}$

Figure S142: Participants's free-form generation of pretend objects for the item soap dispenser. Frequency count of the term for a pretend option is annotated as superscript.

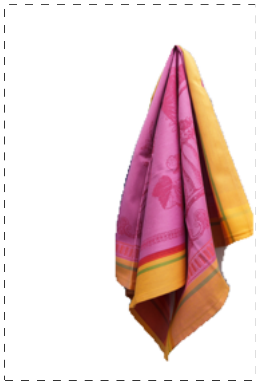

cape$^{(5)}$, blanket$^{(5)}$, dress$^{(3)}$, umbrella$^{(3)}$, curtain$^{(2)}$, scarf$^{(2)}$, invisibility cloak$^{(1)}$, washcloth$^{(1)}$, diamond$^{(1)}$, sheet$^{(1)}$, skirt$^{(1)}$, veil$^{(1)}$, paper air plane$^{(1)}$, tent$^{(1)}$, handkerchief$^{(1)}$, magic carpet$^{(1)}$, folded umbrella$^{(1)}$, robe$^{(1)}$, parachute$^{(1)}$, hair$^{(1)}$, flag$^{(1)}$, headscarf$^{(1)}$, gravel$^{(1)}$, superhero cape$^{(1)}$, hat$^{(1)}$, turkish turban$^{(1)}$

Figure S143: Participants's free-form generation of pretend objects for the item towel. Frequency count of the term for a pretend option is annotated as superscript.

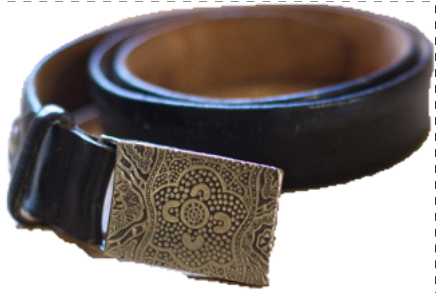

snake$^{(12)}$, whip$^{(6)}$, rope$^{(5)}$, lasso$^{(2)}$, leash$^{(2)}$, whip for a cowboy$^{(1)}$, wallet$^{(1)}$, suspenders$^{(1)}$, dog collar$^{(1)}$, watch$^{(1)}$, road$^{(1)}$, bracelet$^{(1)}$, indiana jones's whip$^{(1)}$, parachute strap$^{(1)}$, extension cord$^{(1)}$, headband$^{(1)}$, scarf$^{(1)}$, fire hose reel$^{(1)}$

Figure S144: Participants's free-form generation of pretend objects for the item belt. Frequency count of the term for a pretend option is annotated as superscript.

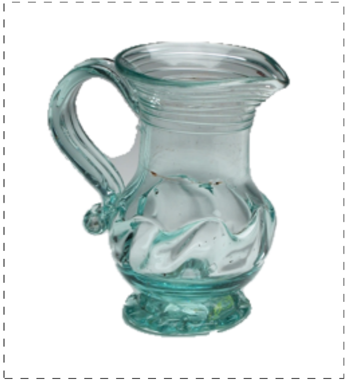

vase$^{(6)}$, pitcher$^{(2)}$, fountain$^{(2)}$, creature with its hand on its hip$^{(1)}$, lantern$^{(1)}$, cup$^{(1)}$, handbag$^{(1)}$, owl$^{(1)}$, kitten$^{(1)}$, hose$^{(1)}$, swimming pool$^{(1)}$, fish tank$^{(1)}$, nothing other than jug$^{(1)}$, treasure$^{(1)}$, trophy$^{(1)}$, table decor$^{(1)}$, tower$^{(1)}$, drinking glass$^{(1)}$, weird swimming pool$^{(1)}$, turbulent lake or wave$^{(1)}$, sculpture$^{(1)}$, fancy dress$^{(1)}$, watering can$^{(1)}$, valuable trading commodity$^{(1)}$, cave$^{(1)}$, planter$^{(1)}$, door knob$^{(1)}$, bird$^{(1)}$, device that captures evil spirits.$^{(1)}$, fishbowl$^{(1)}$, cat statue$^{(1)}$, fat lady$^{(1)}$, tree trunk$^{(1)}$

Figure S145: Participants's free-form generation of pretend objects for the item jug. Frequency count of the term for a pretend option is annotated as superscript.

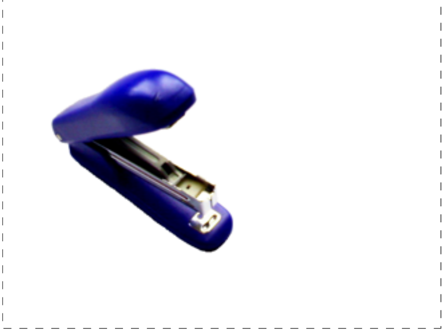

alligator$^{(10)}$, purple letter "v" block for making words$^{(1)}$, paperclip$^{(1)}$, mobile phone$^{(1)}$, holepunch$^{(1)}$, bite$^{(1)}$, tape$^{(1)}$, vegetable chopper$^{(1)}$, torch light$^{(1)}$, scissor$^{(1)}$, trash compactor$^{(1)}$, pacman$^{(1)}$, jaws$^{(1)}$, monster$^{(1)}$, crocodile$^{(1)}$, exerciser$^{(1)}$, monster mouth$^{(1)}$, alien from the movie alien$^{(1)}$, animal$^{(1)}$, pac man$^{(1)}$, weapon$^{(1)}$, pistol$^{(1)}$, shark$^{(1)}$, butterfly knife$^{(1)}$, mouth$^{(1)}$, switchblade$^{(1)}$, sheet paper$^{(1)}$, gun$^{(1)}$, crab$^{(1)}$, crocodile jaw$^{(1)}$, jaws of an alligator$^{(1)}$

Figure S146: Participants's free-form generation of pretend objects for the item stapler. Frequency count of the term for a pretend option is annotated as superscript.

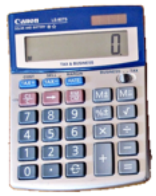

computer$^{(8)}$, phone$^{(5)}$, part of the command center on the starship enterprise$^{(1)}$, robotic assistant$^{(1)}$, mattress$^{(1)}$, robot$^{(1)}$, watch$^{(1)}$, book$^{(1)}$, television$^{(1)}$, controller$^{(1)}$, paper$^{(1)}$, bed$^{(1)}$, door lock keypad$^{(1)}$, spy device$^{(1)}$, pinpad on an atm$^{(1)}$, old phone$^{(1)}$, supercomputer$^{(1)}$, telephone$^{(1)}$, smart phone$^{(1)}$, keyboard$^{(1)}$, spaceship control panel$^{(1)}$, spaceship dashboard$^{(1)}$, stapler$^{(1)}$, typewriter$^{(1)}$, secret vault key-lock$^{(1)}$, cellphone$^{(1)}$, futuristic phone$^{(1)}$, cash register$^{(1)}$, control panel$^{(1)}$

Figure S147: Participants's free-form generation of pretend objects for the item calculator. Frequency count of the term for a pretend option is annotated as superscript.

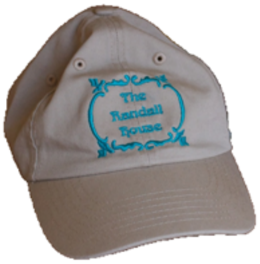

basket$^{(3)}$, bowl$^{(2)}$, bag$^{(2)}$, donald duck$^{(2)}$, cave$^{(2)}$, tent$^{(2)}$, part of a two factor authentication token for entering a house$^{(1)}$, scarf$^{(1)}$, halo$^{(1)}$, duck$^{(1)}$, beanie$^{(1)}$, cleaning cloth$^{(1)}$, umbrella$^{(1)}$, sidewalk$^{(1)}$, disc$^{(1)}$, washcloth$^{(1)}$, mountain$^{(1)}$, mountain in the distance$^{(1)}$, toy$^{(1)}$, kid$^{(1)}$, mask$^{(1)}$, boat$^{(1)}$, duck's face$^{(1)}$, crown$^{(1)}$, speed bumb$^{(1)}$, purse$^{(1)}$, bird's nest$^{(1)}$, frisbee$^{(1)}$, sombrero$^{(1)}$, grocery container$^{(1)}$, soldiers helmet$^{(1)}$, cup$^{(1)}$, hat$^{(1)}$

Figure S148: Participants's free-form generation of pretend objects for the item hat. Frequency count of the term for a pretend option is annotated as superscript.

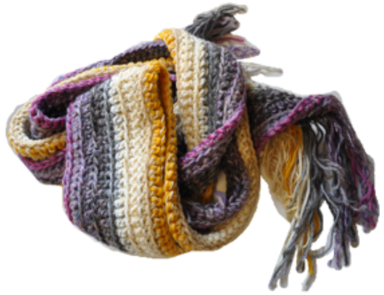

blanket$^{(4)}$, snake$^{(3)}$, hammock$^{(2)}$, coiled snake$^{(2)}$, snake that is in disguise$^{(1)}$, sweater$^{(1)}$, mummy wrappings$^{(1)}$, spagetti$^{(1)}$, ninja mask$^{(1)}$, throw up$^{(1)}$, hoodie$^{(1)}$, robe$^{(1)}$, pile of leaves$^{(1)}$, rag$^{(1)}$, tremor movie worm$^{(1)}$, spaghetti$^{(1)}$, rug$^{(1)}$, python$^{(1)}$, valley$^{(1)}$, long road$^{(1)}$, shaggy snake coiled in on itself$^{(1)}$, animal$^{(1)}$, african hat$^{(1)}$, trap$^{(1)}$, hair$^{(1)}$, cocoon$^{(1)}$, feather boa$^{(1)}$, sash$^{(1)}$, duvet$^{(1)}$, tube top$^{(1)}$, eel$^{(1)}$, belt$^{(1)}$, crumpled newspaper$^{(1)}$

Figure S149: Participants's free-form generation of pretend objects for the item scarf. Frequency count of the term for a pretend option is annotated as superscript.

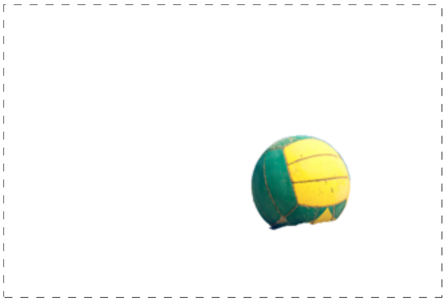

basketball$^{(3)}$, helmet$^{(3)}$, wilson$^{(2)}$, soccer ball$^{(2)}$, planet$^{(2)}$, missile$^{(1)}$, wilson from cast away$^{(1)}$, snorlax$^{(1)}$, softball$^{(1)}$, globe$^{(1)}$, lemon$^{(1)}$, face$^{(1)}$, head$^{(1)}$, rock$^{(1)}$, seat$^{(1)}$, paper weight$^{(1)}$, turtle$^{(1)}$, mountain$^{(1)}$, friend you know like that movie i forget what he called him though$^{(1)}$, wilson, tom hanks friend$^{(1)}$, belly$^{(1)}$, giant boulder$^{(1)}$, boulder$^{(1)}$, distant planet$^{(1)}$, magic ball$^{(1)}$, sandbag$^{(1)}$, grenade$^{(1)}$, headrest$^{(1)}$, friend$^{(1)}$, soccerball$^{(1)}$, hat$^{(1)}$, earth$^{(1)}$, paw ranger face$^{(1)}$

Figure S150: Participants's free-form generation of pretend objects for the item volleyball. Frequency count of the term for a pretend option is annotated as superscript.

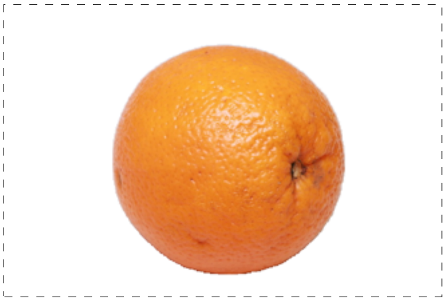

ball$^{(5)}$, basketball$^{(3)}$, sun$^{(3)}$, baseball$^{(3)}$, apple$^{(2)}$, lemon$^{(2)}$, tennis ball$^{(2)}$, fake orange that is actually a grenade$^{(1)}$, dragon egg$^{(1)}$, ping pong ball$^{(1)}$, lacrosse ball$^{(1)}$, mango$^{(1)}$, spaceship$^{(1)}$, porcupine$^{(1)}$, planet$^{(1)}$, persons naval$^{(1)}$, nose$^{(1)}$, diamond$^{(1)}$, magic 8 ball$^{(1)}$, bouncy ball$^{(1)}$, color$^{(1)}$, croquet ball$^{(1)}$, eyeball$^{(1)}$, tangerine$^{(1)}$, paper weight$^{(1)}$, the heart of your fallen enemies$^{(1)}$, planet mars$^{(1)}$

Figure S151: Participants's free-form generation of pretend objects for the item orange. Frequency count of the term for a pretend option is annotated as superscript.

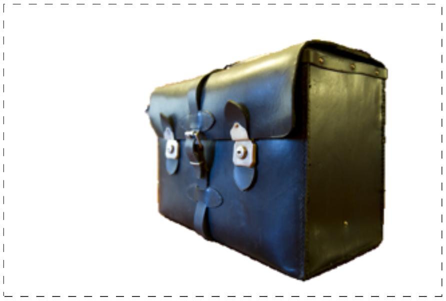

treasure chest$^{(5)}$, purse$^{(2)}$, suitcase$^{(2)}$, trunk$^{(2)}$, chest$^{(2)}$, safe$^{(2)}$, water dispenser$^{(1)}$, backpack$^{(1)}$, clutch$^{(1)}$, pet carrier$^{(1)}$, smiley face$^{(1)}$, battery$^{(1)}$, train$^{(1)}$, monster$^{(1)}$, stool$^{(1)}$, safe box$^{(1)}$, suitcase with money$^{(1)}$, doctor's bag$^{(1)}$, mailbox$^{(1)}$, square house$^{(1)}$, toolbox$^{(1)}$, computer$^{(1)}$, jail$^{(1)}$, bomb package$^{(1)}$, portal$^{(1)}$, treasure box$^{(1)}$, building block$^{(1)}$, end table$^{(1)}$, bomb$^{(1)}$, casket$^{(1)}$, pirate trunk$^{(1)}$

Figure S152: Participants's free-form generation of pretend objects for the item briefcase. Frequency count of the term for a pretend option is annotated as superscript.

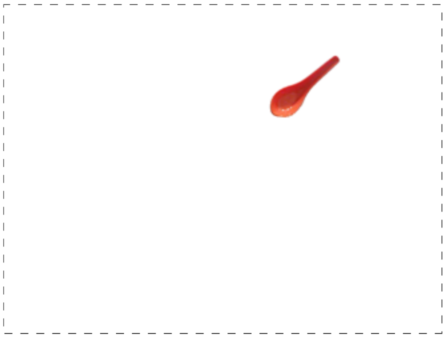

ladle$^{(3)}$, microphone$^{(3)}$, magic wand$^{(2)}$, knife$^{(2)}$, guitar$^{(2)}$, shovel$^{(2)}$, dowsing rod$^{(1)}$, eye cover$^{(1)}$, compass needle$^{(1)}$, dinosaur$^{(1)}$, vein$^{(1)}$, tablespoon$^{(1)}$, sperm$^{(1)}$, meteorite$^{(1)}$, elfs spoon$^{(1)}$, tear drop$^{(1)}$, thermometer$^{(1)}$, bath for insects$^{(1)}$, pool$^{(1)}$, drum stick$^{(1)}$, arrow$^{(1)}$, tool$^{(1)}$, wooden paddle$^{(1)}$, pipe$^{(1)}$, tiny pool$^{(1)}$, wand$^{(1)}$, paper airplane$^{(1)}$, boat$^{(1)}$, container for change$^{(1)}$, wizards wand$^{(1)}$, paddle$^{(1)}$, lizard tongue$^{(1)}$

Figure S153: Participants's free-form generation of pretend objects for the item soup spoon. Frequency count of the term for a pretend option is annotated as superscript.

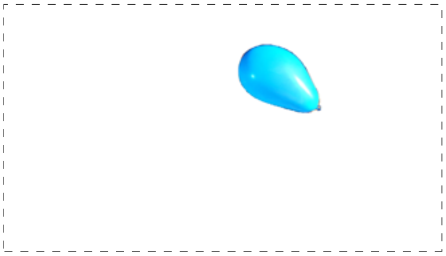

ball$^{(4)}$, pillow$^{(3)}$, cloud$^{(2)}$, pear$^{(2)}$, egg$^{(2)}$, machine that sucks all the oxygen out of the air, forever growing larger and devastating the earth$^{(1)}$, blimp$^{(1)}$, biohazard container$^{(1)}$, jellybean$^{(1)}$, tear$^{(1)}$, eggplant$^{(1)}$, sun$^{(1)}$, teardrop$^{(1)}$, pokemon$^{(1)}$, meteor$^{(1)}$, teddy bear$^{(1)}$, bubbles$^{(1)}$, low gravity ball$^{(1)}$, slug$^{(1)}$, prostate$^{(1)}$, fruit$^{(1)}$, grenade$^{(1)}$, rain cloud$^{(1)}$, ostrich egg$^{(1)}$, stress ball$^{(1)}$, plastic bag$^{(1)}$, soap bubble$^{(1)}$, bubble$^{(1)}$, helium$^{(1)}$, the kamehameha wave$^{(1)}$, phone$^{(1)}$, alien face$^{(1)}$

Figure S154: Participants's free-form generation of pretend objects for the item balloon. Frequency count of the term for a pretend option is annotated as superscript.

E Supplemental Information for Study 4

Figure S155-S178 visualize people's responses in the PRETENSE and PRIOR conditions, as well as the coding of the location of subparts in model-inpainted images. Inpainting images were sampled from state-of-art language-vision models, including DALLE-2 (Ramesh et al., 2022) and Stable Diffusion (Rombach et al., 2022). Table 1 list the item-specific information for the subpart mapping task. For each real object, there are three images taken of different views of the object, as shown across Figure S155-S178.

Real object	Pretend object	Subpart 1	Subpart 2
mug	elephant	trunk	left ear
broccoli	tree	trunk	crown
stapler	crocodile	mouth	tail
teapot	duck	beak	tail
wineglass	tulip	petal	stem
pear	snowman	head	body
car	frog	mouth	right hind leg
bottle	rocket	nosecone	fin

Table 1: Overview of the pretend object and its subparts to be located for each of the 8 categories of real objects in the stimuli set for Study 4.

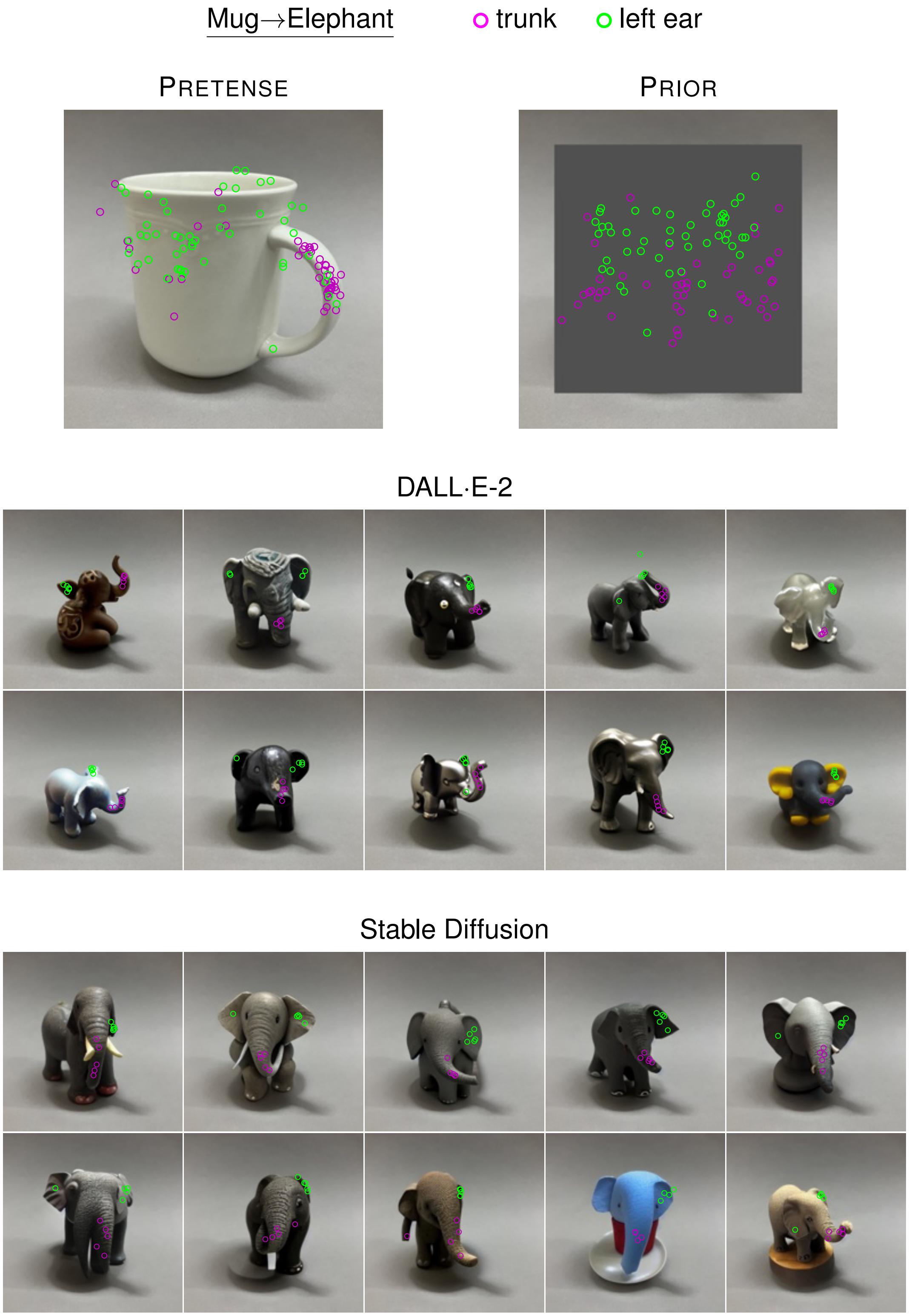

Figure S155: People's responses in the Pretense and Prior conditions, as well as coding of model-inpainted images. Participants were prompted to pretend the mug is an elephant. Models inpainted the masked area of the mug given the prompt "An elephant".

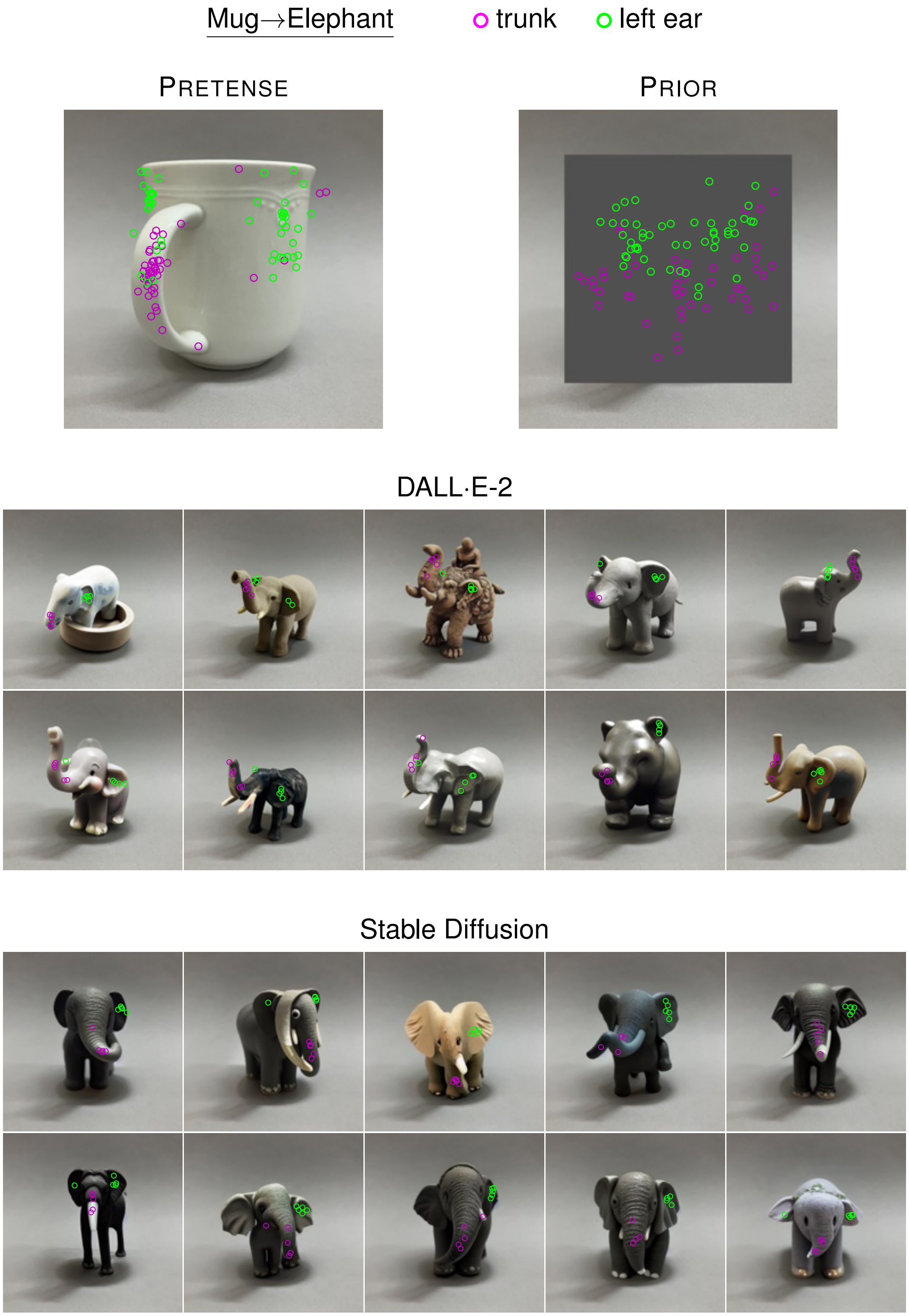

Figure S156: People's responses in the PRETENSE and PRIOR conditions, as well as coding of model-inpainted images. Participants were prompted to pretend the mug is an elephant. Models inpainted the masked area of the mug given the prompt "An elephant".

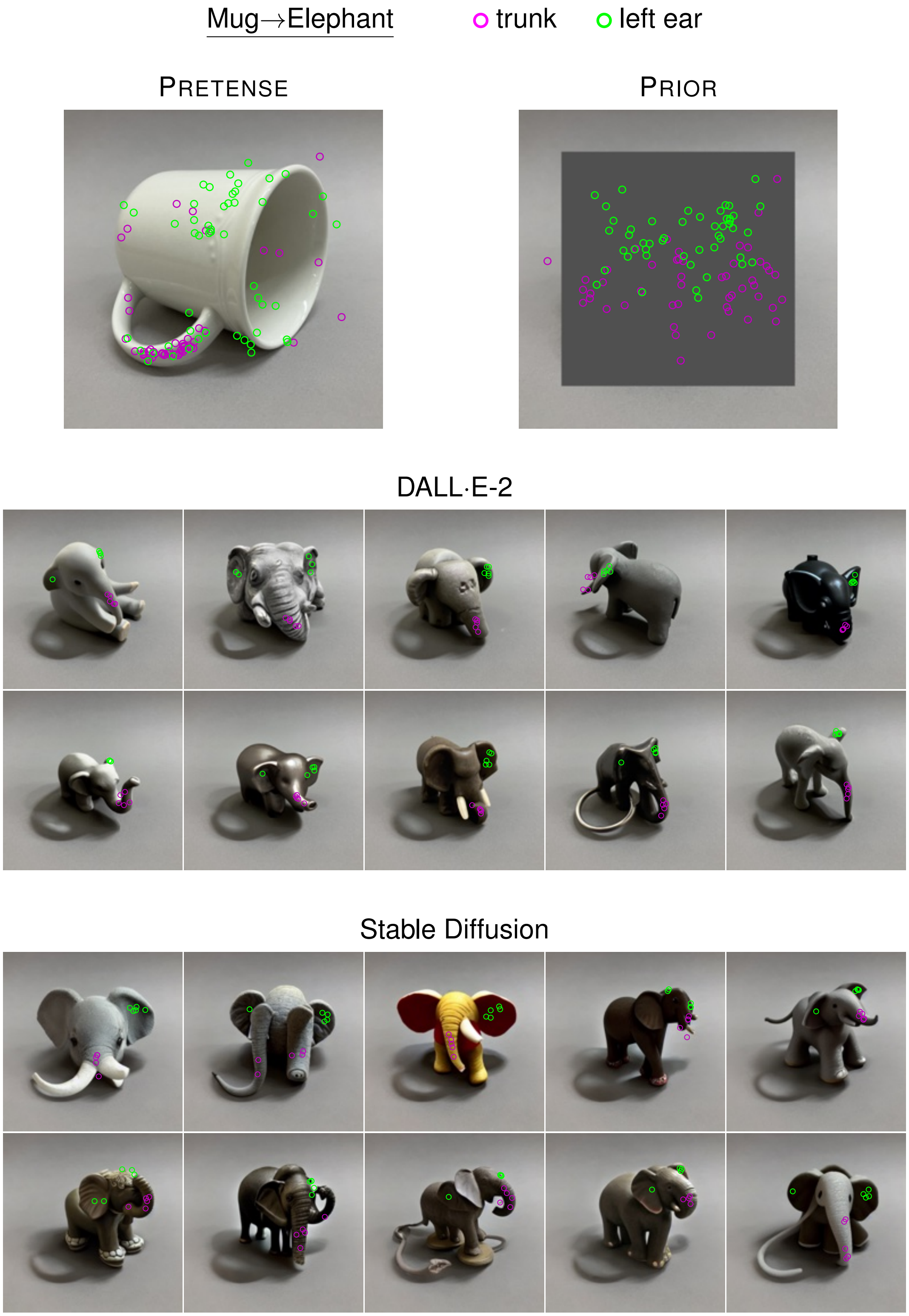

Figure S157: People's responses in the PRETENSE and PRIOR conditions, as well as coding of model-inpainted images. Participants were prompted to pretend the mug is an elephant. Models inpainted the masked area of the mug given the prompt "An elephant".

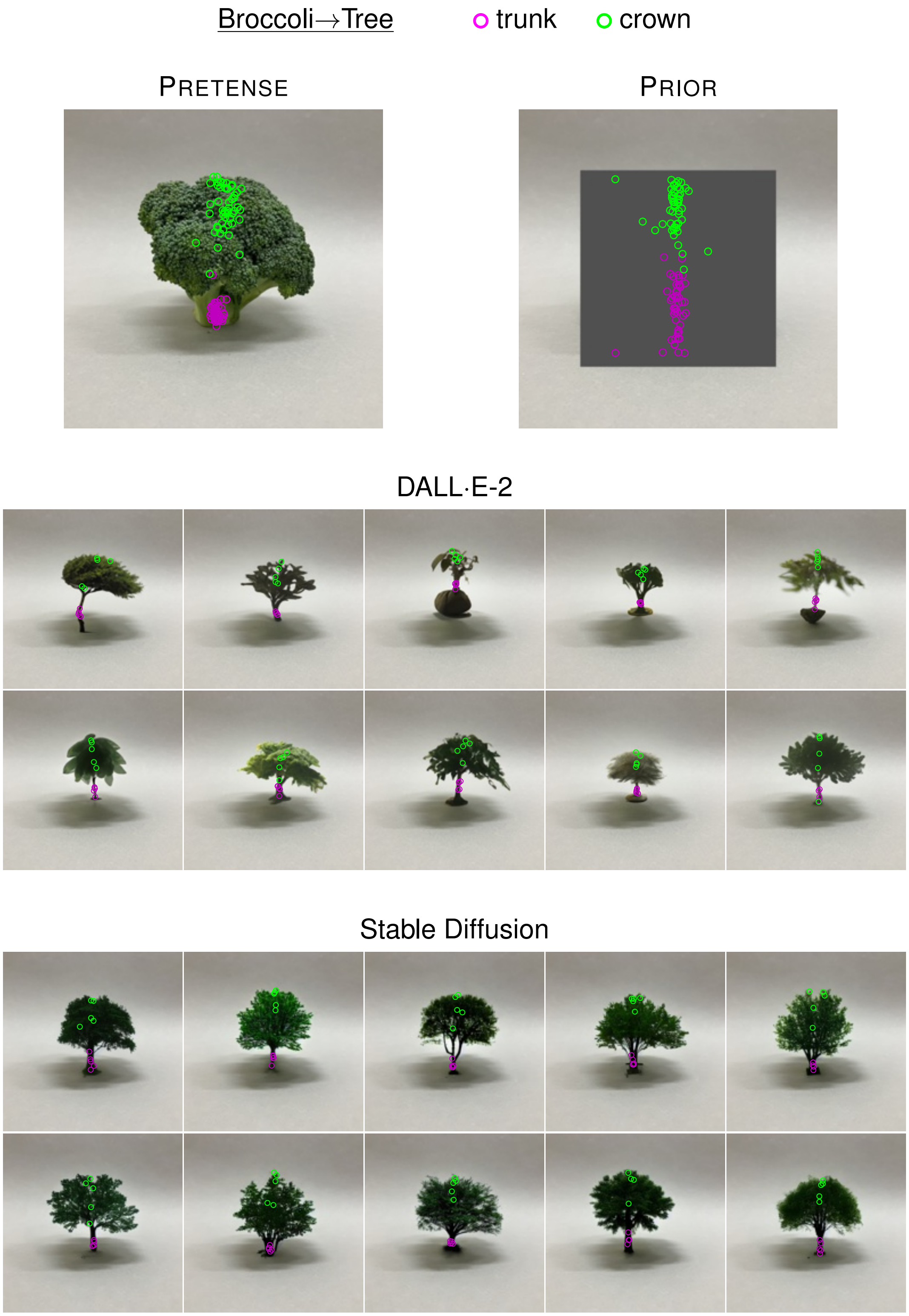

Figure S158: People's responses in the PRETENSE and PRIOR conditions, as well as coding of model-inpainted images. Participants were prompted to pretend the broccoli is a tree. Models inpainted the masked area of the broccoli given the prompt "A tree".

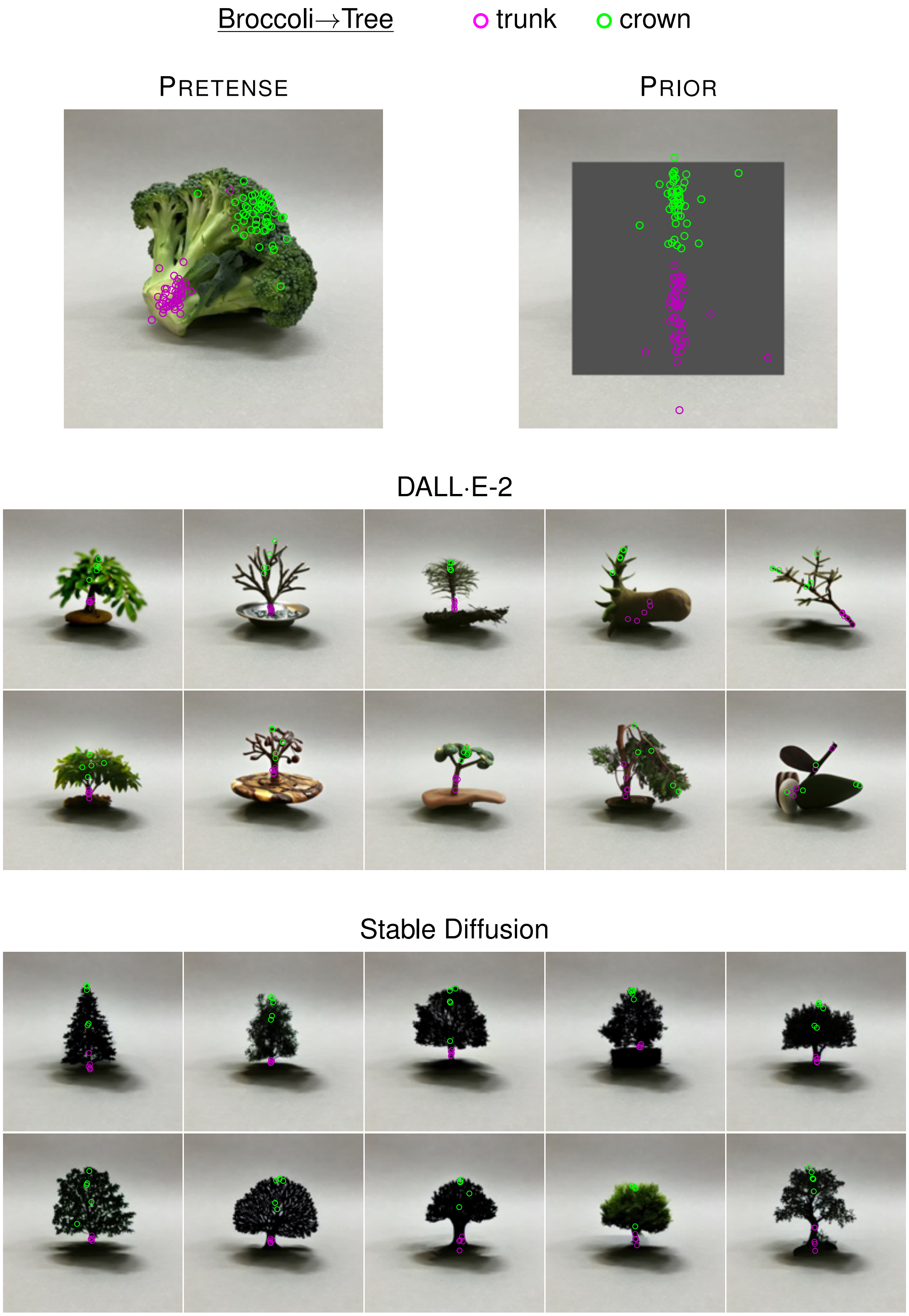

Figure S159: People's responses in the PRETENSE and PRIOR conditions, as well as coding of model-inpainted images. Participants were prompted to pretend the broccoli is a tree. Models inpainted the masked area of the broccoli given the prompt "A tree".

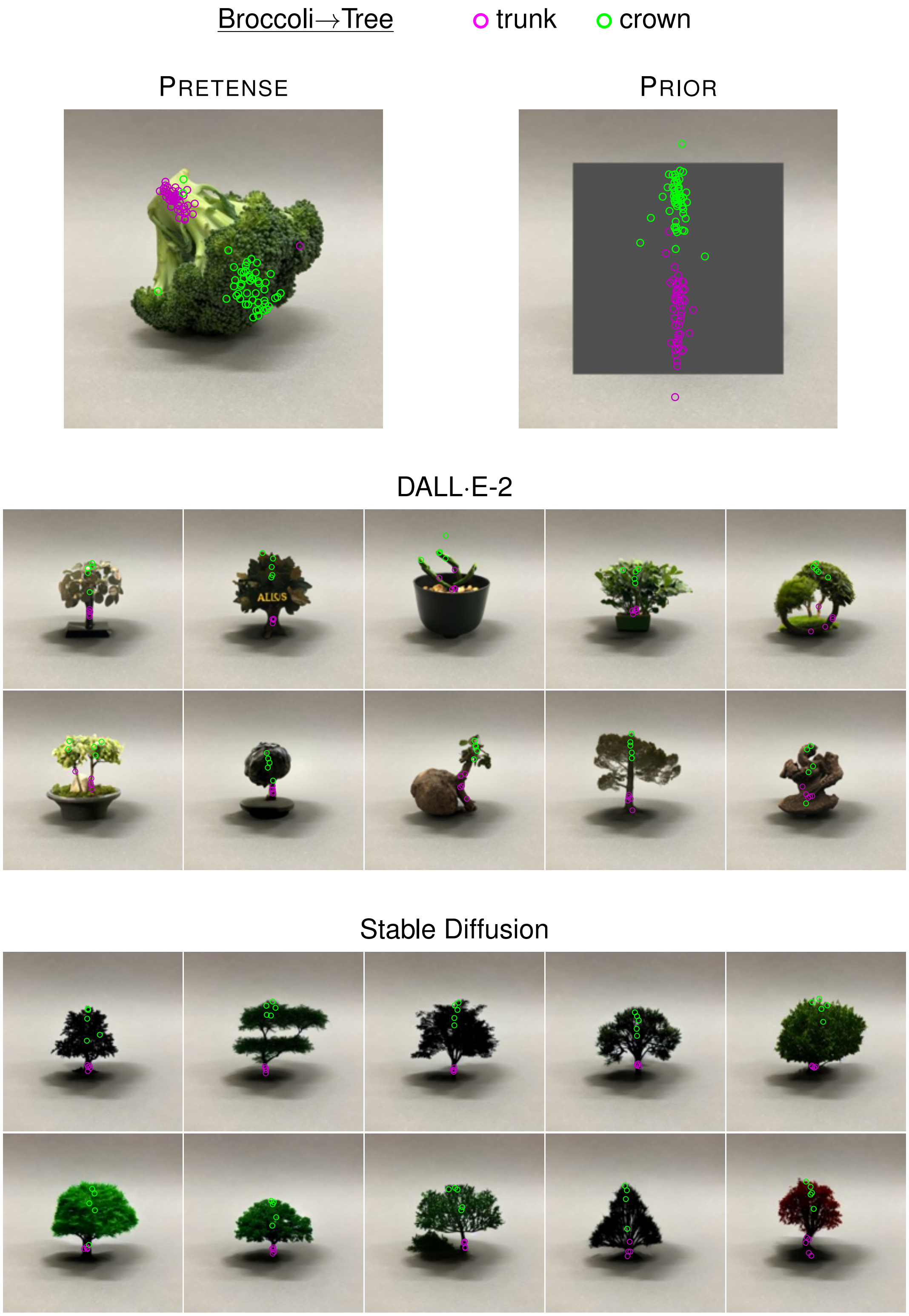

Figure S160: People's responses in the PRETENSE and PRIOR conditions, as well as coding of model-inpainted images. Participants were prompted to pretend the broccoli is a tree. Models inpainted the masked area of the broccoli given the prompt "A tree".

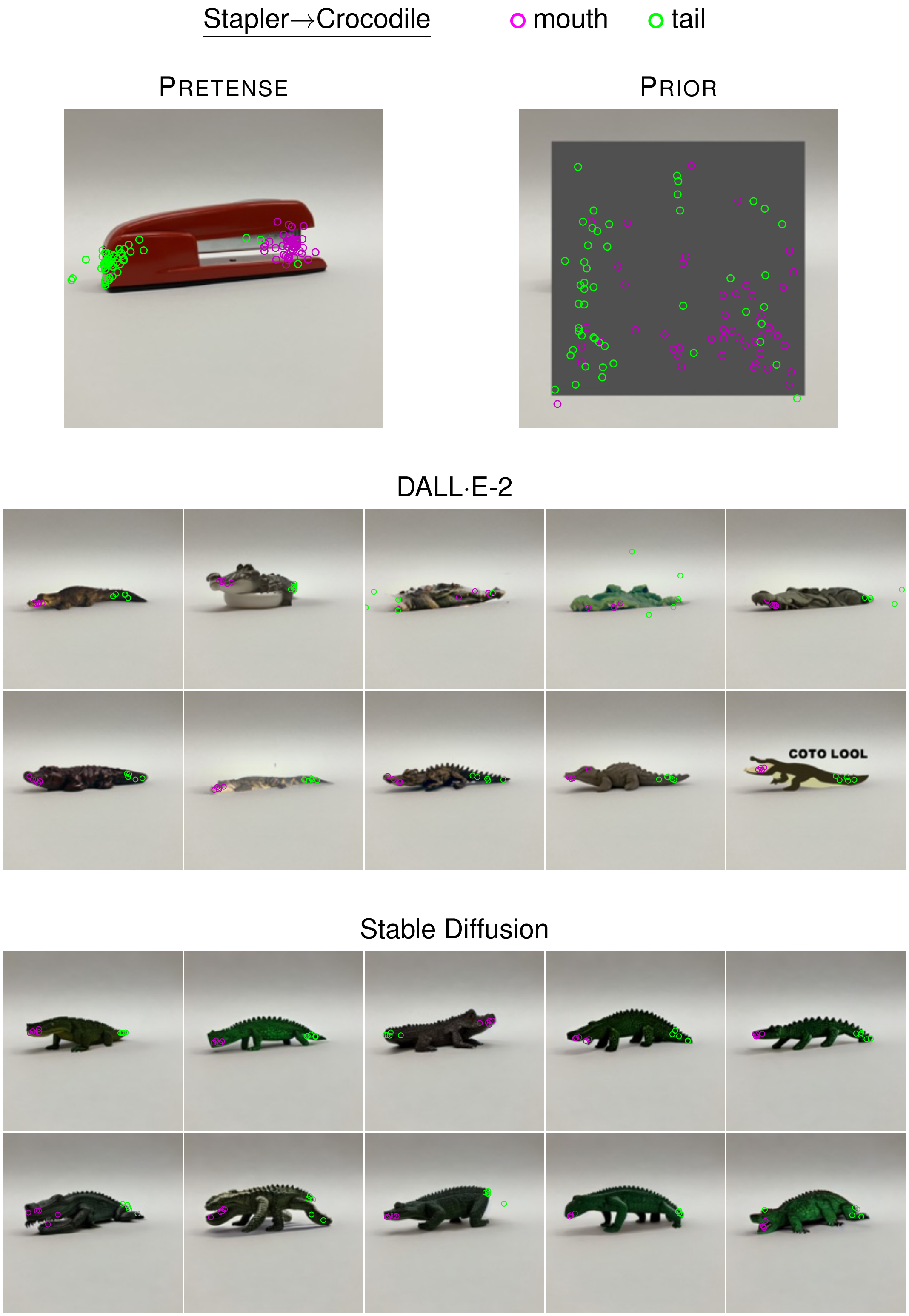

Figure S161: People's responses in the PRETENSE and PRIOR conditions, as well as coding of model-inpainted images. Participants were prompted to pretend the stapler is a crocodile. Models inpainted the masked area of the stapler given the prompt "A crocodile".

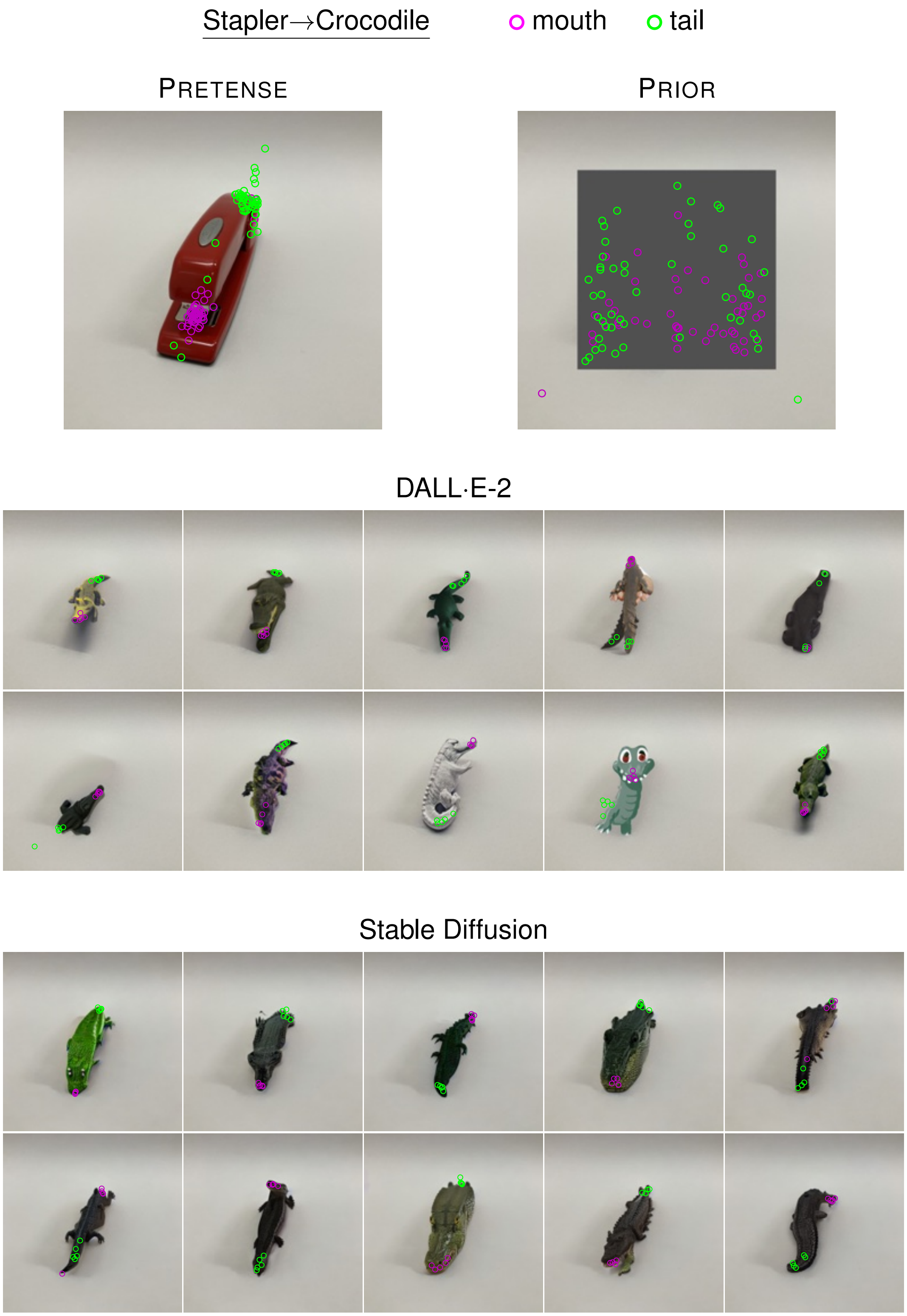

Figure S162: People's responses in the PRETENSE and PRIOR conditions, as well as coding of model-inpainted images. Participants were prompted to pretend the stapler is a crocodile. Models inpainted the masked area of the stapler given the prompt "A crocodile".

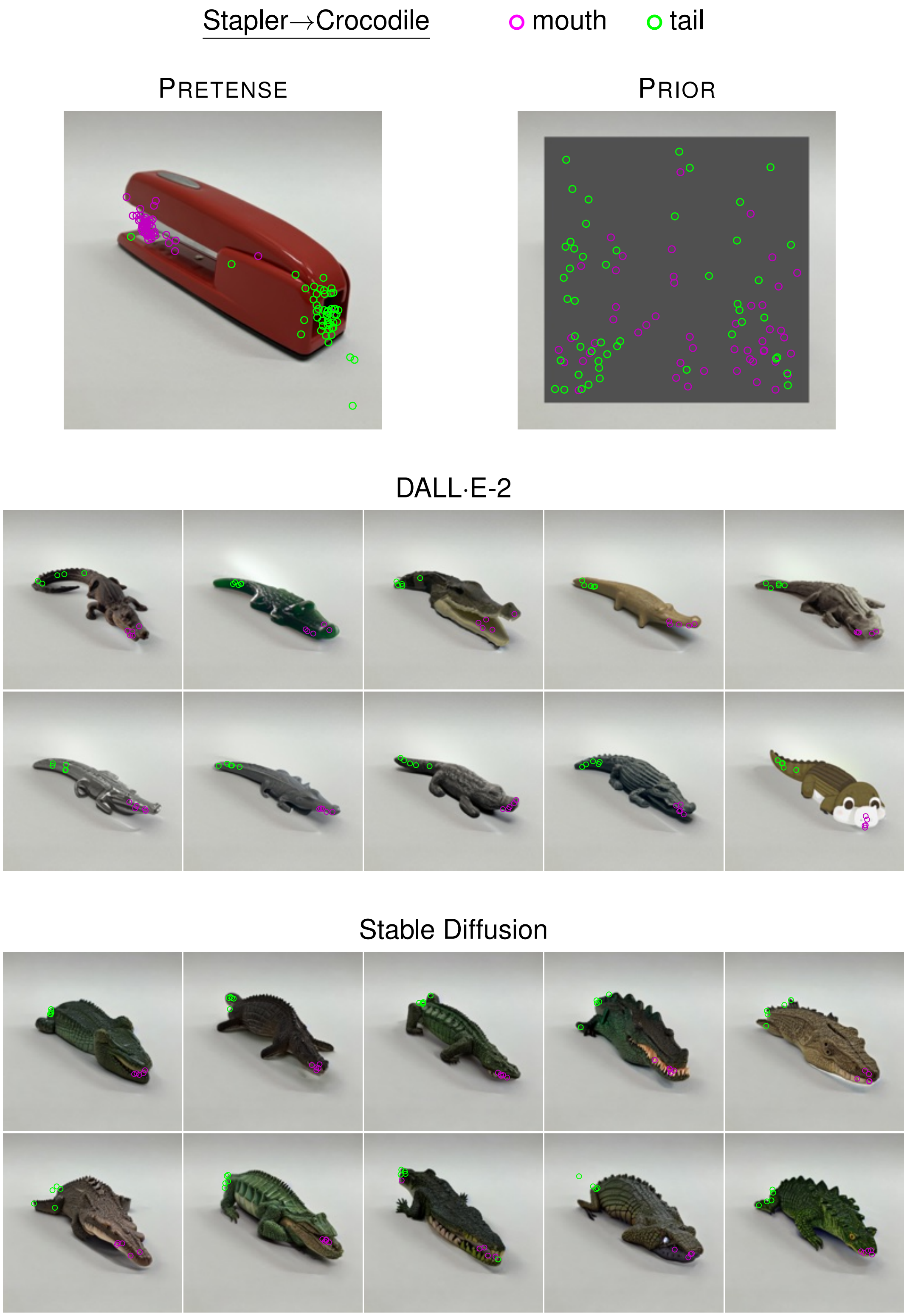

Figure S163: People's responses in the PRETENSE and PRIOR conditions, as well as coding of model-inpainted images. Participants were prompted to pretend the stapler is a crocodile. Models inpainted the masked area of the stapler given the prompt "A crocodile".

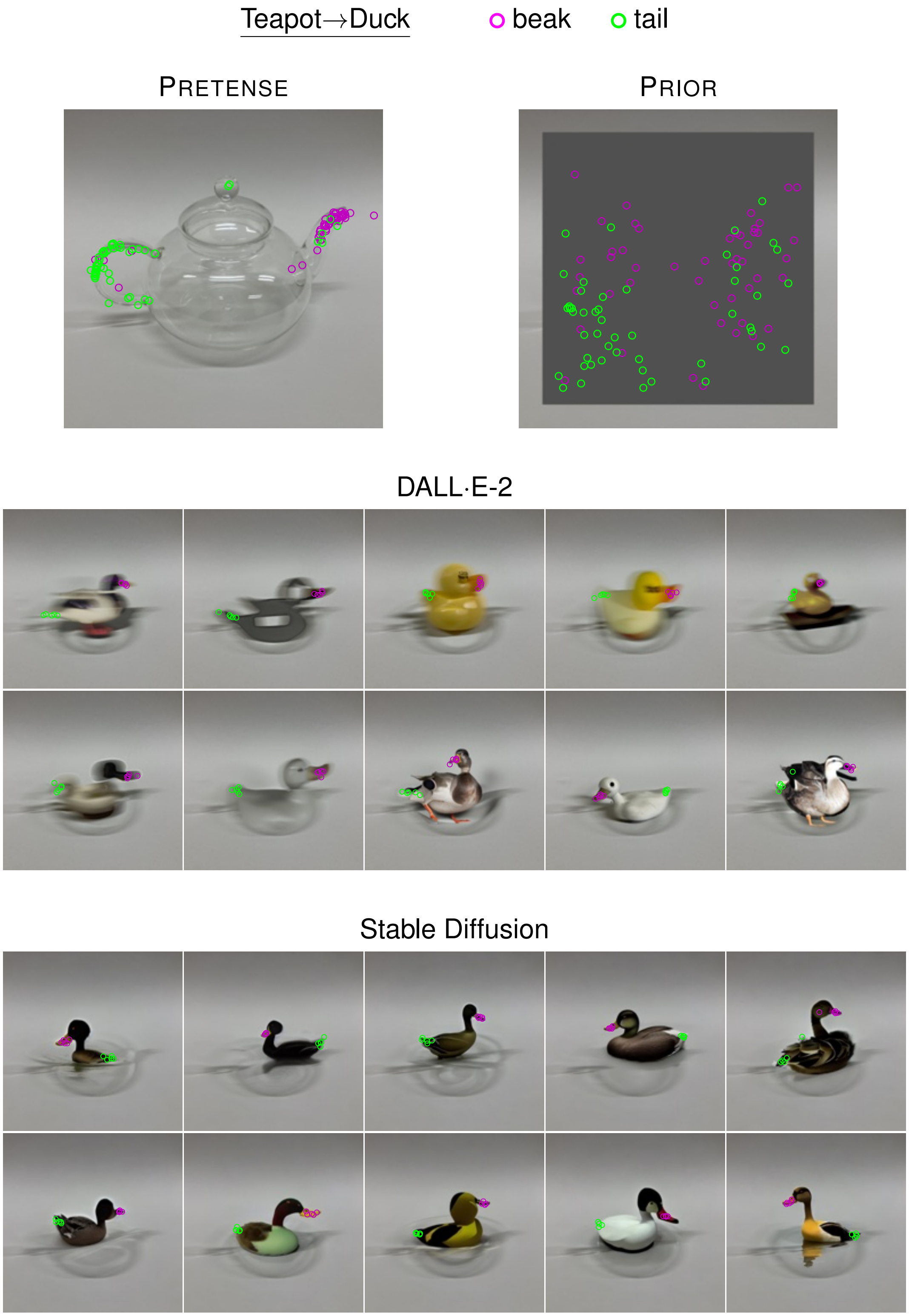

Figure S164: People's responses in the PRETENSE and PRIOR conditions, as well as coding of model-inpainted images. Participants were prompted to pretend the teapot is a duck. Models inpainted the masked area of the teapot given the prompt "A duck".

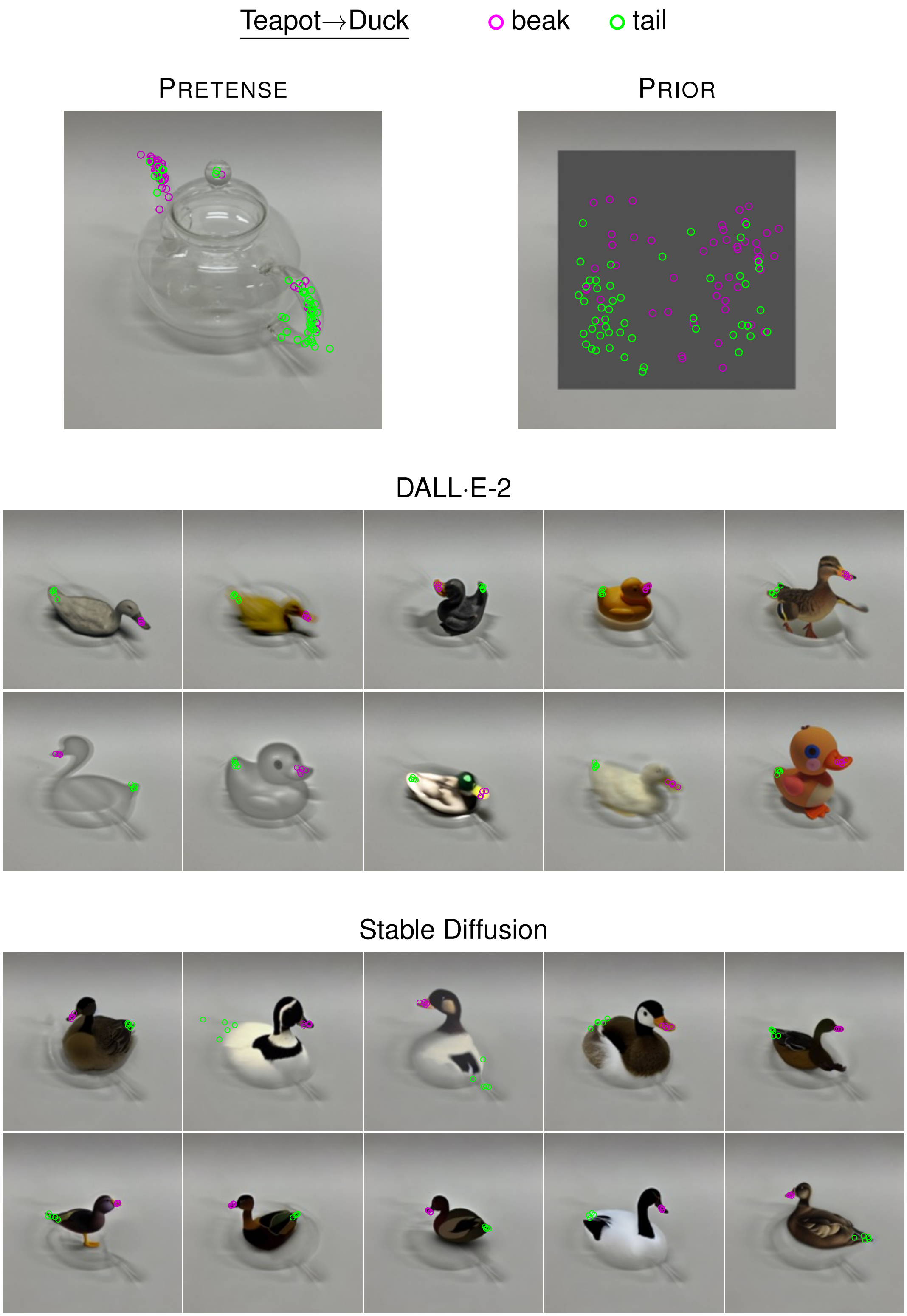

Figure S165: People's responses in the PRETENSE and PRIOR conditions, as well as coding of model-inpainted images. Participants were prompted to pretend the teapot is a duck. Models inpainted the masked area of the teapot given the prompt "A duck".

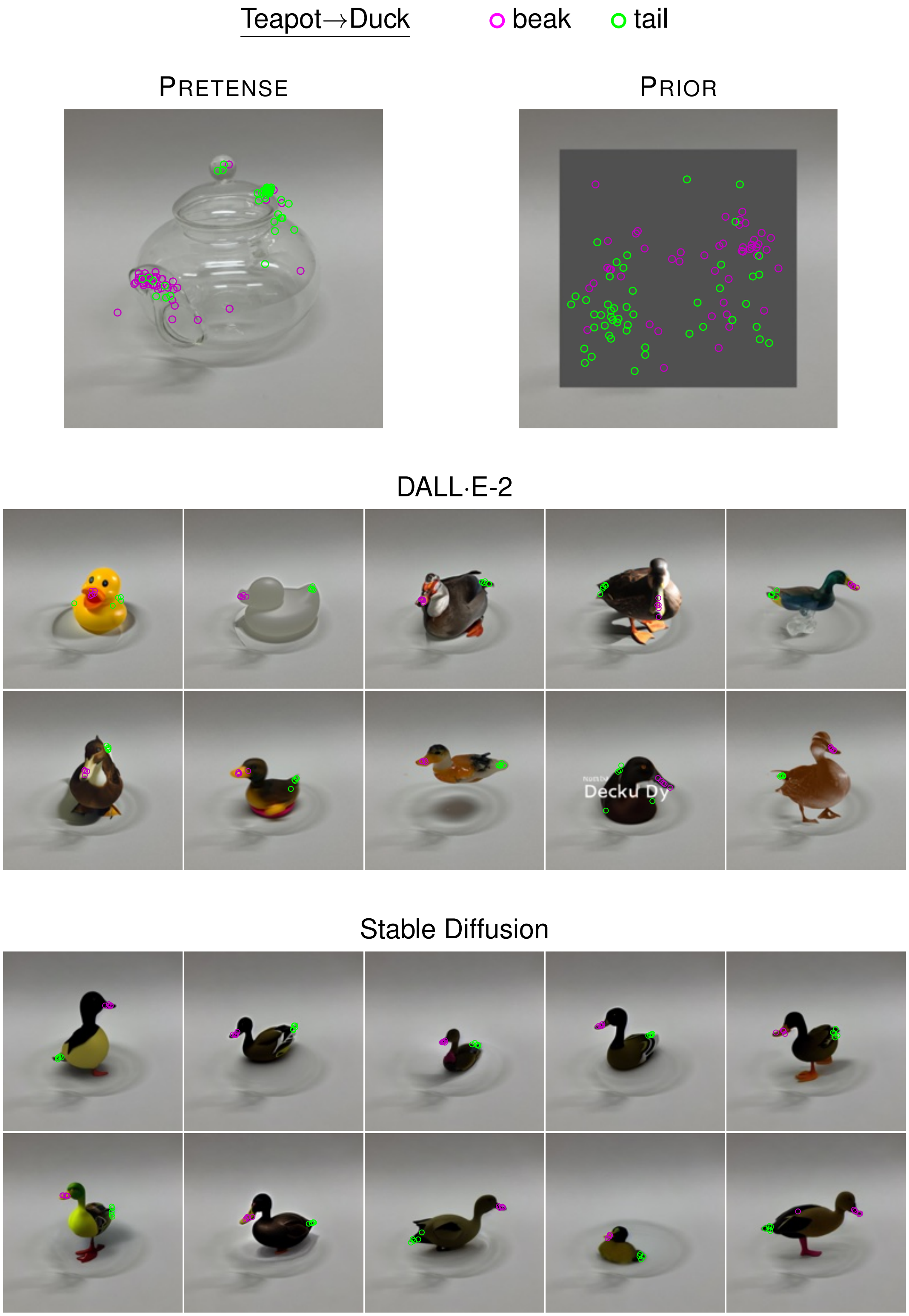

Figure S166: People's responses in the PRETENSE and PRIOR conditions, as well as coding of model-inpainted images. Participants were prompted to pretend the teapot is a duck. Models inpainted the masked area of the teapot given the prompt "A duck".

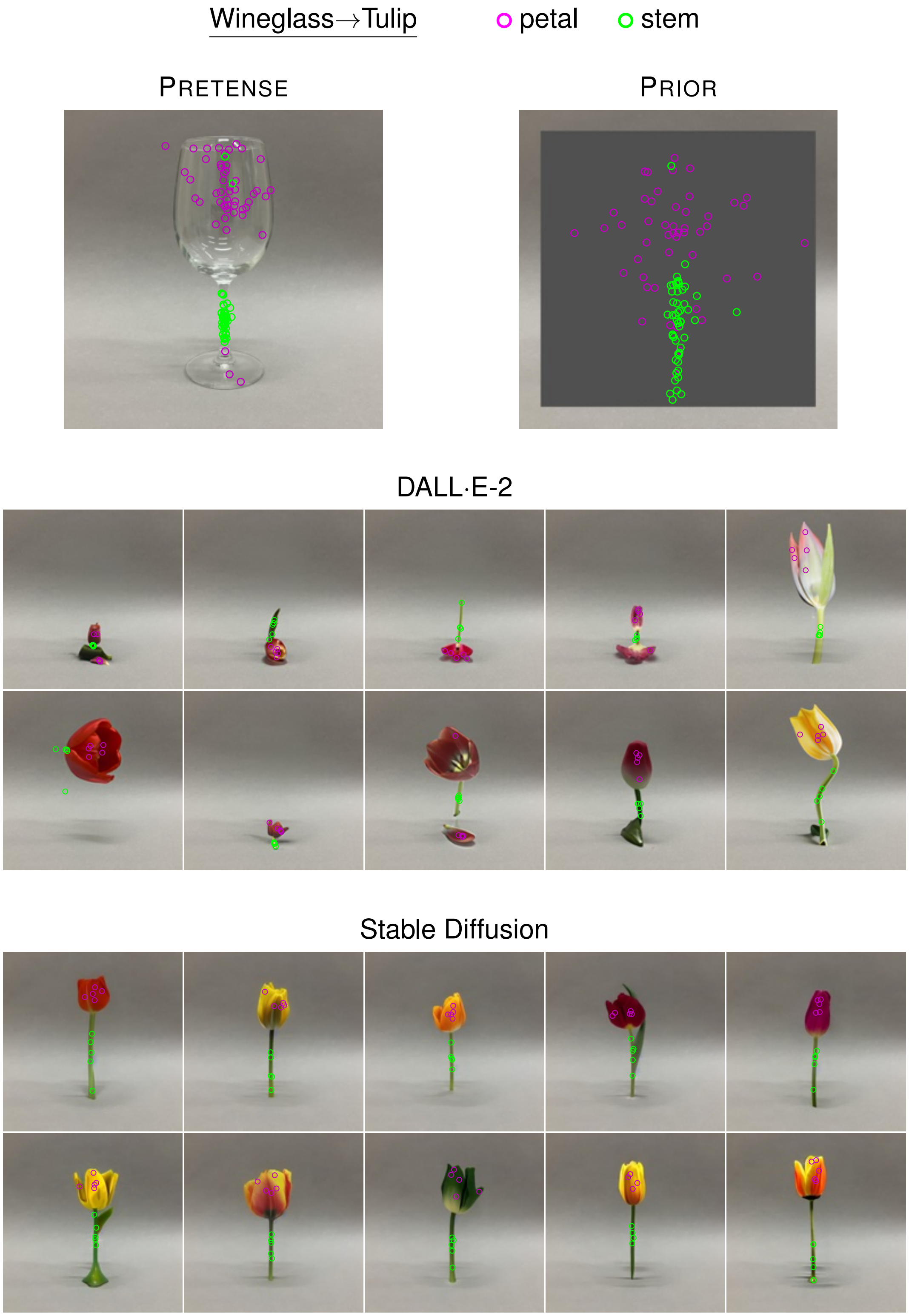

Figure S167: People's responses in the PRETENSE and PRIOR conditions, as well as coding of model-inpainted images. Participants were prompted to pretend the wineglass is a tulip. Models inpainted the masked area of the wineglass given the prompt "A tulip".

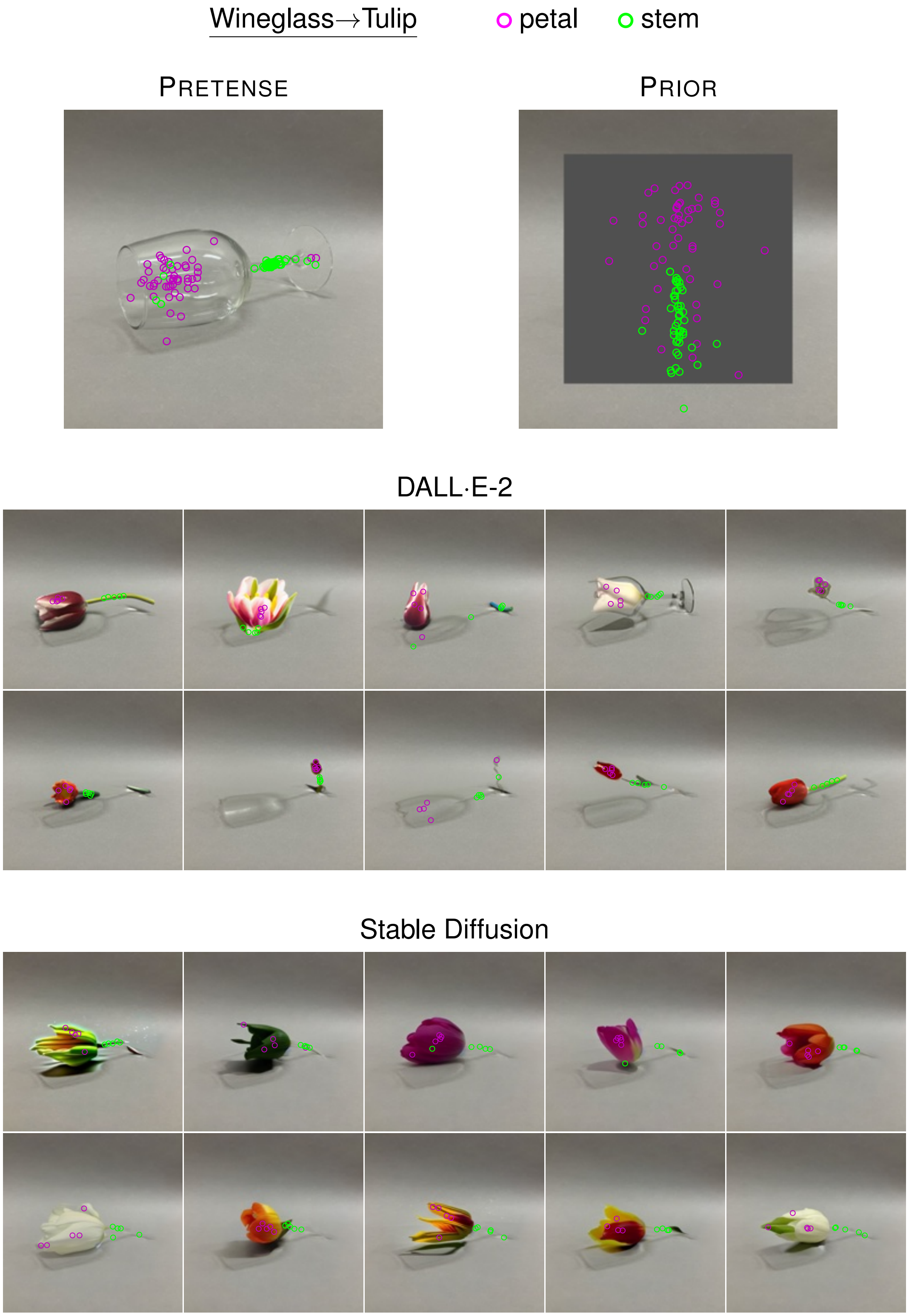

Figure S168: People's responses in the PRETENSE and PRIOR conditions, as well as coding of model-inpainted images. Participants were prompted to pretend the wineglass is a tulip. Models inpainted the masked area of the wineglass given the prompt "A tulip".

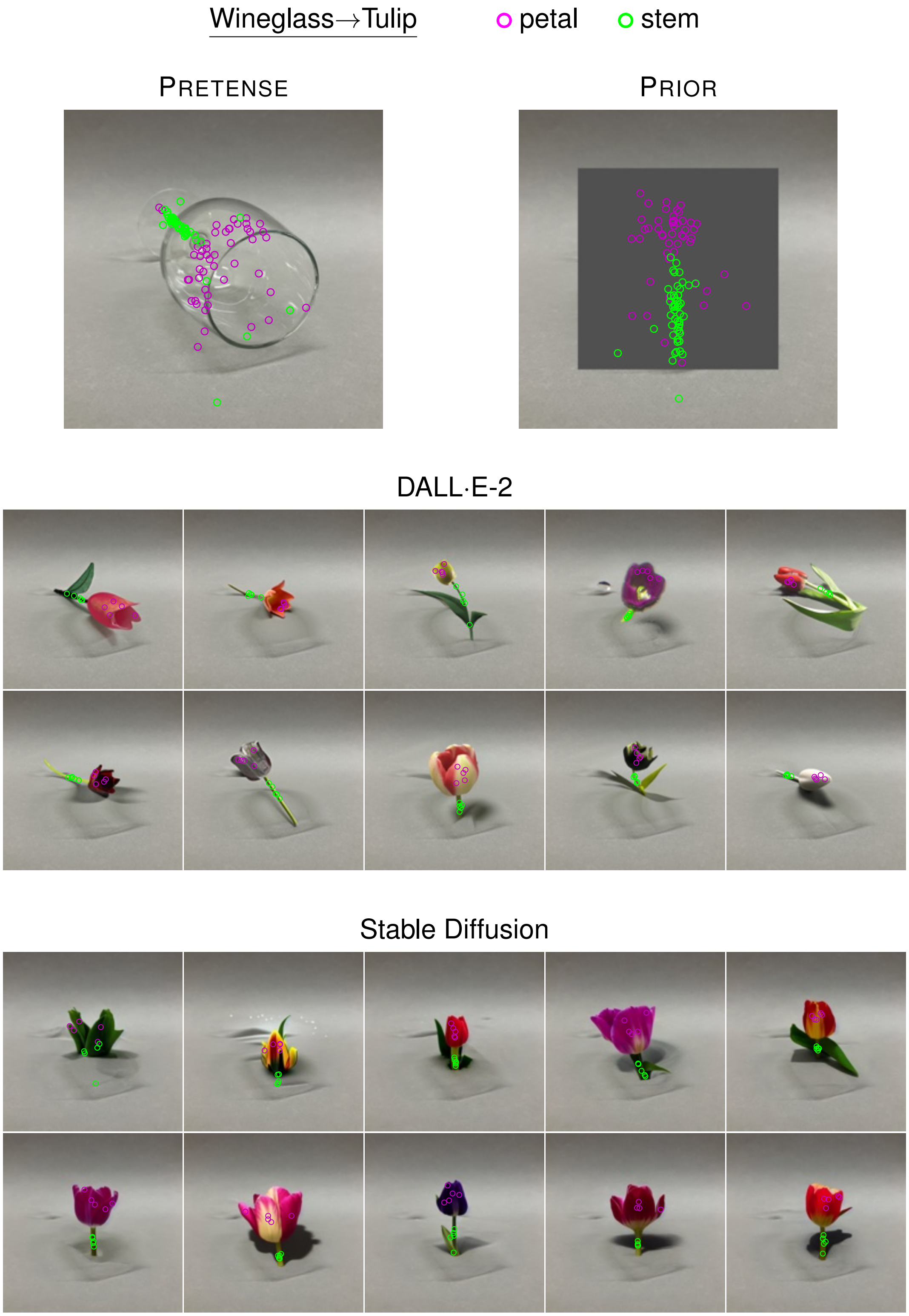

Figure S169: People's responses in the PRETENSE and PRIOR conditions, as well as coding of model-inpainted images. Participants were prompted to pretend the wineglass is a tulip. Models inpainted the masked area of the wineglass given the prompt "A tulip".

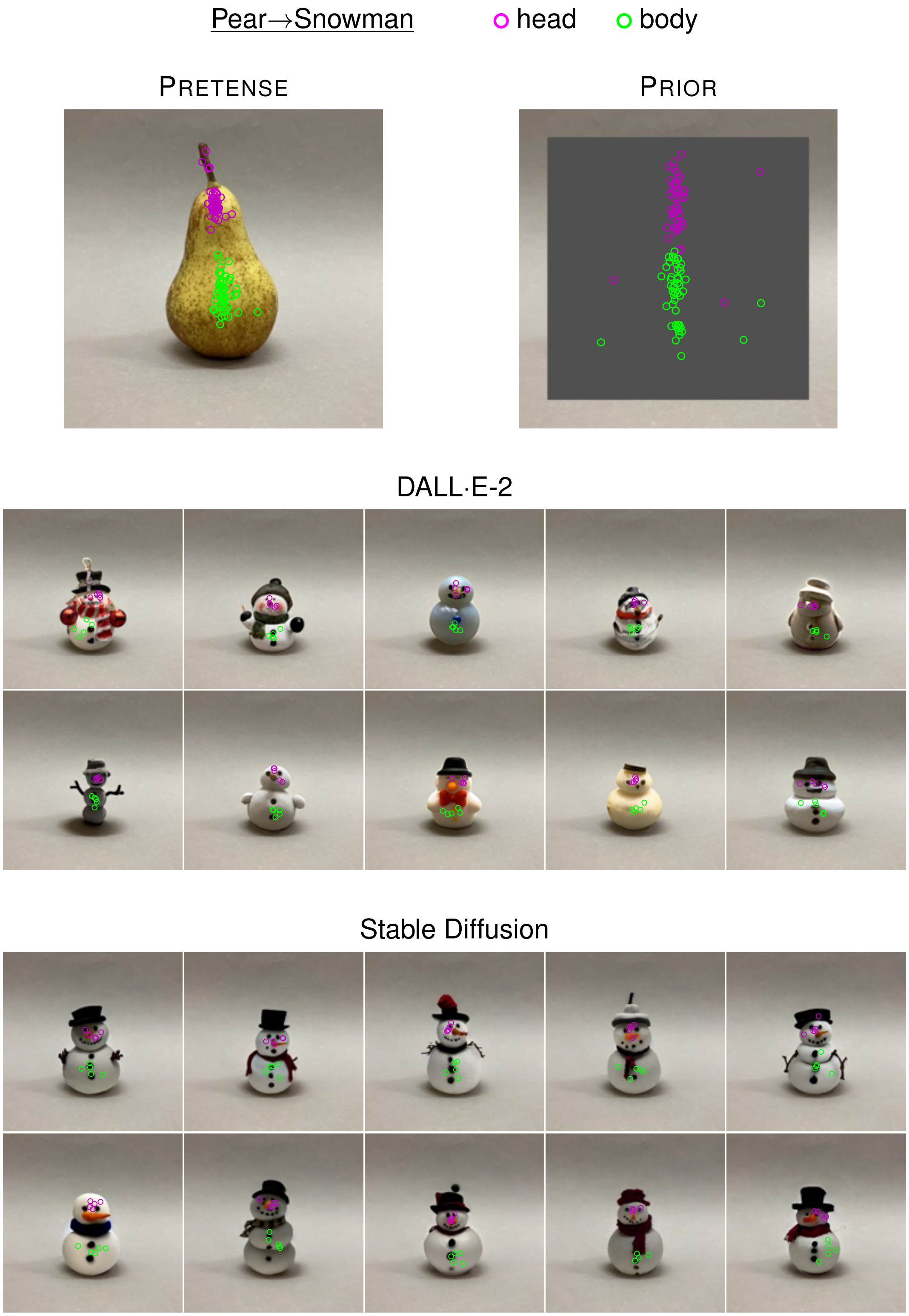

Figure S170: People's responses in the PRETENSE and PRIOR conditions, as well as coding of model-inpainted images. Participants were prompted to pretend the pear is a snowman. Models inpainted the masked area of the pear given the prompt "A snowman".

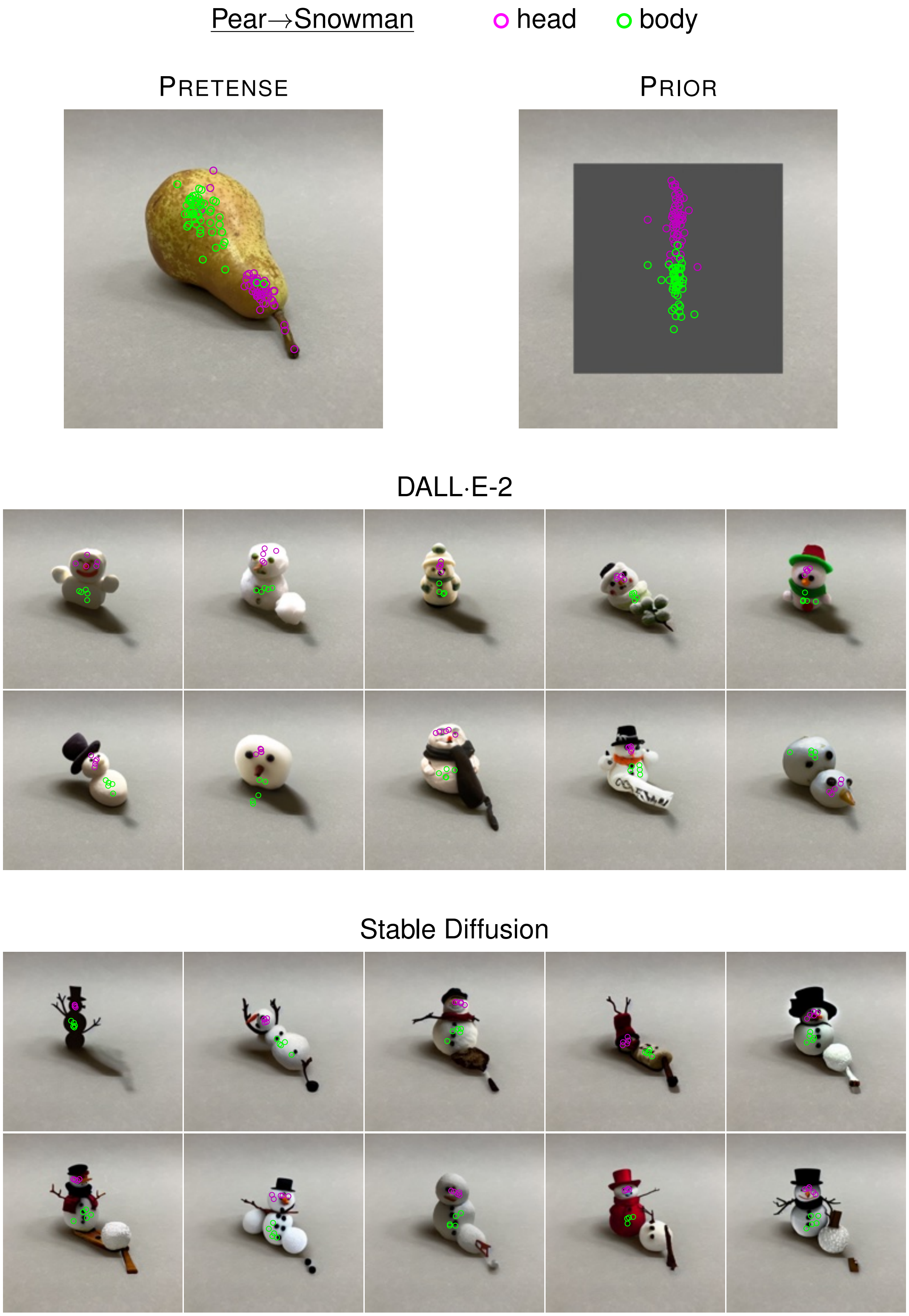

Figure S171: People's responses in the PRETENSE and PRIOR conditions, as well as coding of model-inpainted images. Participants were prompted to pretend the pear is a snowman. Models inpainted the masked area of the pear given the prompt "A snowman".

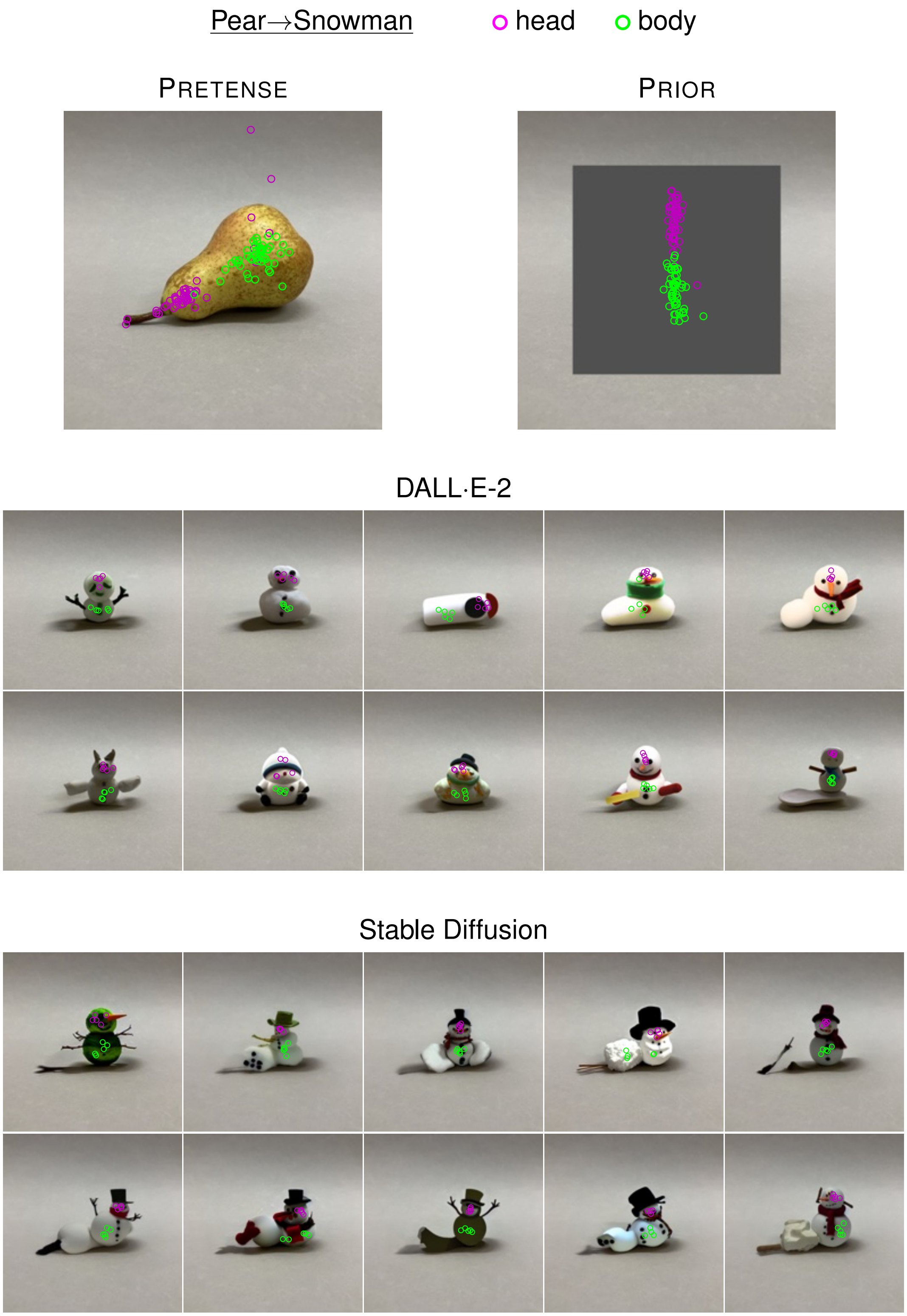

Figure S172: People's responses in the PRETENSE and PRIOR conditions, as well as coding of model-inpainted images. Participants were prompted to pretend the pear is a snowman. Models inpainted the masked area of the pear given the prompt "A snowman".

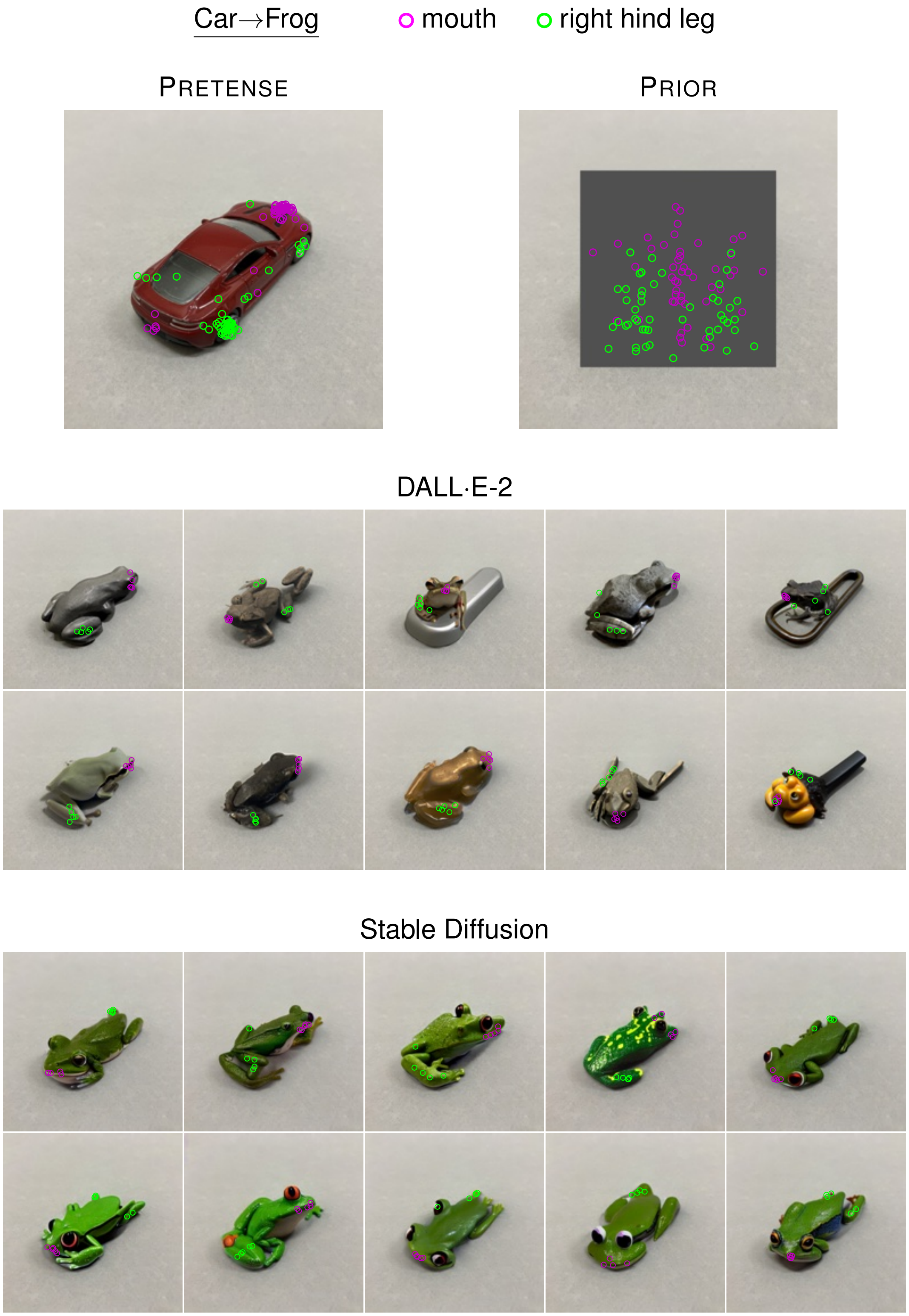

Figure S173: People's responses in the PRETENSE and PRIOR conditions, as well as coding of model-inpainted images. Participants were prompted to pretend the car is a frog. Models inpainted the masked area of the car given the prompt "A frog".

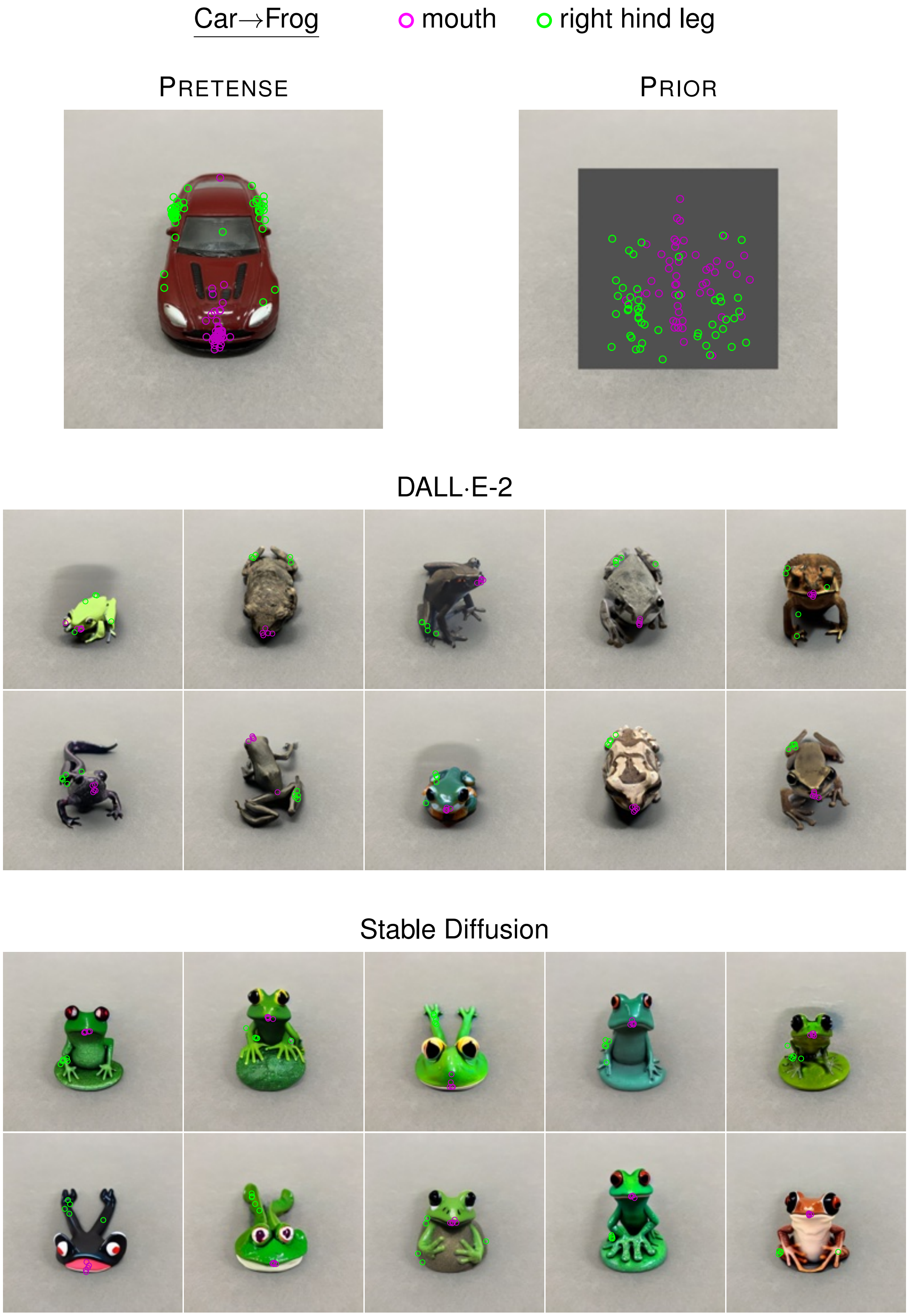

Figure S174: People's responses in the PRETENSE and PRIOR conditions, as well as coding of model-inpainted images. Participants were prompted to pretend the car is a frog. Models inpainted the masked area of the car given the prompt "A frog".

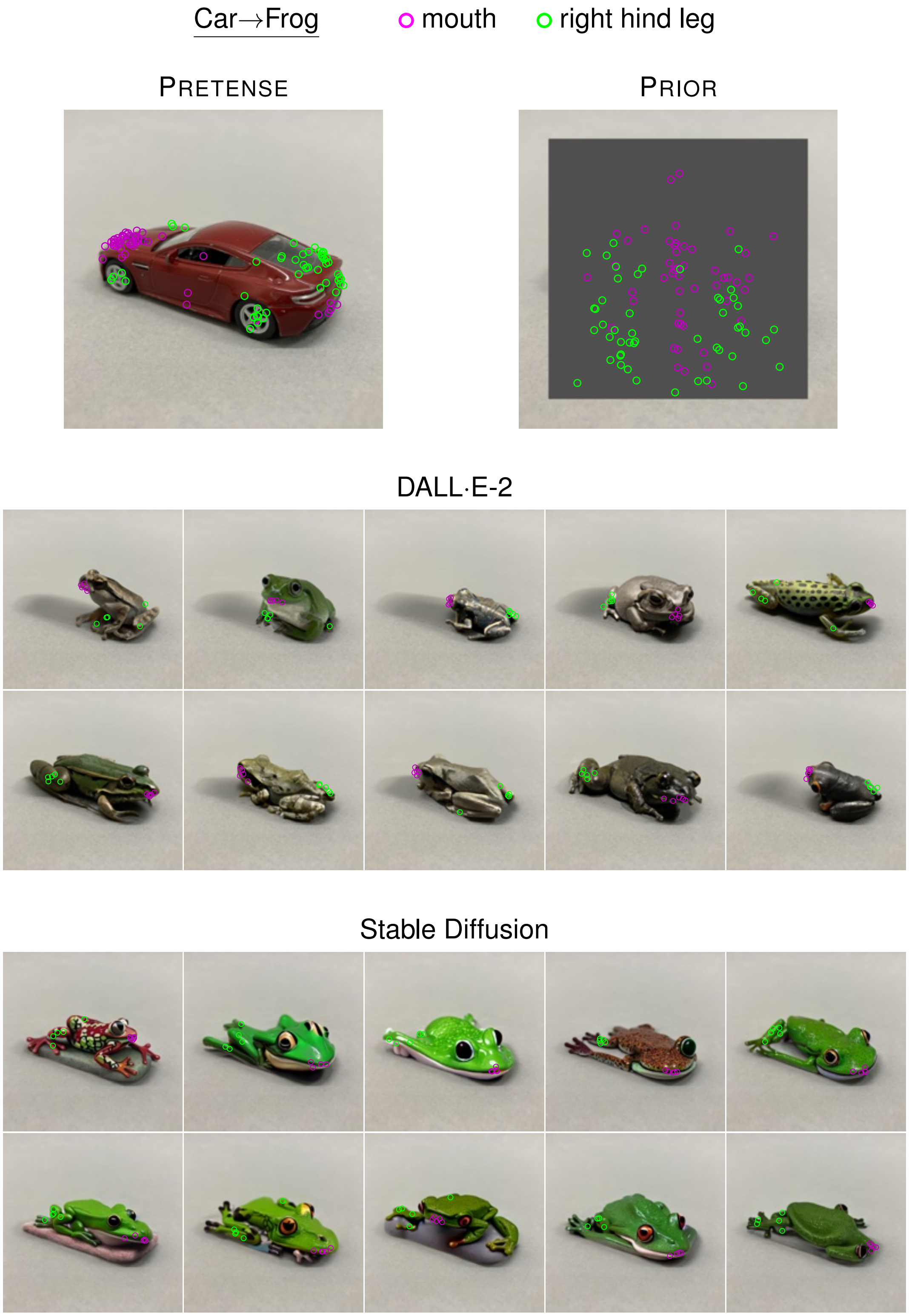

Figure S175: People's responses in the PRETENSE and PRIOR conditions, as well as coding of model-inpainted images. Participants were prompted to pretend the car is a frog. Models inpainted the masked area of the car given the prompt "A frog".

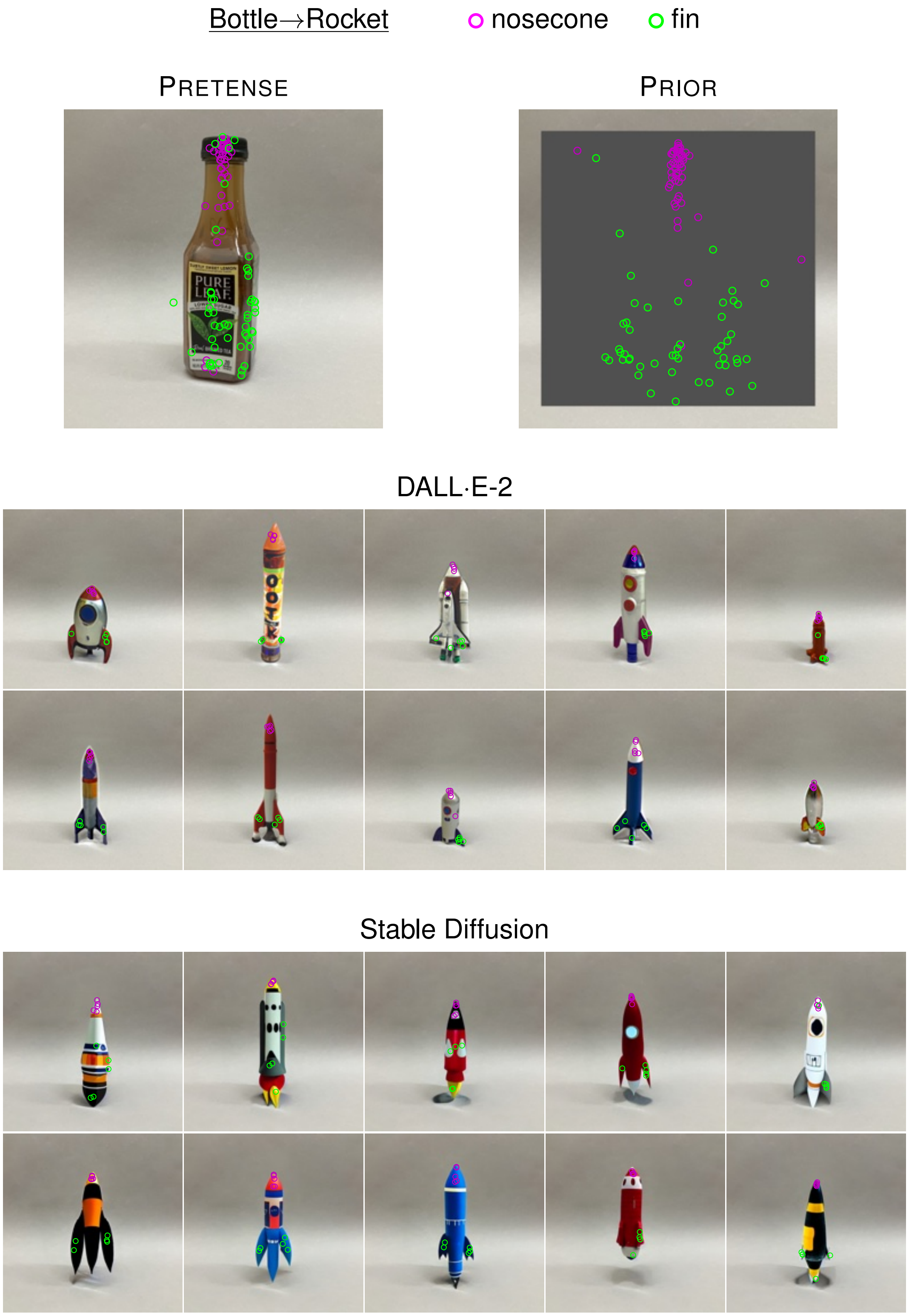

Figure S176: People's responses in the PRETENSE and PRIOR conditions, as well as coding of model-inpainted images. Participants were prompted to pretend the bottle is a rocket. Models inpainted the masked area of the bottle given the prompt "A rocket".

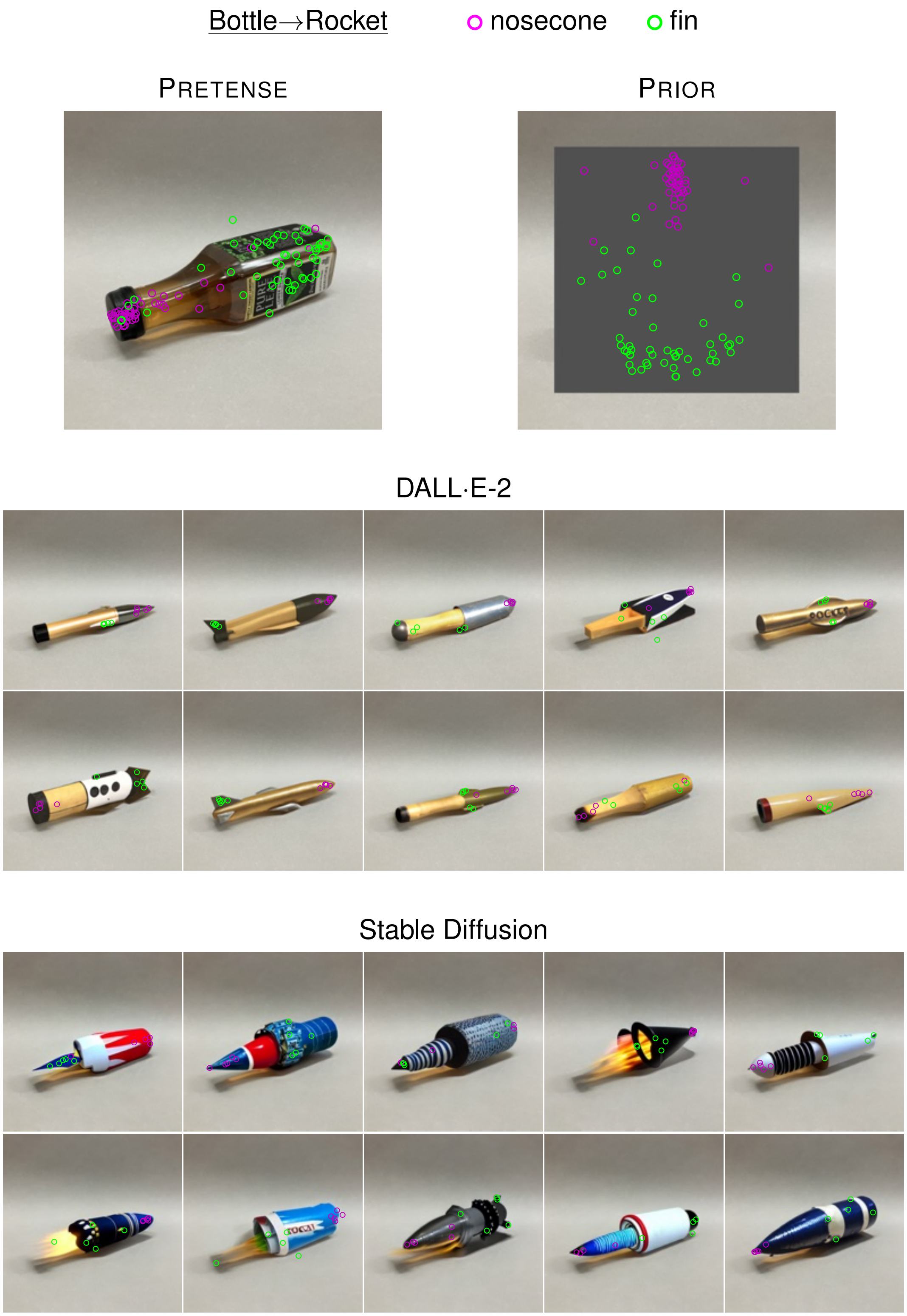

Figure S177: People's responses in the PRETENSE and PRIOR conditions, as well as coding of model-inpainted images. Participants were prompted to pretend the bottle is a rocket. Models inpainted the masked area of the bottle given the prompt "A rocket".

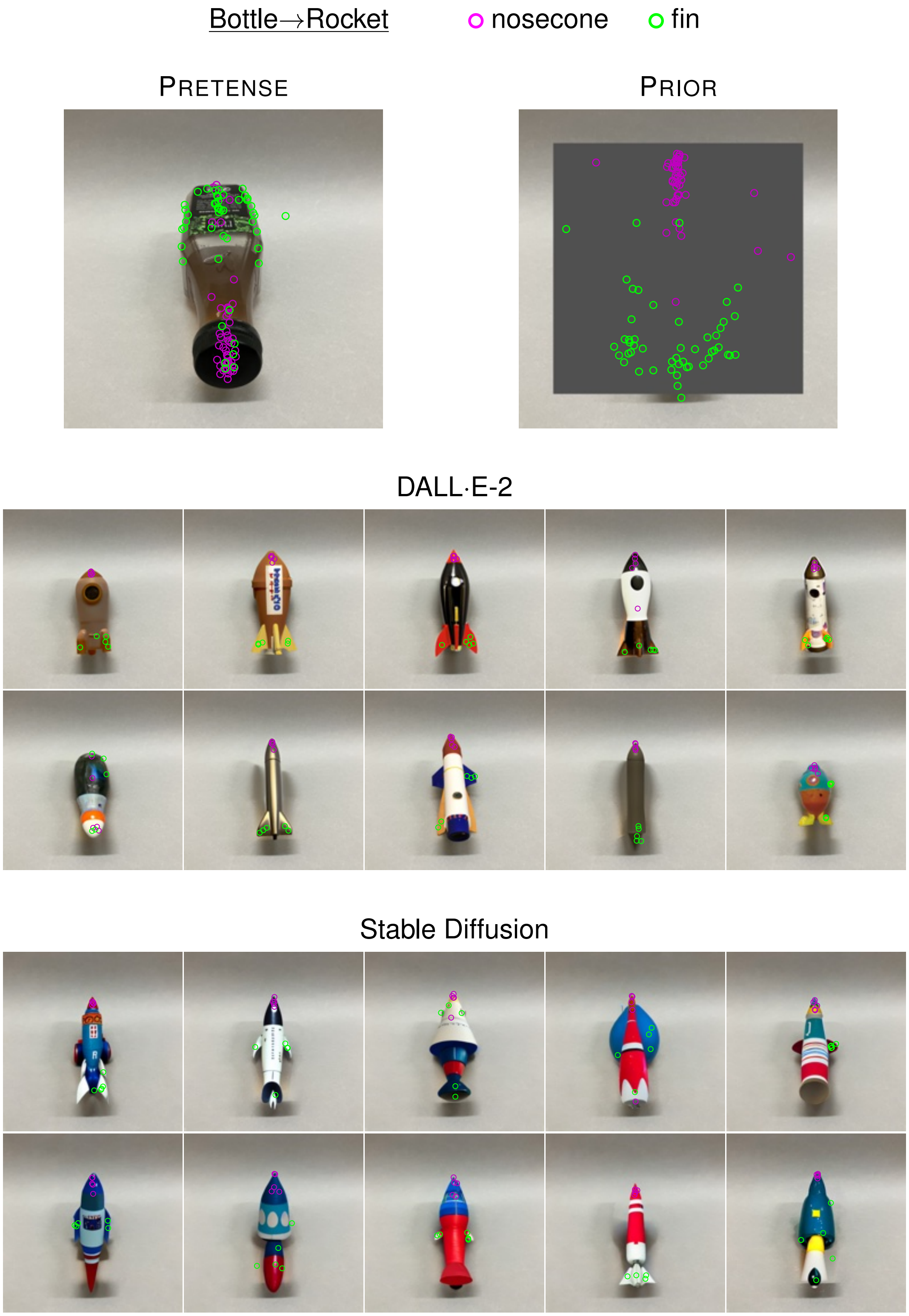

Figure S178: People's responses in the PRETENSE and PRIOR conditions, as well as coding of model-inpainted images. Participants were prompted to pretend the bottle is a rocket. Models inpainted the masked area of the bottle given the prompt "A rocket".

References

Radford, A., Kim, J. W., Hallacy, C., Ramesh, A., Goh, G., Agarwal, S., Sastry, G., Askell, A., Mishkin, P., Clark, J., et al. (2021). Learning transferable visual models from natural language supervision. *International conference on machine learning*, 8748–8763.

Ramesh, A., Dhariwal, P., Nichol, A., Chu, C., & Chen, M. (2022). Hierarchical text-conditional image generation with clip latents. *arXiv e-prints*, arXiv–2204.

Rombach, R., Blattmann, A., Lorenz, D., Esser, P., & Ommer, B. (2022). High-resolution image synthesis with latent diffusion models. *Proceedings of the IEEE/CVF conference on computer vision and pattern recognition*, 10684–10695.
